# Supplementary material for: Accelerated phosphatidylcholine turnover in macrophages promotes adipose tissue inflammation in obesity
Source: eLife. 2019 Aug 16;8:e47990. doi: 10.7554/eLife.47990 (PMC6748830; doi:10.7554/eLife.47990)
Supplement: Supplementary file 1. [file elife-47990-supp1.docx]

**Supplementary file 1.**

| **name** | **OB vs WT log.odds.ratio** | **OB vs WT adjusted.p.value** | **GO ID** |
| --- | --- | --- | --- |
| RNA localization | 0.109695705 | 6.08E-06 | GO:0006403 |
| microtubule cytoskeleton organization | 0.093090767 | 6.50E-06 | GO:0000226 |
| chromatin assembly or disassembly | 0.072715827 | 1.00E-05 | GO:0006333 |
| microtubule-based movement | 0.090059298 | 2.33E-05 | GO:0007018 |
| microtubule organizing center organization | 0.146245681 | 2.61E-05 | GO:0031023 |
| mRNA transport | 0.109808548 | 3.59E-05 | GO:0051028 |
| regulation of DNA replication | 0.138664509 | 4.54E-05 | GO:0006275 |
| centrosome organization | 0.144335312 | 8.12E-05 | GO:0051297 |
| nucleobase, nucleoside, nucleotide and nucleic acid transport | 0.099854207 | 0.000111184 | GO:0015931 |
| mRNA processing | 0.0578393 | 0.000167041 | GO:0006397 |
| mRNA metabolic process | 0.054040069 | 0.000340716 | GO:0016071 |
| regulation of DNA metabolic process | 0.096223508 | 0.000421682 | GO:0051052 |
| nuclear export | 0.114899289 | 0.000538998 | GO:0051168 |
| ER to Golgi vesicle-mediated transport | 0.123039145 | 0.000781143 | GO:0006888 |
| sphingolipid metabolic process | 0.100679526 | 0.000805755 | GO:0006665 |
| cytoskeleton-dependent intracellular transport | 0.076563482 | 0.00088951 | GO:0030705 |
| mitochondrial transport | 0.100785069 | 0.000903336 | GO:0006839 |
| negative regulation of adaptive immune response | 0.141384187 | 0.000996114 | GO:0002820 |
| negative regulation of adaptive immune response based on somatic recombination of immune receptors built from immunoglobulin superfamily domains | 0.141384187 | 0.000996114 | GO:0002823 |
| spindle organization | 0.136044423 | 0.000996578 | GO:0007051 |
| proteasomal protein catabolic process | 0.116160699 | 0.001202692 | GO:0010498 |
| proteasomal ubiquitin-dependent protein catabolic process | 0.116160699 | 0.001202692 | GO:0043161 |
| tetrapyrrole metabolic process | 0.118690789 | 0.001216214 | GO:0033013 |
| negative regulation of leukocyte mediated immunity | 0.138596463 | 0.001265756 | GO:0002704 |
| negative regulation of lymphocyte mediated immunity | 0.138596463 | 0.001265756 | GO:0002707 |
| porphyrin metabolic process | 0.118893495 | 0.001478977 | GO:0006778 |
| pigment metabolic process | 0.10813916 | 0.001521217 | GO:0042440 |
| centrosome duplication | 0.150989536 | 0.001774733 | GO:0051298 |
| regulation of microtubule-based process | 0.103843338 | 0.002239439 | GO:0032886 |
| membrane lipid metabolic process | 0.063257735 | 0.002637924 | GO:0006643 |
| B cell homeostasis | 0.128008324 | 0.002648853 | GO:0001782 |
| positive regulation of endocytosis | 0.12047554 | 0.002907785 | GO:0045807 |
| regulation of phagocytosis | 0.128076246 | 0.003712153 | GO:0050764 |
| negative regulation of immune effector process | 0.124974473 | 0.004112746 | GO:0002698 |
| posttranscriptional regulation of gene expression | 0.075584143 | 0.004112746 | GO:0010608 |
| negative regulation of immune response | 0.118078381 | 0.00413245 | GO:0050777 |
| protein import into nucleus, docking | 0.132229562 | 0.004191572 | GO:0000059 |
| phagocytosis | 0.101446635 | 0.00503594 | GO:0006909 |
| cellular lipid catabolic process | 0.093169711 | 0.00503594 | GO:0044242 |
| myeloid leukocyte activation | 0.105316074 | 0.006950655 | GO:0002274 |
| regulation of mitosis | 0.106515089 | 0.007362457 | GO:0007088 |
| cofactor catabolic process | 0.11107813 | 0.007622515 | GO:0051187 |
| positive regulation of phagocytosis | 0.128128292 | 0.007785139 | GO:0050766 |
| natural killer cell mediated immunity | 0.119540819 | 0.008671944 | GO:0002228 |
| natural killer cell mediated cytotoxicity | 0.119540819 | 0.008671944 | GO:0042267 |
| RNA splicing | 0.051881202 | 0.008977949 | GO:0008380 |
| protein ubiquitination | 0.079124981 | 0.00903524 | GO:0016567 |
| phospholipid biosynthetic process | 0.084888604 | 0.009600299 | GO:0008654 |
| protein export from nucleus | 0.130373098 | 0.009827841 | GO:0006611 |
| regulation of mitotic metaphase/anaphase transition | 0.140486196 | 0.010926482 | GO:0030071 |
| protein modification by small protein conjugation | 0.07415241 | 0.012375254 | GO:0032446 |
| establishment or maintenance of chromatin architecture | 0.04324993 | 0.012884899 | GO:0006325 |
| membrane lipid biosynthetic process | 0.07747481 | 0.013692795 | GO:0046467 |
| amino acid transport | 0.082876543 | 0.017506793 | GO:0006865 |
| homophilic cell adhesion | 0.072883396 | 0.020657461 | GO:0007156 |
| meiosis I | 0.1025323 | 0.022072268 | GO:0007127 |
| sterol transport | 0.117152176 | 0.025155824 | GO:0015918 |
| cholesterol transport | 0.117152176 | 0.025155824 | GO:0030301 |
| glycerophospholipid metabolic process | 0.077396853 | 0.025221219 | GO:0006650 |
| coenzyme catabolic process | 0.107467379 | 0.031451225 | GO:0009109 |
| macroautophagy | 0.124851577 | 0.03246804 | GO:0016236 |
| immune response-regulating cell surface receptor signaling pathway | 0.092804257 | 0.035270204 | GO:0002768 |
| tricarboxylic acid cycle | 0.114235727 | 0.035778842 | GO:0006099 |
| posttranscriptional gene silencing | 0.125397712 | 0.041310187 | GO:0016441 |
| posttranscriptional gene silencing by RNA | 0.125397712 | 0.041310187 | GO:0035194 |
| regulation of endocytosis | 0.091158719 | 0.044384454 | GO:0030100 |
| regulation of translation | 0.076317448 | 0.044950188 | GO:0006417 |
| embryonic cleavage | 0.136020547 | 0.046564678 | GO:0040016 |
| immune response-activating signal transduction | 0.089300606 | 0.04715384 | GO:0002757 |
| acetyl-CoA catabolic process | 0.111487385 | 0.048713909 | GO:0046356 |
| DNA damage response, signal transduction | 0.086685282 | 0.04960336 | GO:0042770 |
