# Supplementary material for: Accelerated phosphatidylcholine turnover in macrophages promotes adipose tissue inflammation in obesity
Source: eLife. 2019 Aug 16;8:e47990. doi: 10.7554/eLife.47990 (PMC6748830; doi:10.7554/eLife.47990)
Supplement: Supplementary file 2. — Supplementary file 2b. The list of differentially expressed genes in eWAT isolated from Lepob/ob BMT Pcyt1afl/fl and Pcyt1afl/flLyz2Cre/+ mice, ranked in ascending order of p value. [file elife-47990-supp2.docx]

**SupSuplementary file 2a.**

| **Pathway/term** | **Direction** | **statistic** | **p.value** |
| --- | --- | --- | --- |
| insulin receptor signaling pathway | UP | 3.130495169 | 0.000582751 |
| circadian rhythm | UP | 3.130495169 | 0.000582751 |
| cellular response to growth factor stimulus | UP | 2.874944543 | 0.002331002 |
| positive regulation of phosphatidylinositol 3-kinase signaling | DOWN | -2.874944543 | 0.002331002 |
| cellular response to zinc ion | UP | 2.74716923 | 0.004079254 |
| extracellular matrix constituent secretion | UP | 2.74716923 | 0.004079254 |
| positive regulation of transcription from RNA polymerase II promoter in response to endoplasmic reticulum stress | UP | 2.74716923 | 0.004079254 |
| negative regulation of endoplasmic reticulum stress-induced intrinsic apoptotic signaling pathway | UP | 2.74716923 | 0.004079254 |
| regulation of necrotic cell death | DOWN | -2.74716923 | 0.004079254 |
| cellular response to insulin stimulus | UP | 2.619393917 | 0.006993007 |
| mRNA transcription from RNA polymerase II promoter | UP | 2.619393917 | 0.006993007 |
| negative regulation of BMP signaling pathway | UP | 2.619393917 | 0.006993007 |
| protein processing | DOWN | -2.619393917 | 0.006993007 |
| brain-derived neurotrophic factor receptor signaling pathway | UP | 2.491618604 | 0.011072261 |
| regulation of GTPase activity | UP | 2.491618604 | 0.011072261 |
| intracellular signal transduction | UP | 2.491618604 | 0.011072261 |
| IRES-dependent translational initiation | UP | 2.491618604 | 0.011072261 |
| negative regulation of translational initiation | UP | 2.491618604 | 0.011072261 |
| inner ear development | DOWN | -2.491618604 | 0.011072261 |
| positive regulation of fever generation | DOWN | -2.491618604 | 0.011072261 |
| positive regulation of tumor necrosis factor-mediated signaling pathway | DOWN | -2.491618604 | 0.011072261 |
| positive regulation of I-kappaB phosphorylation | DOWN | -2.491618604 | 0.011072261 |
| regulation of JNK cascade | DOWN | -2.491618604 | 0.011072261 |
| cellular protein complex assembly | DOWN | -2.491618604 | 0.011072261 |
| regulation of protein phosphorylation | DOWN | -2.491618604 | 0.011072261 |
| negative regulation of cytokine secretion involved in immune response | DOWN | -2.491618604 | 0.011072261 |
| glomerulus vasculature development | DOWN | -2.491618604 | 0.011072261 |
| regulation of macrophage migration inhibitory factor signaling pathway | DOWN | -2.491618604 | 0.011072261 |
| regulation of tumor necrosis factor production | DOWN | -2.491618604 | 0.011072261 |
| negative regulation of vascular permeability | DOWN | -2.491618604 | 0.011072261 |
| cell-substrate adhesion | DOWN | -2.491618604 | 0.011072261 |
| negative regulation of protein import into nucleus | DOWN | -2.491618604 | 0.011072261 |
| regulation of protein binding | DOWN | -2.491618604 | 0.011072261 |
| negative regulation of protein phosphorylation | DOWN | -2.491618604 | 0.011072261 |
| negative regulation of cell-substrate adhesion | DOWN | -2.491618604 | 0.011072261 |
| fructose 6-phosphate metabolic process | UP | 2.491618604 | 0.011072261 |
| negative regulation of insulin secretion | UP | 2.491618604 | 0.011072261 |
| glycolytic process through fructose-6-phosphate | UP | 2.491618604 | 0.011072261 |
| response to glucose | UP | 2.491618604 | 0.011072261 |
| malonyl-CoA catabolic process | UP | 2.491618604 | 0.011072261 |
| positive regulation of fatty acid oxidation | UP | 2.491618604 | 0.011072261 |
| regulation of establishment or maintenance of cell polarity | DOWN | -2.491618604 | 0.011072261 |
| multicellular organism growth | DOWN | -2.491618604 | 0.011072261 |
| prostate gland growth | DOWN | -2.491618604 | 0.011072261 |
| insulin-like growth factor receptor signaling pathway | DOWN | -2.491618604 | 0.011072261 |
| chondroitin sulfate proteoglycan biosynthetic process | DOWN | -2.491618604 | 0.011072261 |
| antigen receptor-mediated signaling pathway | UP | 2.491618604 | 0.011072261 |
| neutrophil apoptotic process | UP | 2.491618604 | 0.011072261 |
| negative regulation of chemokine biosynthetic process | UP | 2.491618604 | 0.011072261 |
| negative regulation of hormone secretion | UP | 2.491618604 | 0.011072261 |
| positive regulation of T-helper 2 cell differentiation | UP | 2.491618604 | 0.011072261 |
| branching involved in salivary gland morphogenesis | UP | 2.491618604 | 0.011072261 |
| cellular response to interleukin-1 | UP | 2.491618604 | 0.011072261 |
| cellular response to tumor necrosis factor | UP | 2.491618604 | 0.011072261 |
| positive regulation of neuron projection development | UP | 2.363843291 | 0.017482518 |
| nerve growth factor signaling pathway | UP | 2.363843291 | 0.017482518 |
| cellular response to nerve growth factor stimulus | UP | 2.363843291 | 0.017482518 |
| positive regulation of synapse assembly | UP | 2.363843291 | 0.017482518 |
| nervous system development | UP | 2.363843291 | 0.017482518 |
| negative regulation of anoikis | UP | 2.363843291 | 0.017482518 |
| cellular response to organic substance | DOWN | -2.363843291 | 0.017482518 |
| cellular response to amino acid starvation | UP | 2.363843291 | 0.017482518 |
| regulation of microtubule-based movement | UP | 2.363843291 | 0.017482518 |
| extracellular matrix organization | DOWN | -2.363843291 | 0.017482518 |
| interleukin-6-mediated signaling pathway | UP | 2.363843291 | 0.017482518 |
| inositol trisphosphate biosynthetic process | DOWN | -2.363843291 | 0.017482518 |
| tumor necrosis factor-mediated signaling pathway | DOWN | -2.363843291 | 0.017482518 |
| cellular extravasation | DOWN | -2.363843291 | 0.017482518 |
| positive regulation of translational initiation by iron | DOWN | -2.363843291 | 0.017482518 |
| regulation of branching involved in salivary gland morphogenesis | DOWN | -2.363843291 | 0.017482518 |
| negative regulation of alkaline phosphatase activity | DOWN | -2.363843291 | 0.017482518 |
| negative regulation of glucose import | DOWN | -2.363843291 | 0.017482518 |
| regulation of protein secretion | DOWN | -2.363843291 | 0.017482518 |
| regulation of reactive oxygen species metabolic process | DOWN | -2.363843291 | 0.017482518 |
| defense response to bacterium | DOWN | -2.363843291 | 0.017482518 |
| negative regulation of NF-kappaB transcription factor activity | DOWN | -2.363843291 | 0.017482518 |
| hydrogen peroxide catabolic process | DOWN | -2.363843291 | 0.017482518 |
| protein acetylation | UP | 2.363843291 | 0.017482518 |
| positive regulation of transcription from RNA polymerase II promoter involved in unfolded protein response | UP | 2.363843291 | 0.017482518 |
| protein stabilization | UP | 2.363843291 | 0.017482518 |
| release of sequestered calcium ion into cytosol by sarcoplasmic reticulum | UP | 2.363843291 | 0.017482518 |
| response to caffeine | UP | 2.363843291 | 0.017482518 |
| cellular response to caffeine | UP | 2.363843291 | 0.017482518 |
| growth hormone secretion | UP | 2.363843291 | 0.017482518 |
| positive regulation of tyrosine phosphorylation of Stat5 protein | UP | 2.363843291 | 0.017482518 |
| programmed necrotic cell death | DOWN | -2.363843291 | 0.017482518 |
| positive regulation of innate immune response | DOWN | -2.363843291 | 0.017482518 |
| regulation of blood pressure | DOWN | -2.363843291 | 0.017482518 |
| response to organic substance | DOWN | -2.236067978 | 0.026223776 |
| cellular response to lipopolysaccharide | UP | 2.236067978 | 0.026223776 |
| epidermal growth factor receptor signaling pathway | UP | 2.236067978 | 0.026223776 |
| regulation of multicellular organism growth | UP | 2.236067978 | 0.026223776 |
| JAK-STAT cascade | UP | 2.236067978 | 0.026223776 |
| positive regulation of interleukin-6 production | DOWN | -2.236067978 | 0.026223776 |
| organ induction | UP | 2.236067978 | 0.026223776 |
| positive regulation of epithelial cell proliferation | UP | 2.236067978 | 0.026223776 |
| leukocyte migration | DOWN | -2.236067978 | 0.026223776 |
| positive regulation of JNK cascade | DOWN | -2.236067978 | 0.026223776 |
| response to molecule of bacterial origin | DOWN | -2.236067978 | 0.026223776 |
| positive regulation of protein kinase B signaling | DOWN | -2.236067978 | 0.026223776 |
| positive regulation of cerebellar granule cell precursor proliferation | DOWN | -2.236067978 | 0.026223776 |
| glucose homeostasis | UP | 2.236067978 | 0.026223776 |
| activation of cysteine-type endopeptidase activity involved in apoptotic process by cytochrome c | DOWN | -2.236067978 | 0.026223776 |
| positive regulation of extrinsic apoptotic signaling pathway in absence of ligand | DOWN | -2.236067978 | 0.026223776 |
| positive regulation of axon regeneration | UP | 2.236067978 | 0.026223776 |
| early endosome to late endosome transport | DOWN | -2.236067978 | 0.026223776 |
| muscle fiber development | DOWN | -2.236067978 | 0.026223776 |
| collagen catabolic process | DOWN | -2.236067978 | 0.026223776 |
| B cell receptor signaling pathway | UP | 2.108292665 | 0.037878788 |
| platelet-derived growth factor receptor-alpha signaling pathway | DOWN | -2.108292665 | 0.037878788 |
| positive regulation of chemotaxis | DOWN | -2.108292665 | 0.037878788 |
| positive regulation of MAP kinase activity | DOWN | -2.108292665 | 0.037878788 |
| apoptotic process | DOWN | -2.108292665 | 0.037878788 |
| vesicle targeting | DOWN | -2.108292665 | 0.037878788 |
| liver regeneration | DOWN | -2.108292665 | 0.037878788 |
| embryo implantation | DOWN | -2.108292665 | 0.037878788 |
| proteolysis | DOWN | -2.108292665 | 0.037878788 |
| angiogenesis | UP | 2.108292665 | 0.037878788 |
| activation of MAPK activity | UP | 2.108292665 | 0.037878788 |
| cellular response to glucose stimulus | DOWN | -2.108292665 | 0.037878788 |
| brown fat cell differentiation | UP | 2.108292665 | 0.037878788 |
| negative regulation of fibroblast proliferation | UP | 2.108292665 | 0.037878788 |
| white fat cell differentiation | UP | 2.108292665 | 0.037878788 |
| positive regulation of establishment of protein localization to plasma membrane | DOWN | -2.108292665 | 0.037878788 |
| protein localization to cell surface | DOWN | -2.108292665 | 0.037878788 |
| embryonic hemopoiesis | UP | 2.108292665 | 0.037878788 |
| movement of cell or subcellular component | UP | 2.108292665 | 0.037878788 |

**Supplementary file 2b.**

| **ENSEMBL ID** | **Symbol** | **log Foldchange** | **logCPM** | **PValue** |
| --- | --- | --- | --- | --- |
| ENSMUSG00000000982 | Ccl3 | -0.784425382 | 4.075936345 | 4.34181E-12 |
| ENSMUSG00000032532 | Cck | 0.779315627 | 3.577727084 | 2.67968E-07 |
| ENSMUSG00000028862 | Map3k6 | 0.503954106 | 5.352963464 | 3.8662E-07 |
| ENSMUSG00000022303 | Dcstamp | -0.589784244 | 4.810417524 | 8.164E-07 |
| ENSMUSG00000004891 | Nes | -0.387582116 | 7.408291243 | 1.00766E-06 |
| ENSMUSG00000042485 | Mustn1 | 0.462251704 | 5.091396832 | 1.06183E-06 |
| ENSMUSG00000036040 | Adamtsl2 | 0.66221741 | 3.518338474 | 3.75892E-06 |
| ENSMUSG00000046694 | Tent5b | 0.8304418 | 1.316960417 | 4.23222E-06 |
| ENSMUSG00000026437 | Cdk18 | -0.351007935 | 8.015685055 | 4.41892E-06 |
| ENSMUSG00000003534 | Ddr1 | 0.358839694 | 5.376826201 | 4.58003E-06 |
| ENSMUSG00000021701 | Plk2 | -0.277497352 | 7.294222164 | 5.11482E-06 |
| ENSMUSG00000038515 | Grtp1 | 0.560220261 | 3.516346468 | 6.14235E-06 |
| ENSMUSG00000027488 | Snta1 | 0.412561556 | 5.798005381 | 6.39429E-06 |
| ENSMUSG00000066687 | Zbtb16 | 0.97664784 | 3.110912893 | 7.95838E-06 |
| ENSMUSG00000026822 | Lcn2 | 0.562786248 | 6.410678715 | 1.26895E-05 |
| ENSMUSG00000085081 | Rptoros | -0.722837973 | 1.466362849 | 1.32753E-05 |
| ENSMUSG00000001270 | Ckb | -0.380604558 | 9.023736177 | 1.40367E-05 |
| ENSMUSG00000040026 | Saa3 | 0.582369491 | 5.742314449 | 1.73176E-05 |
| ENSMUSG00000026358 | Rgs1 | -0.326421688 | 6.768222787 | 2.05892E-05 |
| ENSMUSG00000022505 | Emp2 | -0.375501542 | 5.211639085 | 2.21263E-05 |
| ENSMUSG00000028459 | Cd72 | -0.309807323 | 7.08064965 | 2.27706E-05 |
| ENSMUSG00000090084 | Srpx | -0.373953817 | 4.867496038 | 2.89136E-05 |
| ENSMUSG00000021798 | Ldb3 | 0.579989493 | 2.793039244 | 3.0024E-05 |
| ENSMUSG00000029819 | Npy | -0.742796877 | 3.260914464 | 3.37112E-05 |
| ENSMUSG00000030317 | Timp4 | 0.798611223 | 6.077147685 | 3.80027E-05 |
| ENSMUSG00000021904 | Sema3g | 0.376415246 | 5.759942901 | 4.32712E-05 |
| ENSMUSG00000028111 | Ctsk | -0.347697615 | 9.4034626 | 4.59372E-05 |
| ENSMUSG00000040852 | Plekhh2 | 3.522916693 | 6.988701094 | 4.71473E-05 |
| ENSMUSG00000025902 | Sox17 | 0.436882514 | 3.917407795 | 5.45815E-05 |
| ENSMUSG00000068699 | Flnc | -0.393061457 | 6.182313119 | 5.6015E-05 |
| ENSMUSG00000026207 | Speg | -0.280515974 | 6.146048124 | 6.35889E-05 |
| ENSMUSG00000037907 | Ankrd13b | -0.377633043 | 5.676842424 | 6.62088E-05 |
| ENSMUSG00000061100 | Retnla | 1.127991478 | 3.470739129 | 6.65671E-05 |
| ENSMUSG00000029368 | Alb | 3.006425661 | 3.622267834 | 6.75982E-05 |
| ENSMUSG00000038167 | Plekhg6 | 0.743549069 | 2.289223375 | 8.05109E-05 |
| ENSMUSG00000085151 | 1110018N20Rik | 0.724514316 | 1.150653438 | 8.28947E-05 |
| ENSMUSG00000032060 | Cryab | 0.376018097 | 7.941793298 | 8.50844E-05 |
| ENSMUSG00000000957 | Mmp14 | -0.294584701 | 8.29047699 | 8.72806E-05 |
| ENSMUSG00000021306 | Gpr137b | -0.285387198 | 6.928822211 | 8.73668E-05 |
| ENSMUSG00000033585 | Ndn | 0.330975335 | 4.372940894 | 9.4222E-05 |
| ENSMUSG00000030246 | Ldhb | 0.399716174 | 4.023435858 | 9.72419E-05 |
| ENSMUSG00000029816 | Gpnmb | -0.371159057 | 12.70385677 | 9.84939E-05 |
| ENSMUSG00000049719 | Prss46 | -0.596213672 | 2.850106958 | 0.000102376 |
| ENSMUSG00000009394 | Syn2 | 0.551740184 | 2.116481202 | 0.00010557 |
| ENSMUSG00000079012 | Serpina3m | 0.554282001 | 2.473342351 | 0.00010834 |
| ENSMUSG00000014773 | Dll1 | 0.51760771 | 2.804053668 | 0.000115691 |
| ENSMUSG00000032020 | Ubash3b | -0.25868549 | 5.553664351 | 0.000132787 |
| ENSMUSG00000024084 | Qpct | -0.366930966 | 6.493847573 | 0.000135297 |
| ENSMUSG00000026981 | Il1rn | -0.558588706 | 6.341660095 | 0.000137893 |
| ENSMUSG00000078532 | Nkain1 | -0.396525946 | 3.310147312 | 0.000143307 |
| ENSMUSG00000005681 | Apoa2 | 2.788327032 | 1.495662339 | 0.000146931 |
| ENSMUSG00000028195 | Ccn1 | 0.390440186 | 5.872220198 | 0.000151024 |
| ENSMUSG00000029330 | Cds1 | 0.314206208 | 4.331514854 | 0.000153514 |
| ENSMUSG00000041688 | Amot | -0.560654605 | 2.407282504 | 0.000155798 |
| ENSMUSG00000029716 | Tfr2 | 0.548774301 | 5.549268535 | 0.000164998 |
| ENSMUSG00000040564 | Apoc1 | 0.455488984 | 6.052771861 | 0.000166435 |
| ENSMUSG00000024998 | Plce1 | 0.386865376 | 3.703947717 | 0.00017477 |
| ENSMUSG00000036913 | Trim67 | 0.537196863 | 3.052739442 | 0.000177149 |
| ENSMUSG00000025610 | Map3k7cl | 0.668298987 | 1.27539375 | 0.000181265 |
| ENSMUSG00000051906 | Cd209f | 1.036262795 | 0.881607268 | 0.000182903 |
| ENSMUSG00000031980 | Agt | 0.459536663 | 5.140908588 | 0.000184942 |
| ENSMUSG00000027412 | Lpin3 | 0.48208629 | 3.555401586 | 0.000192964 |
| ENSMUSG00000079049 | Serpinb1c | -0.521935281 | 2.921998521 | 0.000194361 |
| ENSMUSG00000102752 | Gm7694 | 0.449531461 | 4.894865618 | 0.000209114 |
| ENSMUSG00000039004 | Bmp6 | 0.376409099 | 3.604378845 | 0.000211865 |
| ENSMUSG00000015112 | Slc25a13 | -0.272006772 | 4.719668864 | 0.00021444 |
| ENSMUSG00000030774 | Pak1 | -0.291505032 | 5.775420222 | 0.000217188 |
| ENSMUSG00000018930 | Ccl4 | -0.545634234 | 3.166071203 | 0.000217239 |
| ENSMUSG00000050052 | Tdrp | 0.310262581 | 5.552443205 | 0.000217487 |
| ENSMUSG00000031722 | Hp | 0.381697991 | 12.96692065 | 0.000219249 |
| ENSMUSG00000003526 | Prodh | 0.381002172 | 3.997593493 | 0.000223667 |
| ENSMUSG00000022421 | Nptxr | -0.293565875 | 3.910077364 | 0.000232594 |
| ENSMUSG00000033933 | Vhl | 0.305420642 | 5.133257344 | 0.000233029 |
| ENSMUSG00000056380 | Gpr50 | 0.533202337 | 6.191103086 | 0.000234494 |
| ENSMUSG00000052889 | Prkcb | -0.391801455 | 5.34219388 | 0.000240434 |
| ENSMUSG00000025498 | Irf7 | 0.456706599 | 5.832100228 | 0.000256828 |
| ENSMUSG00000051855 | Mest | 0.414687211 | 8.088656879 | 0.000305886 |
| ENSMUSG00000019820 | Utrn | 0.271424415 | 5.545247009 | 0.000306269 |
| ENSMUSG00000056091 | St3gal5 | -0.314162989 | 4.495713966 | 0.000310259 |
| ENSMUSG00000084883 | Ccdc85c | 0.392409254 | 3.030551206 | 0.000317481 |
| ENSMUSG00000028341 | Nr4a3 | 0.695631891 | 0.986701983 | 0.0003332 |
| ENSMUSG00000032047 | Acat1 | 0.246366585 | 6.903140089 | 0.00034227 |
| ENSMUSG00000029084 | Cd38 | 0.427500981 | 3.602101296 | 0.000344285 |
| ENSMUSG00000036534 | Slc38a7 | -0.210763112 | 5.877644466 | 0.000349418 |
| ENSMUSG00000031616 | Ednra | -0.317411232 | 4.344614432 | 0.00035064 |
| ENSMUSG00000078763 | Slfn1 | 0.58894037 | 1.941993647 | 0.000354442 |
| ENSMUSG00000058624 | Gda | 0.300413091 | 6.318169016 | 0.000355713 |
| ENSMUSG00000006445 | Epha2 | 0.372291927 | 3.27112893 | 0.000363321 |
| ENSMUSG00000090952 | Gm17251 | 0.378852677 | 3.525155645 | 0.000371018 |
| ENSMUSG00000039323 | Igfbp2 | 0.847042539 | 2.098428278 | 0.000372718 |
| ENSMUSG00000068923 | Syt11 | -0.282135796 | 4.661363876 | 0.000377845 |
| ENSMUSG00000022181 | C6 | 0.469718004 | 4.14573956 | 0.00038123 |
| ENSMUSG00000028937 | Acot7 | -0.270812914 | 6.615229792 | 0.000384722 |
| ENSMUSG00000055632 | Hmcn2 | 0.60360655 | 5.80352067 | 0.000392689 |
| ENSMUSG00000022018 | Rgcc | 0.365136044 | 6.64530102 | 0.000398659 |
| ENSMUSG00000069135 | Fgfr1op | 0.337270237 | 3.570024974 | 0.000404185 |
| ENSMUSG00000015468 | Notch4 | 0.401415058 | 5.357721308 | 0.000405938 |
| ENSMUSG00000030711 | Sult1a1 | 0.390136539 | 6.188796541 | 0.000410195 |
| ENSMUSG00000019122 | Ccl9 | -0.313964859 | 9.341443111 | 0.000411493 |
| ENSMUSG00000103847 | Gm20056 | -0.40208216 | 3.907543336 | 0.000435239 |
| ENSMUSG00000029254 | Stap1 | -0.337284706 | 5.595286103 | 0.00044465 |
| ENSMUSG00000032401 | Lctl | 0.369310556 | 6.62811493 | 0.000446447 |
| ENSMUSG00000021411 | Pxdc1 | 0.312825834 | 4.163724255 | 0.000463026 |
| ENSMUSG00000021822 | Plau | -0.290685186 | 8.461259662 | 0.00046322 |
| ENSMUSG00000001918 | Slc1a5 | 0.33187596 | 9.492509435 | 0.000475766 |
| ENSMUSG00000025036 | Sfxn2 | 0.255862911 | 4.499473005 | 0.000488434 |
| ENSMUSG00000090394 | 4930523C07Rik | 0.291570148 | 4.776334371 | 0.000491656 |
| ENSMUSG00000060594 | Layn | -0.470025651 | 5.206305495 | 0.000492036 |
| ENSMUSG00000026979 | Psd4 | -0.231900206 | 6.318352966 | 0.000492344 |
| ENSMUSG00000033540 | Idua | -0.226434083 | 5.811185493 | 0.00049731 |
| ENSMUSG00000009654 | Oit3 | -0.438091148 | 3.484066192 | 0.000515981 |
| ENSMUSG00000034738 | Nostrin | -0.271761297 | 4.526087228 | 0.000518907 |
| ENSMUSG00000023092 | Fhl1 | 0.376963222 | 6.876251645 | 0.00051953 |
| ENSMUSG00000025473 | Adam8 | -0.25521892 | 10.10169044 | 0.000544423 |
| ENSMUSG00000024770 | Lipn | -0.652142523 | 1.142637716 | 0.000552336 |
| ENSMUSG00000048126 | Col6a3 | -0.277049638 | 8.957693171 | 0.00056712 |
| ENSMUSG00000031266 | Gla | -0.207139671 | 6.612308403 | 0.000575354 |
| ENSMUSG00000032125 | Robo4 | 0.341704116 | 5.936200968 | 0.000580562 |
| ENSMUSG00000021771 | Vdac2 | -0.195409709 | 7.952631659 | 0.000584042 |
| ENSMUSG00000078234 | Klhdc7a | 0.426525882 | 6.184384908 | 0.000587188 |
| ENSMUSG00000031442 | Mcf2l | 0.29567571 | 5.018780589 | 0.000595792 |
| ENSMUSG00000049723 | Mmp12 | -0.488333468 | 11.22299343 | 0.000615437 |
| ENSMUSG00000003283 | Hck | -0.219519669 | 6.835403154 | 0.000652677 |
| ENSMUSG00000022836 | Mylk | 0.412281551 | 6.319003105 | 0.000654957 |
| ENSMUSG00000036611 | Eepd1 | 0.383022346 | 7.004268965 | 0.000675931 |
| ENSMUSG00000035914 | Cd276 | -0.259753962 | 4.803631748 | 0.000697572 |
| ENSMUSG00000039062 | Anpep | -0.251973 | 9.697330881 | 0.000705876 |
| ENSMUSG00000024074 | Crim1 | 0.30452732 | 4.773358266 | 0.000706385 |
| ENSMUSG00000097993 | Ptprv | -0.510986041 | 2.210950781 | 0.00071948 |
| ENSMUSG00000024401 | Tnf | -0.364895377 | 3.529615362 | 0.000723006 |
| ENSMUSG00000030342 | Cd9 | -0.262725273 | 8.724585822 | 0.000728595 |
| ENSMUSG00000036103 | Colec12 | -0.253325373 | 5.91456787 | 0.000731321 |
| ENSMUSG00000026621 | Marc1 | 0.432546795 | 5.944191384 | 0.000735348 |
| ENSMUSG00000035686 | Thrsp | 0.514709896 | 6.510367048 | 0.00077258 |
| ENSMUSG00000030088 | Aldh1l1 | 0.396496244 | 7.274191974 | 0.000776078 |
| ENSMUSG00000045954 | Cavin2 | 0.313273167 | 7.712030199 | 0.000779628 |
| ENSMUSG00000062082 | Cd200r4 | -0.341360083 | 3.292569995 | 0.000782574 |
| ENSMUSG00000024222 | Fkbp5 | 0.459864765 | 5.824375764 | 0.000789708 |
| ENSMUSG00000041734 | Kirrel | 0.284044473 | 6.103083318 | 0.000808511 |
| ENSMUSG00000033526 | Ppip5k1 | 0.253070081 | 5.791025062 | 0.000828732 |
| ENSMUSG00000020312 | Shc2 | -0.245823431 | 5.498502119 | 0.000840725 |
| ENSMUSG00000045659 | Plekha7 | 0.425138434 | 4.221585751 | 0.000855653 |
| ENSMUSG00000033581 | Igf2bp2 | -0.291643198 | 6.269718764 | 0.000861977 |
| ENSMUSG00000031119 | Gpc4 | 0.343507802 | 5.093227796 | 0.000863437 |
| ENSMUSG00000029335 | Bmp3 | 0.320421898 | 5.180385729 | 0.000878308 |
| ENSMUSG00000019718 | L3hypdh | 0.398269815 | 3.609394817 | 0.000879893 |
| ENSMUSG00000027332 | Ivd | 0.219468019 | 6.600470465 | 0.000881719 |
| ENSMUSG00000019997 | Ccn2 | 0.797276673 | 5.04078003 | 0.000896181 |
| ENSMUSG00000037493 | Cib2 | 0.279234182 | 6.542744142 | 0.000929677 |
| ENSMUSG00000021451 | Sema4d | -0.280389452 | 6.952334554 | 0.000930909 |
| ENSMUSG00000018830 | Myh11 | 0.342344836 | 5.17328153 | 0.000940371 |
| ENSMUSG00000073565 | Prr16 | 0.464185091 | 3.100927469 | 0.000963296 |
| ENSMUSG00000025347 | Mettl7b | 0.584454955 | 2.796964186 | 0.000969925 |
| ENSMUSG00000052565 | Hist1h1d | -0.820981323 | 0.336115524 | 0.000971919 |
| ENSMUSG00000048218 | Amigo2 | 0.605194333 | 2.648749685 | 0.000985629 |
| ENSMUSG00000029762 | Akr1b8 | -0.410536657 | 8.962709478 | 0.000986143 |
| ENSMUSG00000022419 | Deptor | 0.274662434 | 6.16372149 | 0.001000266 |
| ENSMUSG00000027956 | Tmem144 | -0.370192578 | 3.160301917 | 0.001007124 |
| ENSMUSG00000109508 | Gm44956 | 0.709442594 | 0.617671723 | 0.001041603 |
| ENSMUSG00000055866 | Per2 | 0.598070013 | 3.385353245 | 0.001057513 |
| ENSMUSG00000021403 | Serpinb9b | -0.740262457 | 6.195032346 | 0.001061489 |
| ENSMUSG00000030409 | Dmpk | 0.377757071 | 7.914067237 | 0.001066948 |
| ENSMUSG00000032702 | Kank1 | 0.34448473 | 4.953223208 | 0.001070487 |
| ENSMUSG00000020340 | Cyfip2 | -0.258276573 | 5.341896391 | 0.001106555 |
| ENSMUSG00000039956 | Mrap | 0.303044024 | 7.748410316 | 0.001122223 |
| ENSMUSG00000031465 | Angpt2 | -0.303101126 | 4.703208403 | 0.001139206 |
| ENSMUSG00000073421 | H2-Ab1 | 0.324552338 | 10.12831788 | 0.00114247 |
| ENSMUSG00000037112 | Sik2 | 0.294357351 | 5.298983804 | 0.001145323 |
| ENSMUSG00000029304 | Spp1 | -0.442168335 | 7.919937856 | 0.001158997 |
| ENSMUSG00000033268 | Duox1 | 0.78567785 | 0.654253627 | 0.001159363 |
| ENSMUSG00000031438 | Rnf128 | -0.363538362 | 6.932639508 | 0.001163555 |
| ENSMUSG00000031958 | Ldhd | 0.338372583 | 4.074705378 | 0.001202234 |
| ENSMUSG00000001025 | S100a6 | -0.244156764 | 9.727310479 | 0.001218014 |
| ENSMUSG00000050556 | Kcnb1 | 0.360231018 | 4.393085821 | 0.001231239 |
| ENSMUSG00000021998 | Lcp1 | -0.167837313 | 10.1093885 | 0.001234179 |
| ENSMUSG00000054555 | Adam12 | -0.293591302 | 5.241826814 | 0.001240614 |
| ENSMUSG00000056888 | Glipr1 | -0.29204823 | 7.091025549 | 0.001249126 |
| ENSMUSG00000067924 | Rtl8b | 0.27820186 | 4.325978701 | 0.001263727 |
| ENSMUSG00000039166 | Akap7 | 0.304667572 | 3.934698956 | 0.001267606 |
| ENSMUSG00000070953 | Rabepk | 0.212367255 | 4.437253349 | 0.001267706 |
| ENSMUSG00000057606 | Colq | 0.522727928 | 1.572721677 | 0.001276426 |
| ENSMUSG00000022623 | Shank3 | 0.377640376 | 5.804417469 | 0.001281702 |
| ENSMUSG00000030513 | Pcsk6 | 0.376862616 | 3.94467882 | 0.001283245 |
| ENSMUSG00000021948 | Prkcd | -0.197077548 | 8.511819433 | 0.001287236 |
| ENSMUSG00000024972 | Lgals12 | 0.364369431 | 5.674173883 | 0.001296961 |
| ENSMUSG00000019779 | Frk | -0.487791203 | 2.069003757 | 0.001316003 |
| ENSMUSG00000025558 | Dock9 | 0.33809161 | 4.941320856 | 0.001317307 |
| ENSMUSG00000028691 | Prdx1 | -0.381717621 | 10.16560033 | 0.001327309 |
| ENSMUSG00000026077 | Npas2 | -0.409857515 | 3.616569311 | 0.001328565 |
| ENSMUSG00000032531 | Amotl2 | 0.235016997 | 6.58145255 | 0.001333148 |
| ENSMUSG00000031785 | Adgrg1 | 0.340810793 | 6.504540436 | 0.001341005 |
| ENSMUSG00000033207 | Mamdc2 | -0.362106006 | 6.350802855 | 0.00134105 |
| ENSMUSG00000027068 | Dhrs9 | -0.326394835 | 4.929022196 | 0.001354401 |
| ENSMUSG00000045287 | Rtn4rl1 | 0.303008811 | 5.434382713 | 0.001354435 |
| ENSMUSG00000036943 | Rab8b | -0.204219537 | 6.95686462 | 0.001370413 |
| ENSMUSG00000022496 | Tnfrsf17 | 0.929877141 | -0.043244763 | 0.001387693 |
| ENSMUSG00000030865 | Chp2 | 0.402310738 | 4.272848409 | 0.001410318 |
| ENSMUSG00000020593 | Lpin1 | 0.25540976 | 6.868137309 | 0.001428102 |
| ENSMUSG00000027848 | Olfml3 | 0.258902052 | 5.475079474 | 0.001453353 |
| ENSMUSG00000051000 | Fam160a1 | 0.474678677 | 2.564030198 | 0.001459461 |
| ENSMUSG00000056054 | S100a8 | 0.556015685 | 3.797724595 | 0.001469639 |
| ENSMUSG00000075254 | Heg1 | 0.277824843 | 6.295601564 | 0.001480038 |
| ENSMUSG00000019558 | Slc6a8 | -0.213623974 | 7.773575482 | 0.001485549 |
| ENSMUSG00000042842 | Serpinb6b | -0.360460467 | 6.075411721 | 0.001498192 |
| ENSMUSG00000030087 | Klf15 | 0.497837359 | 4.099415837 | 0.001513284 |
| ENSMUSG00000042331 | Specc1 | -0.216768668 | 6.038502093 | 0.00152631 |
| ENSMUSG00000016024 | Lbp | 0.269413655 | 8.594685914 | 0.001531447 |
| ENSMUSG00000026628 | Atf3 | -0.253433565 | 8.366243797 | 0.001533012 |
| ENSMUSG00000020399 | Havcr2 | -0.359341896 | 3.705057602 | 0.001534003 |
| ENSMUSG00000029675 | Eln | 0.281629201 | 6.811672897 | 0.001538618 |
| ENSMUSG00000097328 | Tnfsf12 | -0.220163622 | 6.681747906 | 0.001552713 |
| ENSMUSG00000028978 | Nos3 | 0.403653872 | 5.555798231 | 0.001556882 |
| ENSMUSG00000026764 | Kif5c | -0.435031456 | 1.849674552 | 0.0015725 |
| ENSMUSG00000079547 | H2-DMb1 | 0.20363665 | 7.323916033 | 0.001594659 |
| ENSMUSG00000030084 | Plxna1 | -0.17967381 | 7.91079985 | 0.001614426 |
| ENSMUSG00000032013 | Trim29 | -0.327556136 | 4.499899097 | 0.00162599 |
| ENSMUSG00000001518 | Itfg2 | -0.245687799 | 3.622372865 | 0.001626566 |
| ENSMUSG00000051359 | Ncald | 0.413196756 | 2.463212054 | 0.001630839 |
| ENSMUSG00000004655 | Aqp1 | 0.384115993 | 8.732941954 | 0.001640701 |
| ENSMUSG00000000290 | Itgb2 | -0.286584711 | 10.13701055 | 0.001647739 |
| ENSMUSG00000044881 | Coa4 | -0.290229703 | 3.794960075 | 0.001661026 |
| ENSMUSG00000020277 | Pfkl | 0.247782408 | 7.601473053 | 0.00166481 |
| ENSMUSG00000021062 | Rab15 | -0.269746644 | 4.200546879 | 0.001670906 |
| ENSMUSG00000040713 | Creg1 | -0.279023101 | 9.408002013 | 0.001674552 |
| ENSMUSG00000018822 | Sfrp5 | 0.337223763 | 7.600273368 | 0.001722783 |
| ENSMUSG00000044167 | Foxo1 | 0.25444032 | 5.173363265 | 0.001768807 |
| ENSMUSG00000105547 | Iglc3 | 2.197616102 | 0.823453708 | 0.00177297 |
| ENSMUSG00000041577 | Prelp | 0.314999524 | 9.897234502 | 0.001794495 |
| ENSMUSG00000044080 | S100a1 | -0.241609828 | 7.040294685 | 0.001798444 |
| ENSMUSG00000019787 | Trdn | 0.658604678 | 4.126344754 | 0.001836467 |
| ENSMUSG00000032028 | Nxpe2 | 0.996040186 | -0.391171571 | 0.001843257 |
| ENSMUSG00000028238 | Atp6v0d2 | -0.199069332 | 8.736371901 | 0.001844371 |
| ENSMUSG00000028399 | Ptprd | 0.395109612 | 3.452942153 | 0.001846928 |
| ENSMUSG00000001773 | Folh1 | 0.639010517 | 2.712797723 | 0.001864753 |
| ENSMUSG00000115480 | Gm49249 | -0.874787919 | -0.001656891 | 0.001887532 |
| ENSMUSG00000022667 | Cd200r1 | -0.224283288 | 5.691773686 | 0.001888805 |
| ENSMUSG00000040035 | Disp2 | 0.544656768 | 1.542556935 | 0.001903768 |
| ENSMUSG00000070737 | Tmem35b | -0.287686393 | 3.800839492 | 0.001904191 |
| ENSMUSG00000071347 | C1qtnf9 | 0.302807675 | 5.452951149 | 0.001928986 |
| ENSMUSG00000021033 | Gstz1 | 0.338508387 | 8.246939089 | 0.001936243 |
| ENSMUSG00000016942 | Tmprss6 | 1.059822088 | -0.222602943 | 0.001944767 |
| ENSMUSG00000021095 | Gsc | -0.658720475 | 0.484005734 | 0.001948468 |
| ENSMUSG00000040751 | Lat2 | -0.234955585 | 8.177204577 | 0.001965805 |
| ENSMUSG00000017390 | Aldoc | -0.457327166 | 2.069340284 | 0.001976629 |
| ENSMUSG00000046618 | Olfml2a | 0.565318519 | 1.535704482 | 0.001995221 |
| ENSMUSG00000032500 | Dclk3 | 0.6302102 | 2.399798668 | 0.00203106 |
| ENSMUSG00000017774 | Myo1c | 0.152644989 | 8.437966989 | 0.002071376 |
| ENSMUSG00000067599 | Klra7 | 0.988070832 | 0.0765235 | 0.00209012 |
| ENSMUSG00000033032 | Afap1l1 | 0.309222055 | 4.556059586 | 0.002090953 |
| ENSMUSG00000078816 | Prkcg | 0.36993948 | 3.272244938 | 0.002095328 |
| ENSMUSG00000067818 | Myl9 | 0.276634163 | 5.989529909 | 0.002104866 |
| ENSMUSG00000002980 | Bcam | 0.286691792 | 6.091459467 | 0.002117242 |
| ENSMUSG00000021130 | Galnt16 | 0.415260662 | 3.20011613 | 0.002118224 |
| ENSMUSG00000039109 | F13a1 | 0.64424056 | 5.123117391 | 0.002119534 |
| ENSMUSG00000031320 | Rps4x | -0.158840239 | 6.458516925 | 0.002120366 |
| ENSMUSG00000021281 | Tnfaip2 | -0.220679256 | 8.003900621 | 0.002125477 |
| ENSMUSG00000021650 | Ptcd2 | -0.200437629 | 4.951398653 | 0.002132191 |
| ENSMUSG00000060477 | Irak2 | 0.217835727 | 5.827488747 | 0.002137068 |
| ENSMUSG00000032322 | Pstpip1 | -0.332457983 | 6.800727471 | 0.002156813 |
| ENSMUSG00000023921 | Mut | 0.217278688 | 5.333899803 | 0.002166725 |
| ENSMUSG00000034612 | Chst11 | -0.247854821 | 5.386086704 | 0.002212067 |
| ENSMUSG00000097572 | Gm26797 | 0.573548379 | 1.323762031 | 0.002226737 |
| ENSMUSG00000029840 | Mtpn | -0.187010158 | 6.968191524 | 0.002253716 |
| ENSMUSG00000078452 | Raet1d | -0.697334587 | 0.908151581 | 0.002260067 |
| ENSMUSG00000023030 | Slc11a2 | -0.159324571 | 5.773245894 | 0.002291596 |
| ENSMUSG00000057315 | Arhgap24 | 0.249513669 | 4.471088593 | 0.002303041 |
| ENSMUSG00000024302 | Dtna | 0.754571776 | 0.582155971 | 0.002316728 |
| ENSMUSG00000024675 | Ms4a4c | 0.611419806 | 1.132780229 | 0.002317667 |
| ENSMUSG00000025153 | Fasn | 0.312104926 | 9.86945221 | 0.0023392 |
| ENSMUSG00000026202 | Tuba4a | -0.37319812 | 5.142690315 | 0.002340436 |
| ENSMUSG00000026335 | Pam | 0.250807165 | 8.146376688 | 0.002346514 |
| ENSMUSG00000030789 | Itgax | -0.258011136 | 8.805540633 | 0.002349244 |
| ENSMUSG00000036594 | H2-Aa | 0.306986273 | 10.35232942 | 0.002379346 |
| ENSMUSG00000060063 | Alox5ap | -0.275118615 | 8.033530207 | 0.002385636 |
| ENSMUSG00000044641 | Pard6b | -0.437095067 | 1.913714581 | 0.002390691 |
| ENSMUSG00000007041 | Clic1 | -0.185743987 | 9.052113909 | 0.00240049 |
| ENSMUSG00000038065 | Mturn | 0.241098743 | 5.104339628 | 0.002403075 |
| ENSMUSG00000022257 | Laptm4b | 0.277009645 | 5.642633634 | 0.002407635 |
| ENSMUSG00000026639 | Lamb3 | 0.39017923 | 4.099896798 | 0.002427411 |
| ENSMUSG00000034641 | Cd300ld | -0.21038056 | 7.424664096 | 0.002435002 |
| ENSMUSG00000030259 | Rassf8 | -0.25229571 | 6.42128181 | 0.00244688 |
| ENSMUSG00000029648 | Flt1 | 0.256886666 | 5.436383779 | 0.002483306 |
| ENSMUSG00000034796 | Cpne7 | 0.598504879 | 1.566608762 | 0.002489071 |
| ENSMUSG00000042770 | Hebp1 | 0.191553042 | 7.064883812 | 0.002491946 |
| ENSMUSG00000031506 | Ptpn7 | -0.298530234 | 6.338939946 | 0.002498383 |
| ENSMUSG00000029994 | Anxa4 | -0.233635803 | 8.359950622 | 0.0025107 |
| ENSMUSG00000083701 | Gm4912 | 0.694908207 | 0.699088973 | 0.00255292 |
| ENSMUSG00000028076 | Cd1d1 | 0.324711408 | 6.894453261 | 0.002605494 |
| ENSMUSG00000030790 | Adm | 0.363272675 | 3.675561794 | 0.002612894 |
| ENSMUSG00000085295 | 4930430E12Rik | -0.34834257 | 2.681400108 | 0.002614235 |
| ENSMUSG00000078485 | Plekhn1 | -0.169437076 | 6.832595141 | 0.002625636 |
| ENSMUSG00000021996 | Esd | -0.295781754 | 8.264628766 | 0.002638434 |
| ENSMUSG00000015947 | Fcgr1 | 0.244201723 | 6.719878133 | 0.002644374 |
| ENSMUSG00000029095 | Ablim2 | 0.317113015 | 3.820637889 | 0.002660939 |
| ENSMUSG00000023348 | Trip6 | 0.193058827 | 5.486664108 | 0.002662951 |
| ENSMUSG00000041609 | Bicdl1 | 0.271114291 | 4.502473666 | 0.002668963 |
| ENSMUSG00000031986 | Sprtn | 0.211358903 | 4.811577676 | 0.002669423 |
| ENSMUSG00000054252 | Fgfr3 | 0.704383228 | 1.979836681 | 0.002670834 |
| ENSMUSG00000028369 | Svep1 | 0.221124003 | 7.513152365 | 0.002684362 |
| ENSMUSG00000020661 | Dnmt3a | -0.219715957 | 7.342496875 | 0.002704318 |
| ENSMUSG00000022295 | Atp6v1c1 | -0.172650477 | 7.353405691 | 0.002768079 |
| ENSMUSG00000037411 | Serpine1 | 0.429057078 | 7.874221599 | 0.00278079 |
| ENSMUSG00000103965 | Gm30173 | 0.658445298 | 0.984371515 | 0.002816644 |
| ENSMUSG00000047992 | Fam69c | -1.017858272 | -0.084003862 | 0.002859666 |
| ENSMUSG00000020262 | Adarb1 | 0.356951817 | 4.464739237 | 0.002861689 |
| ENSMUSG00000022217 | Emc9 | 0.21109304 | 5.42304861 | 0.002863066 |
| ENSMUSG00000020407 | Upp1 | 0.430873539 | 2.128015756 | 0.002866093 |
| ENSMUSG00000049265 | Kcnk3 | 0.353368685 | 5.285007675 | 0.002866191 |
| ENSMUSG00000060586 | H2-Eb1 | 0.327532481 | 10.21621137 | 0.002876063 |
| ENSMUSG00000020108 | Ddit4 | 0.404608024 | 5.761800953 | 0.002882359 |
| ENSMUSG00000001510 | Dlx3 | -0.608101935 | 0.787754044 | 0.002886642 |
| ENSMUSG00000029027 | Dffb | 0.380266448 | 2.504412336 | 0.002910864 |
| ENSMUSG00000019278 | Dpep1 | 0.327504525 | 6.816328363 | 0.002931401 |
| ENSMUSG00000046032 | Snx12 | -0.177399914 | 5.855203991 | 0.002994098 |
| ENSMUSG00000021038 | Vipas39 | -0.183009964 | 6.681038292 | 0.00299849 |
| ENSMUSG00000003477 | Inmt | 0.839261876 | 3.308614608 | 0.003029112 |
| ENSMUSG00000034957 | Cebpa | 0.199592302 | 8.595389506 | 0.003039293 |
| ENSMUSG00000005103 | Wdr1 | -0.156784193 | 8.993212339 | 0.003061653 |
| ENSMUSG00000031673 | Cdh11 | -0.314883157 | 3.867356452 | 0.00309561 |
| ENSMUSG00000026975 | Dph7 | -0.229265184 | 3.721860872 | 0.003101329 |
| ENSMUSG00000024042 | Sik1 | 0.27768545 | 5.695091521 | 0.003113799 |
| ENSMUSG00000033096 | Apmap | 0.199883458 | 6.955186732 | 0.00312151 |
| ENSMUSG00000018845 | Unc45b | 0.300737229 | 4.151685315 | 0.003128214 |
| ENSMUSG00000033220 | Rac2 | -0.286839693 | 8.146970604 | 0.003146213 |
| ENSMUSG00000039196 | Orm1 | 0.333231526 | 6.558784457 | 0.003173776 |
| ENSMUSG00000038903 | Ccdc68 | 0.954808871 | 0.280653347 | 0.003201833 |
| ENSMUSG00000009941 | Nxf2 | 0.830876079 | -0.046094327 | 0.003204884 |
| ENSMUSG00000097715 | Gpr137b-ps | -0.23893148 | 5.74260093 | 0.003221437 |
| ENSMUSG00000027253 | Lrp4 | -0.301158236 | 2.940520234 | 0.003229694 |
| ENSMUSG00000007107 | Atp1a4 | 0.392520747 | 3.46505224 | 0.003256935 |
| ENSMUSG00000029710 | Ephb4 | 0.300941475 | 5.735235669 | 0.003268521 |
| ENSMUSG00000112148 | Lilrb4a | -0.263284806 | 5.883909862 | 0.003269488 |
| ENSMUSG00000015337 | Endog | 0.267198647 | 3.648055623 | 0.003275415 |
| ENSMUSG00000020627 | Klhl29 | 0.4518193 | 2.023007948 | 0.003319307 |
| ENSMUSG00000054263 | Lifr | 0.24961556 | 5.088804244 | 0.003320618 |
| ENSMUSG00000051984 | Sec31b | 0.429128882 | 2.157463724 | 0.00332691 |
| ENSMUSG00000037071 | Scd1 | 0.392316469 | 11.58481253 | 0.003327046 |
| ENSMUSG00000006611 | Hfe | 0.184312261 | 7.140358486 | 0.003382162 |
| ENSMUSG00000024899 | Papss2 | 0.268281389 | 4.228211739 | 0.003401172 |
| ENSMUSG00000020092 | Pald1 | 0.294579791 | 4.521368754 | 0.003440332 |
| ENSMUSG00000009185 | Ccl8 | 0.579532607 | 5.32917082 | 0.003490528 |
| ENSMUSG00000030220 | Arhgdib | -0.226252341 | 8.519284538 | 0.003498579 |
| ENSMUSG00000043488 | Frmd8os | 0.819686628 | -0.047348893 | 0.003544605 |
| ENSMUSG00000055044 | Pdlim1 | 0.289907136 | 5.022683809 | 0.003559261 |
| ENSMUSG00000097061 | 9330151L19Rik | -0.30654707 | 2.780445747 | 0.003564969 |
| ENSMUSG00000024990 | Rbp4 | 0.375765971 | 6.459567535 | 0.00361492 |
| ENSMUSG00000085178 | Kdm6bos | 0.984236747 | -0.491294262 | 0.003633544 |
| ENSMUSG00000055322 | Tns1 | 0.262178022 | 9.50397263 | 0.003644066 |
| ENSMUSG00000022546 | Gpt | 0.306677076 | 6.848385189 | 0.003650927 |
| ENSMUSG00000044122 | Proca1 | 0.311864364 | 3.601629651 | 0.003669232 |
| ENSMUSG00000089872 | Rps6kc1 | -0.173005583 | 5.502948286 | 0.003682265 |
| ENSMUSG00000042978 | Sbk1 | 0.230457131 | 5.059364861 | 0.003685576 |
| ENSMUSG00000030393 | Zik1 | 0.819796326 | -0.199880106 | 0.003701003 |
| ENSMUSG00000047712 | Ust | 0.302076589 | 3.550978025 | 0.003718129 |
| ENSMUSG00000025161 | Slc16a3 | -0.403239695 | 4.05143498 | 0.003736979 |
| ENSMUSG00000035109 | Shc4 | -0.389484612 | 1.804818162 | 0.003747987 |
| ENSMUSG00000025270 | Alas2 | 0.667588491 | 3.512944537 | 0.003748732 |
| ENSMUSG00000021238 | Aldh6a1 | 0.296459421 | 5.92703172 | 0.003768048 |
| ENSMUSG00000031886 | Ces2e | 0.626391611 | 1.190439146 | 0.003772269 |
| ENSMUSG00000073418 | C4b | 0.323268733 | 9.469685744 | 0.003774928 |
| ENSMUSG00000062609 | Kcnj15 | 0.307909223 | 5.419581103 | 0.003777005 |
| ENSMUSG00000024529 | Lox | 0.370187886 | 6.01320276 | 0.003806665 |
| ENSMUSG00000029223 | Uchl1 | -0.545654371 | 6.06009926 | 0.00382021 |
| ENSMUSG00000054793 | Cadm4 | -0.434184003 | 4.320057358 | 0.003824945 |
| ENSMUSG00000032502 | Stac | 0.600752165 | 1.346568898 | 0.003828003 |
| ENSMUSG00000028088 | Fmo5 | 0.200247098 | 4.280448344 | 0.003857945 |
| ENSMUSG00000003032 | Klf4 | 0.258915174 | 5.357210412 | 0.003858283 |
| ENSMUSG00000039474 | Wfs1 | -0.237760707 | 8.357137844 | 0.003861751 |
| ENSMUSG00000026796 | Fam129b | -0.201771774 | 9.793516154 | 0.003871649 |
| ENSMUSG00000020788 | Atp2a3 | 0.307542898 | 5.874799719 | 0.003872379 |
| ENSMUSG00000036181 | Hist1h1c | -0.217707368 | 7.537799218 | 0.003880232 |
| ENSMUSG00000008090 | Fgfrl1 | 0.280611837 | 7.088236753 | 0.003900554 |
| ENSMUSG00000021313 | Ryr2 | 0.509783876 | 1.545319678 | 0.003922723 |
| ENSMUSG00000044734 | Serpinb1a | -0.403668817 | 3.92614615 | 0.003933827 |
| ENSMUSG00000026023 | Cdk15 | -0.285205581 | 3.195667391 | 0.003941136 |
| ENSMUSG00000053279 | Aldh1a1 | 0.305183148 | 6.331314951 | 0.003941671 |
| ENSMUSG00000047446 | Arl4a | 0.213292407 | 5.326828354 | 0.003957888 |
| ENSMUSG00000062908 | Acadm | 0.176892946 | 7.858704881 | 0.003976913 |
| ENSMUSG00000058486 | Wdr91 | -0.176457387 | 7.165166669 | 0.003979543 |
| ENSMUSG00000049670 | Morn4 | 0.769652665 | 0.180871867 | 0.003980901 |
| ENSMUSG00000038708 | Golga4 | -0.179024842 | 6.135018589 | 0.003985964 |
| ENSMUSG00000078566 | Bnip3 | 0.284074095 | 5.17403937 | 0.003989108 |
| ENSMUSG00000061410 | Zcchc14 | 0.190430845 | 4.982359638 | 0.003989561 |
| ENSMUSG00000028780 | Sema3c | 0.270755518 | 4.547777666 | 0.003996087 |
| ENSMUSG00000030786 | Itgam | -0.254897904 | 7.043373686 | 0.004004677 |
| ENSMUSG00000034781 | Gna11 | 0.165300849 | 6.532696555 | 0.004009173 |
| ENSMUSG00000021091 | Serpina3n | 0.336513998 | 9.137320158 | 0.00401198 |
| ENSMUSG00000036395 | Glb1l2 | 0.316579146 | 6.547599022 | 0.004014062 |
| ENSMUSG00000049878 | Rlf | 0.322280659 | 4.392052571 | 0.004035505 |
| ENSMUSG00000046719 | Nxph3 | -0.310956657 | 3.670002719 | 0.004060197 |
| ENSMUSG00000037003 | Tns2 | 0.307855248 | 7.337328753 | 0.004074771 |
| ENSMUSG00000045106 | Ccdc73 | 0.680054959 | 0.353782845 | 0.004080219 |
| ENSMUSG00000058603 | Rpl28-ps1 | -1.034938819 | -0.570982751 | 0.004091992 |
| ENSMUSG00000015647 | Lama5 | 0.341943381 | 6.1244674 | 0.004109401 |
| ENSMUSG00000016194 | Hsd11b1 | 0.399818363 | 5.798902559 | 0.004116068 |
| ENSMUSG00000031380 | Vegfd | 0.244152676 | 5.202608613 | 0.004155013 |
| ENSMUSG00000017446 | C1qtnf1 | 0.272237412 | 5.779981382 | 0.004184558 |
| ENSMUSG00000043822 | Adamtsl5 | -0.257576006 | 6.062510013 | 0.004236184 |
| ENSMUSG00000022579 | Gpihbp1 | 0.318734105 | 6.673051959 | 0.004239226 |
| ENSMUSG00000046280 | She | 0.310415688 | 3.20950291 | 0.004261627 |
| ENSMUSG00000055254 | Ntrk2 | 0.348861062 | 5.681039782 | 0.004263732 |
| ENSMUSG00000079168 | Cd209g | 1.051235089 | -0.529571957 | 0.004282232 |
| ENSMUSG00000054932 | Afp | -0.535013773 | 3.07815552 | 0.004289118 |
| ENSMUSG00000054383 | Pnma1 | 0.966572314 | -0.460671928 | 0.004299272 |
| ENSMUSG00000006273 | Atp6v1b2 | -0.16068211 | 9.551074795 | 0.004303518 |
| ENSMUSG00000019996 | Map7 | 0.334031412 | 3.61386829 | 0.00431546 |
| ENSMUSG00000030278 | Cidec | 0.277000849 | 10.79379555 | 0.004323213 |
| ENSMUSG00000042284 | Itga1 | 0.310907042 | 5.187884803 | 0.004335817 |
| ENSMUSG00000061086 | Myl4 | 0.328458783 | 3.928331682 | 0.004392954 |
| ENSMUSG00000029153 | Ociad2 | -0.321161743 | 3.643917551 | 0.004398628 |
| ENSMUSG00000073940 | Hbb-bt | 0.614311519 | 5.242773608 | 0.004400274 |
| ENSMUSG00000028124 | Gclm | -0.293265261 | 6.587054096 | 0.004404283 |
| ENSMUSG00000052305 | Hbb-bs | 0.531553141 | 7.710932471 | 0.004421922 |
| ENSMUSG00000019590 | Cyb561 | 0.313197677 | 3.397895892 | 0.004429796 |
| ENSMUSG00000020901 | Pik3r5 | -0.168183256 | 7.383755716 | 0.004444297 |
| ENSMUSG00000018171 | Vmp1 | -0.193001691 | 7.023419818 | 0.004451287 |
| ENSMUSG00000021037 | Ahsa1 | -0.163106557 | 7.024738134 | 0.0044679 |
| ENSMUSG00000028645 | Slc2a1 | -0.232293923 | 5.673168561 | 0.004503504 |
| ENSMUSG00000046417 | Lrrc75a | -0.288216256 | 3.33346828 | 0.004516641 |
| ENSMUSG00000033361 | Prrg3 | 0.271194555 | 4.81183947 | 0.004530712 |
| ENSMUSG00000038370 | Pcp4l1 | -0.343798195 | 5.76943111 | 0.004533611 |
| ENSMUSG00000025348 | Itga7 | 0.273530718 | 8.821392055 | 0.004556104 |
| ENSMUSG00000031253 | Srpx2 | 0.205660108 | 5.874302645 | 0.004559119 |
| ENSMUSG00000024664 | Fads3 | 0.264297236 | 8.869282151 | 0.004571632 |
| ENSMUSG00000074813 | Gm14005 | -0.410459523 | 2.519859603 | 0.004620676 |
| ENSMUSG00000000278 | Scpep1 | -0.175805653 | 8.350952251 | 0.004643289 |
| ENSMUSG00000022636 | Alcam | -0.226609059 | 7.013692578 | 0.004643319 |
| ENSMUSG00000070720 | Tmem200b | 0.418352825 | 1.969835122 | 0.004654705 |
| ENSMUSG00000048498 | Cd300e | -0.654410394 | 0.278182516 | 0.00467252 |
| ENSMUSG00000085024 | C230035I16Rik | -0.684102021 | 0.093325427 | 0.004679872 |
| ENSMUSG00000024737 | Slc15a3 | -0.164353422 | 7.60539934 | 0.004689096 |
| ENSMUSG00000021750 | Fam107a | 0.869871544 | 1.41739889 | 0.004690242 |
| ENSMUSG00000044258 | Ctla2a | 0.234151211 | 4.530590856 | 0.004739715 |
| ENSMUSG00000053398 | Phgdh | 0.332981878 | 5.145757133 | 0.004753357 |
| ENSMUSG00000024975 | Pdcd4 | 0.170914449 | 5.182881272 | 0.004764572 |
| ENSMUSG00000040212 | Emp3 | -0.239559536 | 8.723090368 | 0.004765522 |
| ENSMUSG00000071713 | Csf2rb | -0.169073616 | 6.965861985 | 0.004771261 |
| ENSMUSG00000025534 | Gusb | -0.167726019 | 9.35298415 | 0.004778437 |
| ENSMUSG00000097574 | C920006O11Rik | 0.449179311 | 2.012764269 | 0.004787251 |
| ENSMUSG00000040043 | Rbms2 | 0.204685784 | 5.879081254 | 0.004830318 |
| ENSMUSG00000046598 | Bdh1 | 0.86940401 | -0.397726824 | 0.004852567 |
| ENSMUSG00000084124 | Gm13588 | -0.891652464 | -0.419801304 | 0.004854308 |
| ENSMUSG00000061132 | Blnk | -0.172643256 | 7.728708548 | 0.004855698 |
| ENSMUSG00000020044 | Timp3 | 0.358330029 | 6.827447402 | 0.004865376 |
| ENSMUSG00000054428 | Atpif1 | -0.196933428 | 7.730557477 | 0.004868595 |
| ENSMUSG00000043017 | Ptgir | -0.203016575 | 4.758559848 | 0.004872538 |
| ENSMUSG00000029321 | Slc10a6 | 0.486201655 | 2.659633913 | 0.004882446 |
| ENSMUSG00000020250 | Txnrd1 | -0.217123031 | 7.808545308 | 0.00491228 |
| ENSMUSG00000078651 | Aoc2 | 0.430391353 | 1.993683239 | 0.004920884 |
| ENSMUSG00000025324 | Atp10a | 0.328345696 | 4.138007808 | 0.004936685 |
| ENSMUSG00000025092 | Hspa12a | 0.331163966 | 5.259171951 | 0.004941727 |
| ENSMUSG00000098178 | Gm42418 | -0.647261145 | 9.017407157 | 0.004942556 |
| ENSMUSG00000035227 | Spcs2 | -0.140836756 | 6.383919905 | 0.004948014 |
| ENSMUSG00000058396 | Gpr182 | 0.352815795 | 3.278320567 | 0.00497747 |
| ENSMUSG00000053062 | Jam2 | 0.212383789 | 4.796894402 | 0.004983283 |
| ENSMUSG00000047407 | Tgif1 | -0.199909857 | 5.783622587 | 0.005034025 |
| ENSMUSG00000056899 | Immp2l | 0.37035769 | 2.452359458 | 0.005051165 |
| ENSMUSG00000047497 | Adamts12 | -0.257148315 | 4.738850128 | 0.005075471 |
| ENSMUSG00000038677 | Scube3 | 0.506105375 | 1.217664126 | 0.005082271 |
| ENSMUSG00000019088 | Dnase1l1 | -0.174971955 | 7.475582599 | 0.005087083 |
| ENSMUSG00000059049 | Frem1 | -0.543985227 | 2.101017432 | 0.005090941 |
| ENSMUSG00000085211 | B430219N15Rik | 0.518435493 | 2.45464532 | 0.005097211 |
| ENSMUSG00000036585 | Fgf1 | 0.262825109 | 6.354773599 | 0.005137947 |
| ENSMUSG00000063873 | Slc24a3 | 0.23038777 | 5.906014515 | 0.005142125 |
| ENSMUSG00000024063 | Lbh | 0.153547635 | 6.681276787 | 0.005150215 |
| ENSMUSG00000031740 | Mmp2 | -0.216728386 | 8.838200889 | 0.005156693 |
| ENSMUSG00000029552 | Tes | -0.201467673 | 5.713495431 | 0.005163405 |
| ENSMUSG00000025262 | Fam120c | 0.621764766 | 0.540441208 | 0.005189115 |
| ENSMUSG00000032462 | Pik3cb | -0.182353927 | 5.506916441 | 0.005203738 |
| ENSMUSG00000061288 | Taok3 | -0.194066961 | 6.701604213 | 0.005204272 |
| ENSMUSG00000039103 | Nexn | 0.452895633 | 2.836699871 | 0.005216688 |
| ENSMUSG00000045095 | Magi1 | 0.331803797 | 4.570869902 | 0.005275114 |
| ENSMUSG00000001053 | N4bp3 | 0.331399911 | 3.964661554 | 0.005292912 |
| ENSMUSG00000029553 | Tfec | -0.290600394 | 3.772263888 | 0.005302666 |
| ENSMUSG00000033365 | Ipo13 | -0.159635362 | 6.433997229 | 0.005309616 |
| ENSMUSG00000040652 | Oaz2 | 0.192675875 | 5.014734077 | 0.005316211 |
| ENSMUSG00000020154 | Ptprb | 0.286378424 | 5.668555208 | 0.005322358 |
| ENSMUSG00000008730 | Hipk1 | 0.179437928 | 6.082894836 | 0.005361895 |
| ENSMUSG00000090291 | Lrrc10b | -0.557361156 | 1.028328493 | 0.005381393 |
| ENSMUSG00000096401 | Gm21811 | 0.718974613 | 0.161664072 | 0.0054635 |
| ENSMUSG00000046070 | Igfals | 0.393971696 | 5.951867645 | 0.00548321 |
| ENSMUSG00000030144 | Clec4d | -0.397351387 | 7.165286279 | 0.005491828 |
| ENSMUSG00000028980 | H6pd | 0.185252649 | 8.193616504 | 0.005531129 |
| ENSMUSG00000037736 | Limch1 | 0.363882682 | 4.711928603 | 0.005550726 |
| ENSMUSG00000087107 | AI662270 | -0.213836596 | 4.429604176 | 0.005560816 |
| ENSMUSG00000017897 | Eya2 | 0.242831854 | 4.895806334 | 0.005585921 |
| ENSMUSG00000033453 | Adamts15 | -0.286886829 | 5.593669787 | 0.005606305 |
| ENSMUSG00000071708 | Sms | -0.297191051 | 3.04528253 | 0.005667215 |
| ENSMUSG00000031762 | Mt2 | 0.299132008 | 7.953471722 | 0.005707385 |
| ENSMUSG00000025085 | Ablim1 | 0.180454043 | 6.290998129 | 0.005732266 |
| ENSMUSG00000032714 | Syde1 | 0.249048822 | 5.626264009 | 0.0057611 |
| ENSMUSG00000041737 | Tmem45b | 0.395427923 | 7.066463029 | 0.005772956 |
| ENSMUSG00000038816 | Ctnnal1 | 0.354190454 | 3.454630973 | 0.005787758 |
| ENSMUSG00000033209 | Ttc28 | 0.275064529 | 5.611177014 | 0.005802357 |
| ENSMUSG00000063558 | Aox1 | 0.24412622 | 6.046722662 | 0.005813681 |
| ENSMUSG00000000881 | Dlg3 | -0.251222025 | 5.479814888 | 0.005860432 |
| ENSMUSG00000000223 | Drp2 | 0.636432553 | 0.819694574 | 0.005881309 |
| ENSMUSG00000048486 | Fitm2 | 0.233012618 | 6.450945317 | 0.005906276 |
| ENSMUSG00000027999 | Pla2g12a | 0.232215505 | 5.565798655 | 0.005945541 |
| ENSMUSG00000027692 | Tnik | 0.500873763 | 1.136943128 | 0.005972416 |
| ENSMUSG00000053581 | Zfand2a | -0.163985486 | 6.628245537 | 0.005972659 |
| ENSMUSG00000039628 | Hs3st6 | 0.757324641 | 0.063632513 | 0.005975371 |
| ENSMUSG00000048497 | Mmgt2 | -0.242584287 | 3.863354453 | 0.005979361 |
| ENSMUSG00000033192 | Lpcat2 | -0.325325923 | 4.084609422 | 0.005982513 |
| ENSMUSG00000004558 | Ndrg2 | 0.32737534 | 6.854892351 | 0.005984976 |
| ENSMUSG00000049598 | Vsig8 | -0.421157785 | 6.830964592 | 0.005988236 |
| ENSMUSG00000015143 | Actn1 | -0.131909956 | 8.210962514 | 0.005989948 |
| ENSMUSG00000034853 | Acot11 | -0.218022325 | 4.226104781 | 0.00603004 |
| ENSMUSG00000062515 | Fabp4 | 0.234117692 | 12.74924191 | 0.006035949 |
| ENSMUSG00000039943 | Plcb4 | 0.304674345 | 3.88900621 | 0.006049724 |
| ENSMUSG00000034006 | Pqlc1 | -0.198797063 | 6.246964851 | 0.0060849 |
| ENSMUSG00000022367 | Has2 | -0.80464765 | 0.36536091 | 0.006114016 |
| ENSMUSG00000021868 | Ppif | 0.201607837 | 4.080132435 | 0.006134399 |
| ENSMUSG00000017631 | Abr | -0.173690614 | 6.632290071 | 0.006137973 |
| ENSMUSG00000025815 | Dhtkd1 | 0.502227283 | 1.320267072 | 0.006172222 |
| ENSMUSG00000037111 | Setd7 | 0.210812613 | 5.541935233 | 0.00622904 |
| ENSMUSG00000023809 | Rps6ka2 | -0.337584371 | 3.781712862 | 0.006245395 |
| ENSMUSG00000035085 | 1700020L24Rik | 0.682593253 | 0.375899039 | 0.006246121 |
| ENSMUSG00000036856 | Wnt4 | -0.730578342 | 0.499409088 | 0.006265304 |
| ENSMUSG00000033379 | Atp6v0b | -0.172571024 | 8.335708084 | 0.006272559 |
| ENSMUSG00000071715 | Ncf4 | -0.206991369 | 6.729063589 | 0.006273073 |
| ENSMUSG00000032085 | Tagln | 0.274409065 | 6.557655158 | 0.006285112 |
| ENSMUSG00000039713 | Plekhg5 | 0.218845866 | 5.557358416 | 0.006296614 |
| ENSMUSG00000024300 | Myo1f | -0.196879807 | 8.941576665 | 0.006347301 |
| ENSMUSG00000035929 | H2-Q4 | 0.235446832 | 7.063740863 | 0.006361036 |
| ENSMUSG00000006219 | Fblim1 | -0.160981939 | 7.352798809 | 0.006388497 |
| ENSMUSG00000029851 | Tcaf2 | 0.311248297 | 3.118266306 | 0.006391233 |
| ENSMUSG00000051748 | Wfdc21 | 0.334830619 | 5.378714967 | 0.006398921 |
| ENSMUSG00000043740 | B430306N03Rik | -0.311259113 | 5.864350702 | 0.006444784 |
| ENSMUSG00000034560 | Washc4 | -0.182176356 | 4.530864938 | 0.006464115 |
| ENSMUSG00000051682 | Treml4 | -0.411422169 | 2.098402334 | 0.006473752 |
| ENSMUSG00000034930 | Rtkn | -0.254974921 | 3.682007939 | 0.006483501 |
| ENSMUSG00000035168 | Tanc1 | 0.245636022 | 4.750515424 | 0.006495857 |
| ENSMUSG00000112023 | Lilr4b | -0.21495213 | 8.771627291 | 0.006510259 |
| ENSMUSG00000022032 | Scara5 | 0.414984459 | 5.030216708 | 0.006565132 |
| ENSMUSG00000025150 | Cbr2 | 0.399642536 | 4.699419747 | 0.006568184 |
| ENSMUSG00000050896 | Rtn4rl2 | 0.342369791 | 3.429575055 | 0.00659415 |
| ENSMUSG00000050578 | Mmp13 | -0.432016194 | 2.928553706 | 0.006624984 |
| ENSMUSG00000023048 | Prr13 | -0.25905656 | 8.3409878 | 0.006638759 |
| ENSMUSG00000055805 | Fmnl1 | -0.152297645 | 6.949996287 | 0.006664154 |
| ENSMUSG00000044854 | 1700056E22Rik | 0.459763204 | 1.856278955 | 0.006730684 |
| ENSMUSG00000034652 | Cd300a | -0.230468416 | 8.152626873 | 0.006746352 |
| ENSMUSG00000026204 | Ptprn | -0.413608325 | 2.709640522 | 0.006779602 |
| ENSMUSG00000041229 | Phf8 | 0.196676735 | 5.248758638 | 0.006780677 |
| ENSMUSG00000027499 | Pkia | 0.442618172 | 3.195160378 | 0.006783657 |
| ENSMUSG00000035783 | Acta2 | 0.309920772 | 7.825671692 | 0.006787714 |
| ENSMUSG00000031963 | Bmper | -0.244434628 | 5.764027934 | 0.006806069 |
| ENSMUSG00000055745 | Rtl6 | 0.275102968 | 2.971174192 | 0.0068865 |
| ENSMUSG00000034684 | Sema3f | 0.317185316 | 5.218296164 | 0.006914135 |
| ENSMUSG00000091243 | Vgll3 | 0.250114036 | 5.398788725 | 0.00691784 |
| ENSMUSG00000056737 | Capg | -0.240069019 | 10.38203496 | 0.006947999 |
| ENSMUSG00000028656 | Cap1 | -0.145225112 | 9.261799816 | 0.007013536 |
| ENSMUSG00000021846 | Peli2 | 0.23258108 | 4.137195333 | 0.007015057 |
| ENSMUSG00000036880 | Acaa2 | 0.149851568 | 7.600350437 | 0.007020336 |
| ENSMUSG00000038618 | Rassf7 | 0.355339453 | 2.057107147 | 0.007048961 |
| ENSMUSG00000051043 | Gprc5c | -0.185198996 | 5.414379047 | 0.007049941 |
| ENSMUSG00000037852 | Cpe | -0.320434512 | 8.042401757 | 0.007072112 |
| ENSMUSG00000017943 | Gdap1l1 | -0.724387762 | 0.198182461 | 0.007110039 |
| ENSMUSG00000026259 | Ngef | -0.473745203 | 1.905760315 | 0.007138359 |
| ENSMUSG00000022351 | Sqle | -0.208297561 | 5.123871762 | 0.007141861 |
| ENSMUSG00000097462 | 9530026P05Rik | 0.78935733 | 0.208116903 | 0.007180036 |
| ENSMUSG00000023088 | Abcc1 | -0.175316861 | 6.730317735 | 0.007219877 |
| ENSMUSG00000026395 | Ptprc | -0.180647303 | 7.353144522 | 0.007230684 |
| ENSMUSG00000023050 | Map3k12 | -0.209065274 | 4.3489768 | 0.007234174 |
| ENSMUSG00000030427 | Lilra6 | -0.51113483 | 1.246283473 | 0.007242411 |
| ENSMUSG00000107726 | Gm44037 | -0.679124757 | 0.107512248 | 0.007293098 |
| ENSMUSG00000037661 | Gpr160 | 0.291762114 | 3.701711285 | 0.007320897 |
| ENSMUSG00000001983 | Taco1 | 0.282972349 | 3.176557615 | 0.007342056 |
| ENSMUSG00000000266 | Mid2 | 0.343848366 | 3.141110029 | 0.007350122 |
| ENSMUSG00000027656 | Ccn5 | 0.372569199 | 6.368440758 | 0.007363065 |
| ENSMUSG00000015224 | Cyp2j9 | 0.343335192 | 2.744830214 | 0.007385592 |
| ENSMUSG00000038145 | Snrk | 0.245266701 | 5.768712959 | 0.007445021 |
| ENSMUSG00000001020 | S100a4 | -0.324076984 | 6.752512503 | 0.007511821 |
| ENSMUSG00000033022 | Cdo1 | 0.344464503 | 8.478774962 | 0.007529385 |
| ENSMUSG00000032802 | Srxn1 | -0.260927506 | 8.202576845 | 0.007539448 |
| ENSMUSG00000027187 | Cat | -0.161795354 | 9.283400031 | 0.007541799 |
| ENSMUSG00000032011 | Thy1 | -0.291253255 | 6.732526887 | 0.007545613 |
| ENSMUSG00000041930 | Fam222a | 0.474314642 | 2.201829363 | 0.007555893 |
| ENSMUSG00000009687 | Fxyd5 | -0.177890706 | 8.476327377 | 0.007564646 |
| ENSMUSG00000029406 | Pitpnm2 | 0.230506863 | 5.576936197 | 0.007565218 |
| ENSMUSG00000030546 | Plin1 | 0.274170295 | 9.551068657 | 0.007612152 |
| ENSMUSG00000023074 | Mospd1 | 0.240979686 | 3.583138538 | 0.007614892 |
| ENSMUSG00000026883 | Dab2ip | 0.247641806 | 6.198938533 | 0.007690705 |
| ENSMUSG00000029765 | Plxna4 | -0.236711408 | 4.737627149 | 0.007717635 |
| ENSMUSG00000020032 | Nuak1 | 0.216780107 | 4.245688126 | 0.007737453 |
| ENSMUSG00000045038 | Prkce | 0.284199424 | 3.81638386 | 0.007772919 |
| ENSMUSG00000037217 | Syn1 | -0.30588926 | 3.679168924 | 0.007780298 |
| ENSMUSG00000019437 | Tlcd1 | 0.290526646 | 4.499589323 | 0.007868193 |
| ENSMUSG00000022469 | Rapgef3 | 0.330268593 | 5.637472318 | 0.00787212 |
| ENSMUSG00000100658 | F730311O21Rik | -0.505753281 | 1.373673676 | 0.007874665 |
| ENSMUSG00000021253 | Tgfb3 | 0.223947369 | 4.940137568 | 0.007880275 |
| ENSMUSG00000113757 | Gm47507 | -0.627834157 | 1.786610135 | 0.007924961 |
| ENSMUSG00000072082 | Ccnf | -0.232702095 | 4.068572339 | 0.007953254 |
| ENSMUSG00000101438 | Gm19412 | 0.379852728 | 1.920108464 | 0.007964703 |
| ENSMUSG00000034037 | Fgd5 | 0.301337284 | 4.465763129 | 0.007996591 |
| ENSMUSG00000002059 | Rab34 | 0.175732577 | 6.073238175 | 0.008007632 |
| ENSMUSG00000052560 | Cpne8 | 0.278635936 | 3.778802323 | 0.008032088 |
| ENSMUSG00000054342 | Kcnn4 | -0.238629476 | 7.499359221 | 0.008046178 |
| ENSMUSG00000040152 | Thbs1 | 0.365047101 | 7.141719397 | 0.008051696 |
| ENSMUSG00000002578 | Ikzf4 | 0.86240392 | -0.401024427 | 0.008057043 |
| ENSMUSG00000042429 | Adora1 | 0.33948262 | 6.202591128 | 0.00809784 |
| ENSMUSG00000022265 | Ank | -0.162069287 | 6.429093826 | 0.008105171 |
| ENSMUSG00000002831 | Plin4 | 0.355653755 | 9.673943568 | 0.008125921 |
| ENSMUSG00000037868 | Egr2 | -0.284150129 | 6.22337088 | 0.00816348 |
| ENSMUSG00000049130 | C5ar1 | -0.162314338 | 8.188594222 | 0.008213797 |
| ENSMUSG00000036555 | Iqce | -0.172247241 | 4.782965097 | 0.008226642 |
| ENSMUSG00000051515 | Fam181b | 0.544854889 | 1.156931689 | 0.008273405 |
| ENSMUSG00000031748 | Gnao1 | 0.364860081 | 4.135684558 | 0.008294095 |
| ENSMUSG00000053964 | Lgals4 | 0.192302435 | 4.362367815 | 0.008416545 |
| ENSMUSG00000006589 | Aprt | -0.215781361 | 7.958812617 | 0.00843101 |
| ENSMUSG00000040006 | Ginm1 | 0.184267728 | 6.589335877 | 0.008431705 |
| ENSMUSG00000023861 | Mpc1 | 0.275852888 | 3.686912847 | 0.008456519 |
| ENSMUSG00000048537 | Phldb1 | 0.190528556 | 7.390234418 | 0.008461467 |
| ENSMUSG00000057440 | Mpp7 | 0.291698754 | 3.546930043 | 0.008463648 |
| ENSMUSG00000071540 | 3425401B19Rik | 0.756641501 | 0.601756146 | 0.008469927 |
| ENSMUSG00000020251 | Glt8d2 | 0.34981917 | 1.812391357 | 0.008481073 |
| ENSMUSG00000084512 | Gm22482 | 0.70765712 | 0.176683913 | 0.00851874 |
| ENSMUSG00000003617 | Cp | 0.250452932 | 5.546877012 | 0.008533538 |
| ENSMUSG00000025880 | Smad7 | 0.310405786 | 3.41355749 | 0.008558231 |
| ENSMUSG00000021360 | Gcnt2 | 0.241144416 | 5.64534928 | 0.008589713 |
| ENSMUSG00000019326 | Aoc3 | 0.2593476 | 9.028239174 | 0.008598293 |
| ENSMUSG00000109603 | Gm32389 | 0.536230987 | 0.918696366 | 0.008619177 |
| ENSMUSG00000026701 | Prdx6 | 0.180810099 | 8.490171519 | 0.00862184 |
| ENSMUSG00000019986 | Ahi1 | -0.399596333 | 2.166507579 | 0.008624519 |
| ENSMUSG00000028692 | Akr1a1 | -0.164971737 | 10.70424481 | 0.008640595 |
| ENSMUSG00000022206 | Npr3 | 0.266498596 | 8.5280367 | 0.008656667 |
| ENSMUSG00000027634 | Ndrg3 | -0.138663313 | 5.907963971 | 0.008746833 |
| ENSMUSG00000040147 | Maob | 0.27532343 | 3.749179277 | 0.008748692 |
| ENSMUSG00000025351 | Cd63 | -0.192526453 | 8.858683227 | 0.008810783 |
| ENSMUSG00000026834 | Acvr1c | 0.329799092 | 3.200474356 | 0.008821077 |
| ENSMUSG00000052698 | Tln2 | 0.314289816 | 6.475322316 | 0.008851549 |
| ENSMUSG00000076441 | Ass1 | 0.493633753 | 3.398515489 | 0.008869138 |
| ENSMUSG00000020674 | Pxdn | 0.268630825 | 6.600737499 | 0.008893694 |
| ENSMUSG00000075590 | Nrbp2 | 0.244247887 | 5.068482621 | 0.0088942 |
| ENSMUSG00000059182 | Skap2 | -0.152386586 | 7.125036572 | 0.008917009 |
| ENSMUSG00000087382 | Ctcflos | 0.30536717 | 5.096220729 | 0.008924622 |
| ENSMUSG00000049791 | Fzd4 | 0.263849159 | 7.482415 | 0.008995727 |
| ENSMUSG00000029326 | Enoph1 | -0.159711889 | 4.682019896 | 0.009047583 |
| ENSMUSG00000018566 | Slc2a4 | 0.353465699 | 5.377148981 | 0.009062569 |
| ENSMUSG00000028776 | Tinagl1 | 0.253295764 | 7.261740554 | 0.009073862 |
| ENSMUSG00000025203 | Scd2 | 0.29217449 | 8.0564499 | 0.009129063 |
| ENSMUSG00000090150 | Acad11 | 0.203787765 | 5.848507384 | 0.009155291 |
| ENSMUSG00000021994 | Wnt5a | 0.283070268 | 3.539303483 | 0.009157047 |
| ENSMUSG00000027224 | Duoxa1 | 0.248797948 | 4.119420308 | 0.009162872 |
| ENSMUSG00000038563 | Efl1 | -0.164460911 | 4.44102479 | 0.009194931 |
| ENSMUSG00000020495 | Smg8 | -0.189734856 | 4.239367204 | 0.009195922 |
| ENSMUSG00000038375 | Trp53inp2 | 0.209495351 | 9.393853822 | 0.009217608 |
| ENSMUSG00000053040 | Aph1c | -0.204256318 | 5.019216367 | 0.009232199 |
| ENSMUSG00000044340 | Phlpp1 | 0.187768931 | 3.989506138 | 0.009243882 |
| ENSMUSG00000039646 | Vasn | 0.225133838 | 5.206649843 | 0.009274196 |
| ENSMUSG00000020592 | Sdc1 | -0.255592978 | 7.160957837 | 0.009290622 |
| ENSMUSG00000048572 | Tmem252 | 0.393795825 | 4.067290416 | 0.009305293 |
| ENSMUSG00000039518 | Cdsn | 0.384726236 | 5.905303198 | 0.009361243 |
| ENSMUSG00000035279 | Ssc5d | -0.219366674 | 6.2624737 | 0.009432907 |
| ENSMUSG00000024897 | Apba1 | 0.200973211 | 3.876481199 | 0.009475174 |
| ENSMUSG00000045333 | Zfp423 | 0.280868317 | 5.052389512 | 0.009477032 |
| ENSMUSG00000028367 | Txn1 | -0.21806577 | 8.636950061 | 0.009491066 |
| ENSMUSG00000110613 | Lncbate1 | 0.556974463 | 0.826126368 | 0.009502854 |
| ENSMUSG00000037060 | Cavin3 | 0.218792709 | 8.278358451 | 0.009527757 |
| ENSMUSG00000044452 | Zfp507 | 0.301138407 | 2.547135998 | 0.009532113 |
| ENSMUSG00000060860 | Ube2s | -0.205373336 | 3.672848232 | 0.009552385 |
| ENSMUSG00000000686 | Abhd15 | 0.244630575 | 4.456016676 | 0.009569498 |
| ENSMUSG00000033386 | Frrs1 | -0.175961843 | 5.905981004 | 0.009594723 |
| ENSMUSG00000015202 | Cnksr3 | 0.211539056 | 4.08487376 | 0.009599063 |
| ENSMUSG00000023143 | Nagpa | -0.179424054 | 6.362944988 | 0.009600978 |
| ENSMUSG00000071176 | Arhgef10 | 0.204667692 | 4.783137069 | 0.009648625 |
| ENSMUSG00000107838 | Gm45769 | 0.438932106 | 1.132304557 | 0.009701061 |
| ENSMUSG00000043939 | A530064D06Rik | -0.425335918 | 3.821083378 | 0.009722526 |
| ENSMUSG00000074793 | Hspa12b | 0.274162822 | 5.033987599 | 0.009729341 |
| ENSMUSG00000001436 | Slc19a1 | 0.201193225 | 4.437247294 | 0.0097436 |
| ENSMUSG00000027994 | Mcub | -0.197015328 | 5.66448257 | 0.009790041 |
| ENSMUSG00000020375 | Rufy1 | -0.149043275 | 6.024106034 | 0.009806953 |
| ENSMUSG00000044927 | H1fx | 0.455625108 | 1.980732046 | 0.00981159 |
| ENSMUSG00000034509 | Mad2l1bp | -0.208323282 | 4.410639774 | 0.009815852 |
| ENSMUSG00000055737 | Ghr | 0.263913663 | 8.152292766 | 0.009830695 |
| ENSMUSG00000046415 | B430212C06Rik | 0.349786726 | 2.761715599 | 0.009843087 |
| ENSMUSG00000000326 | Comt | 0.174120469 | 8.745569158 | 0.009893471 |
| ENSMUSG00000056602 | Fry | 0.265686107 | 5.553749313 | 0.009917538 |
| ENSMUSG00000070348 | Ccnd1 | -0.197991181 | 7.09611234 | 0.009932492 |
| ENSMUSG00000030413 | Pglyrp1 | 0.445109689 | 1.276864854 | 0.009994489 |
| ENSMUSG00000074264 | Amy1 | 0.416759524 | 4.953789616 | 0.010018318 |
| ENSMUSG00000112794 | Gm48878 | -0.723515616 | 0.009532818 | 0.010038389 |
| ENSMUSG00000003123 | Lipe | 0.293402259 | 9.788966596 | 0.010060175 |
| ENSMUSG00000026228 | Htr2b | -0.360962326 | 3.524119666 | 0.010109042 |
| ENSMUSG00000039131 | Gipc2 | -0.553015273 | 0.880265978 | 0.010113003 |
| ENSMUSG00000027611 | Procr | -0.322731925 | 5.688584192 | 0.010165324 |
| ENSMUSG00000002346 | Slc25a42 | 0.286602579 | 4.129003427 | 0.010180725 |
| ENSMUSG00000069919 | Hba-a1 | 0.531981874 | 5.951844682 | 0.010244017 |
| ENSMUSG00000035936 | Aldh5a1 | 0.263940521 | 3.306820714 | 0.010270991 |
| ENSMUSG00000038188 | Scarf1 | 0.207601651 | 5.08629285 | 0.010276085 |
| ENSMUSG00000025776 | Crispld1 | -0.785066512 | -0.362930959 | 0.010282113 |
| ENSMUSG00000007891 | Ctsd | -0.189555936 | 12.82764326 | 0.010327525 |
| ENSMUSG00000033170 | Card10 | 0.248223839 | 5.457216294 | 0.010376614 |
| ENSMUSG00000031838 | Ifi30 | -0.213591013 | 8.89550115 | 0.010392852 |
| ENSMUSG00000069917 | Hba-a2 | 0.515013926 | 5.210784952 | 0.010399857 |
| ENSMUSG00000038007 | Acer2 | 0.377241879 | 4.143705686 | 0.010405021 |
| ENSMUSG00000036086 | Zranb3 | -0.33275593 | 6.449468925 | 0.010422288 |
| ENSMUSG00000023913 | Pla2g7 | -0.301476176 | 8.050505618 | 0.010428382 |
| ENSMUSG00000023830 | Igf2r | -0.142121169 | 8.078171499 | 0.010429037 |
| ENSMUSG00000049624 | Slc17a5 | -0.166770372 | 5.284816862 | 0.010489878 |
| ENSMUSG00000025911 | Adhfe1 | 0.352818878 | 5.728403696 | 0.010519527 |
| ENSMUSG00000051652 | Lrrc3 | 0.365333176 | 3.102347898 | 0.010527376 |
| ENSMUSG00000060147 | Serpinb6a | -0.163874915 | 9.480923693 | 0.01053161 |
| ENSMUSG00000024617 | Camk2a | -0.214926309 | 4.06066016 | 0.010600284 |
| ENSMUSG00000028159 | Dapp1 | -0.220080547 | 4.448162225 | 0.010648999 |
| ENSMUSG00000050587 | Lrrc4c | 0.477627849 | 1.47922273 | 0.010654594 |
| ENSMUSG00000029695 | Aass | 0.821630642 | -0.075352418 | 0.0106607 |
| ENSMUSG00000024132 | Eci1 | 0.183366446 | 5.87198255 | 0.010697349 |
| ENSMUSG00000020424 | Castor1 | 0.306854259 | 4.366749766 | 0.010700778 |
| ENSMUSG00000032204 | Aqp9 | 0.498465829 | 0.914442558 | 0.010710497 |
| ENSMUSG00000027207 | Galk2 | -0.174283373 | 5.745260848 | 0.010714274 |
| ENSMUSG00000022021 | Diaph3 | -0.313408348 | 2.647670775 | 0.010730196 |
| ENSMUSG00000030747 | Dgat2 | 0.265416559 | 10.72549474 | 0.010766103 |
| ENSMUSG00000071662 | Polr2g | -0.14200771 | 5.938324638 | 0.010767807 |
| ENSMUSG00000070493 | Chchd2 | 0.154079575 | 6.391254105 | 0.010770044 |
| ENSMUSG00000032271 | Nnmt | 0.221204079 | 7.349628802 | 0.010786977 |
| ENSMUSG00000021245 | Mlh3 | -0.247244882 | 3.28813513 | 0.010804201 |
| ENSMUSG00000027799 | Nbea | 0.336407971 | 2.155326702 | 0.010821823 |
| ENSMUSG00000032382 | Snx1 | -0.162308307 | 7.45347843 | 0.010846149 |
| ENSMUSG00000056069 | Otulinl | -0.171563276 | 5.945924515 | 0.011055297 |
| ENSMUSG00000009614 | Sardh | 0.194556117 | 5.827186613 | 0.011063773 |
| ENSMUSG00000053749 | Gm9920 | 0.534734386 | 0.800969616 | 0.01106801 |
| ENSMUSG00000034295 | Fhod3 | 0.404106053 | 2.20293332 | 0.011093098 |
| ENSMUSG00000022504 | Ciita | 0.236027174 | 4.939481728 | 0.011096275 |
| ENSMUSG00000020681 | Ace | 0.237776609 | 6.574606931 | 0.011153753 |
| ENSMUSG00000033308 | Dpyd | 0.454498572 | 2.279542385 | 0.011162593 |
| ENSMUSG00000068079 | Tcf15 | 0.41727888 | 2.954907607 | 0.011166477 |
| ENSMUSG00000023886 | Smoc2 | 0.227268019 | 6.868308821 | 0.011178976 |
| ENSMUSG00000039126 | Prune2 | -0.198599834 | 5.203112582 | 0.011216963 |
| ENSMUSG00000045629 | Sh3tc2 | 0.382297604 | 3.092711548 | 0.011232351 |
| ENSMUSG00000039431 | Mtmr7 | -0.56855951 | 0.40366349 | 0.011278556 |
| ENSMUSG00000024065 | Ehd3 | 0.281452187 | 4.838195258 | 0.011279084 |
| ENSMUSG00000024039 | Cbs | 0.675843814 | 1.059452903 | 0.01130184 |
| ENSMUSG00000020882 | Cacnb1 | -0.301187952 | 2.551166634 | 0.011317107 |
| ENSMUSG00000025969 | Nrp2 | -0.144908592 | 7.245605412 | 0.011324725 |
| ENSMUSG00000023961 | Enpp4 | 0.267786989 | 3.426280999 | 0.01135458 |
| ENSMUSG00000031387 | Renbp | -0.170108304 | 7.189026902 | 0.011355049 |
| ENSMUSG00000029059 | Fam213b | 0.217138557 | 6.687299424 | 0.011429248 |
| ENSMUSG00000030433 | Sbk2 | -0.271586371 | 3.607269022 | 0.011439499 |
| ENSMUSG00000046223 | Plaur | -0.220906149 | 6.435736179 | 0.011440909 |
| ENSMUSG00000051236 | Msrb3 | 0.201641048 | 5.505395211 | 0.011461533 |
| ENSMUSG00000032788 | Pdxk | -0.166389592 | 7.890981357 | 0.011477575 |
| ENSMUSG00000022698 | Naa50 | -0.167254349 | 6.186734927 | 0.011484619 |
| ENSMUSG00000043004 | Gng2 | -0.16732368 | 6.471462909 | 0.01150108 |
| ENSMUSG00000018965 | Ywhah | -0.153872786 | 6.670467591 | 0.011640926 |
| ENSMUSG00000023411 | Nfatc4 | -0.191046342 | 4.212399087 | 0.011746268 |
| ENSMUSG00000035277 | Arx | 0.362226857 | 3.477873363 | 0.011799702 |
| ENSMUSG00000033632 | AW554918 | 0.261190921 | 2.854290004 | 0.011827197 |
| ENSMUSG00000004951 | Hspb1 | 0.3108971 | 4.671291351 | 0.011827658 |
| ENSMUSG00000020357 | Flt4 | 0.29828387 | 3.31487311 | 0.011828819 |
| ENSMUSG00000070047 | Fat1 | 0.18113109 | 5.391851512 | 0.011844481 |
| ENSMUSG00000034854 | Mfsd12 | -0.187913739 | 8.883449023 | 0.011844797 |
| ENSMUSG00000036446 | Lum | -0.251806814 | 7.867090424 | 0.011850569 |
| ENSMUSG00000022488 | Nckap1l | -0.139694507 | 8.869545936 | 0.011858822 |
| ENSMUSG00000025939 | Ube2w | 0.219874439 | 4.613305408 | 0.011902607 |
| ENSMUSG00000021294 | Kif26a | 0.293055587 | 4.049756422 | 0.011917266 |
| ENSMUSG00000039137 | Whrn | 0.414983463 | 1.44987194 | 0.011998018 |
| ENSMUSG00000041654 | Slc39a11 | -0.178040265 | 6.415845955 | 0.012010057 |
| ENSMUSG00000022831 | Hcls1 | -0.166396989 | 8.495067739 | 0.012046789 |
| ENSMUSG00000004207 | Psap | -0.165374064 | 13.75770326 | 0.012065938 |
| ENSMUSG00000000386 | Mx1 | 0.4564857 | 3.422085735 | 0.012075401 |
| ENSMUSG00000022178 | Ajuba | 0.215518663 | 4.27776666 | 0.012198951 |
| ENSMUSG00000028444 | Cntfr | 0.526308821 | 2.691870919 | 0.012204522 |
| ENSMUSG00000069806 | Cacng7 | 0.227490932 | 4.298138117 | 0.012207197 |
| ENSMUSG00000097103 | Gm2885 | -0.734148526 | -0.3146365 | 0.012226003 |
| ENSMUSG00000083443 | Gm15519 | 0.557039668 | 1.294926948 | 0.012263238 |
| ENSMUSG00000057530 | Ece1 | 0.214456584 | 7.412873576 | 0.012276247 |
| ENSMUSG00000045180 | Shroom2 | 0.497280214 | 2.582814176 | 0.012303394 |
| ENSMUSG00000097440 | Gm6277 | 0.373089911 | 3.704422804 | 0.012314642 |
| ENSMUSG00000041895 | Wipi1 | 0.161829047 | 5.719629569 | 0.012374588 |
| ENSMUSG00000052151 | Plpp2 | -0.176863054 | 4.851633391 | 0.012378019 |
| ENSMUSG00000055782 | Abcd2 | 0.230136314 | 6.862459577 | 0.012396463 |
| ENSMUSG00000029833 | Trim24 | 0.196918067 | 4.883834599 | 0.012401101 |
| ENSMUSG00000032192 | Gnb5 | 0.192315011 | 4.765495808 | 0.01241977 |
| ENSMUSG00000050335 | Lgals3 | -0.197277929 | 12.49656602 | 0.012433591 |
| ENSMUSG00000075225 | Ccdc162 | 0.685515294 | -0.101420607 | 0.012456798 |
| ENSMUSG00000028845 | Tekt2 | -0.395652793 | 1.467429556 | 0.012474591 |
| ENSMUSG00000066510 | Ankdd1a | -0.28495148 | 3.736499441 | 0.01251017 |
| ENSMUSG00000026404 | Ddx59 | 0.27139914 | 3.138821562 | 0.01253444 |
| ENSMUSG00000031434 | Morc4 | 0.260541294 | 3.798141206 | 0.012592109 |
| ENSMUSG00000058153 | Sez6l | 0.53004714 | 2.036639572 | 0.012592935 |
| ENSMUSG00000020541 | Tom1l1 | 0.415748984 | 2.347763658 | 0.012599634 |
| ENSMUSG00000032280 | Tle3 | 0.141627409 | 6.619166224 | 0.012619639 |
| ENSMUSG00000032436 | Cmtm7 | -0.171514823 | 6.225496104 | 0.012620563 |
| ENSMUSG00000031995 | St14 | 0.31245082 | 3.292333703 | 0.012643284 |
| ENSMUSG00000020019 | Ntn4 | 0.252967442 | 3.423312692 | 0.012679201 |
| ENSMUSG00000049313 | Sorl1 | 0.182008228 | 4.637672959 | 0.012699423 |
| ENSMUSG00000025265 | Fgd1 | -0.278757141 | 2.552727999 | 0.012702245 |
| ENSMUSG00000030579 | Tyrobp | -0.172829902 | 10.19213539 | 0.012705279 |
| ENSMUSG00000037251 | Pomk | -0.147481548 | 4.672954587 | 0.012750585 |
| ENSMUSG00000028957 | Per3 | 0.401063651 | 3.875982239 | 0.012756081 |
| ENSMUSG00000020686 | Gas2l2 | 0.559845484 | 1.223612799 | 0.012759506 |
| ENSMUSG00000072769 | Gm10419 | -0.502498447 | 1.352421005 | 0.012774077 |
| ENSMUSG00000026490 | Cdc42bpa | 0.201085505 | 5.170233075 | 0.012780351 |
| ENSMUSG00000000915 | Hip1r | 0.322826019 | 3.973966101 | 0.012964194 |
| ENSMUSG00000046733 | Gprc5a | 0.499841261 | 0.855190405 | 0.012984789 |
| ENSMUSG00000031520 | Vegfc | 0.380830992 | 3.494336706 | 0.012993655 |
| ENSMUSG00000039512 | Uhrf1bp1 | 0.201494137 | 4.740636432 | 0.013021879 |
| ENSMUSG00000049871 | Nlrc3 | -0.252346478 | 3.475288524 | 0.013039076 |
| ENSMUSG00000050721 | Plekho2 | -0.145865162 | 9.050565937 | 0.013130492 |
| ENSMUSG00000042660 | Wdr55 | -0.163441229 | 4.581964263 | 0.013145462 |
| ENSMUSG00000053799 | Exoc6 | 0.167022669 | 4.645935175 | 0.013239449 |
| ENSMUSG00000024601 | Isoc1 | 0.160224762 | 5.499561206 | 0.013260156 |
| ENSMUSG00000042589 | Cux2 | 0.576932154 | 2.016669417 | 0.01329546 |
| ENSMUSG00000101089 | 2610016A17Rik | 0.396606532 | 3.498655823 | 0.013326414 |
| ENSMUSG00000019978 | Epb41l2 | 0.171569826 | 8.281238025 | 0.013331596 |
| ENSMUSG00000114019 | Gm47155 | 0.697311591 | -0.09079474 | 0.01333735 |
| ENSMUSG00000030674 | Qprt | -0.29242541 | 3.083695702 | 0.013355895 |
| ENSMUSG00000076617 | Ighm | 0.275750017 | 9.342280046 | 0.013367709 |
| ENSMUSG00000001520 | Nrip2 | 0.498409512 | 2.900999474 | 0.013378897 |
| ENSMUSG00000020085 | Aifm2 | 0.237798697 | 7.726933154 | 0.01345392 |
| ENSMUSG00000050199 | Lgr4 | 0.338346179 | 4.060265982 | 0.013455701 |
| ENSMUSG00000026921 | Egfl7 | 0.245176705 | 6.825092635 | 0.013569437 |
| ENSMUSG00000026825 | Dnm1 | -0.215816801 | 5.661845441 | 0.013596942 |
| ENSMUSG00000027750 | Postn | -0.373721938 | 9.299653185 | 0.013648268 |
| ENSMUSG00000021186 | Fbln5 | 0.211961841 | 6.135227198 | 0.013664096 |
| ENSMUSG00000022428 | Cby1 | -0.179122524 | 4.375844794 | 0.013690622 |
| ENSMUSG00000061232 | H2-K1 | 0.161269046 | 10.92893634 | 0.013698424 |
| ENSMUSG00000009112 | Bcl2l13 | 0.189092399 | 6.278477286 | 0.013716455 |
| ENSMUSG00000053007 | Creb5 | 0.310312271 | 2.786220765 | 0.013807638 |
| ENSMUSG00000032673 | Prorsd1 | -0.20126589 | 3.837528401 | 0.013853668 |
| ENSMUSG00000001123 | Lgals9 | 0.164468431 | 7.191152608 | 0.013865715 |
| ENSMUSG00000056758 | Hmga2 | -0.438053864 | 3.488377881 | 0.013896037 |
| ENSMUSG00000031144 | Syp | 0.281335583 | 5.782365024 | 0.013950621 |
| ENSMUSG00000020684 | Rasl10b | 0.676501219 | 0.156320551 | 0.013966542 |
| ENSMUSG00000083460 | Gm12182 | 0.754803585 | -0.269676328 | 0.013994328 |
| ENSMUSG00000017760 | Ctsa | -0.16684991 | 10.37749931 | 0.014000261 |
| ENSMUSG00000024781 | Lipa | -0.176157623 | 10.21519519 | 0.014032757 |
| ENSMUSG00000038319 | Kcnh2 | 0.423122939 | 3.973477985 | 0.014035385 |
| ENSMUSG00000097334 | 2700012I20Rik | -0.844759983 | -0.39417341 | 0.014108919 |
| ENSMUSG00000024118 | Tedc2 | -0.241715837 | 3.570934671 | 0.014110749 |
| ENSMUSG00000018774 | Cd68 | -0.183445686 | 10.79911477 | 0.014137536 |
| ENSMUSG00000015829 | Tnr | 0.731647563 | 0.451143388 | 0.014179106 |
| ENSMUSG00000050708 | Ftl1 | -0.254465712 | 10.76731527 | 0.014192666 |
| ENSMUSG00000028024 | Enpep | 0.281214214 | 5.732748569 | 0.014210958 |
| ENSMUSG00000086938 | 4930481A15Rik | -0.316916264 | 2.580875227 | 0.01423518 |
| ENSMUSG00000024924 | Vldlr | 0.259155404 | 6.019648834 | 0.014237129 |
| ENSMUSG00000004267 | Eno2 | -0.182154829 | 5.26123889 | 0.01427318 |
| ENSMUSG00000026874 | Hc | -0.792044532 | 1.675808558 | 0.014372677 |
| ENSMUSG00000027792 | Bche | 0.404511766 | 3.555342884 | 0.014467846 |
| ENSMUSG00000026123 | Plekhb2 | -0.155732131 | 7.02013597 | 0.014485561 |
| ENSMUSG00000036533 | Cdc42ep3 | 0.240189821 | 4.08589352 | 0.014495386 |
| ENSMUSG00000070495 | Ctcfl | 0.653315621 | -0.016671522 | 0.014550217 |
| ENSMUSG00000089901 | Gm8113 | -0.482708558 | 1.695358233 | 0.014558012 |
| ENSMUSG00000025650 | Col7a1 | -0.327128914 | 2.344003488 | 0.014637416 |
| ENSMUSG00000031563 | Wwc2 | 0.184802208 | 5.032029095 | 0.01464677 |
| ENSMUSG00000022096 | Hr | 0.320829273 | 5.373211882 | 0.014695468 |
| ENSMUSG00000020846 | Rflnb | 0.296322637 | 5.569502356 | 0.014747779 |
| ENSMUSG00000028427 | Aqp7 | 0.260453827 | 5.70927969 | 0.014762298 |
| ENSMUSG00000050910 | Cdr2l | -0.181800223 | 4.579008221 | 0.014778928 |
| ENSMUSG00000042742 | Bmt2 | -0.294135313 | 3.774991524 | 0.014805246 |
| ENSMUSG00000054418 | 2900041M22Rik | 0.535956999 | 0.375319063 | 0.014812351 |
| ENSMUSG00000027490 | E2f1 | -0.180478469 | 5.619464585 | 0.01483049 |
| ENSMUSG00000049960 | Mrps16 | -0.154331262 | 5.020323468 | 0.014844008 |
| ENSMUSG00000021684 | Pde8b | -0.316885053 | 3.691331489 | 0.014908168 |
| ENSMUSG00000078630 | Tomt | 0.567260736 | 0.3767924 | 0.014914674 |
| ENSMUSG00000063415 | Cyp26b1 | 0.575318199 | 4.069146081 | 0.014935555 |
| ENSMUSG00000031431 | Tsc22d3 | 0.271256798 | 7.262127216 | 0.014950795 |
| ENSMUSG00000004707 | Ly9 | -0.242622359 | 7.165024016 | 0.015010944 |
| ENSMUSG00000022604 | Cep97 | -0.359722935 | 2.013657993 | 0.015026267 |
| ENSMUSG00000071068 | Treml2 | 0.653831852 | 0.188752568 | 0.015031595 |
| ENSMUSG00000027175 | Tcp11l1 | -0.242666082 | 2.815947151 | 0.015044619 |
| ENSMUSG00000090207 | 4930524O07Rik | 0.859893694 | -0.679493438 | 0.015049769 |
| ENSMUSG00000027386 | Fbln7 | -0.329091778 | 5.744729724 | 0.015059157 |
| ENSMUSG00000000532 | Acvr1b | 0.145150784 | 5.789218024 | 0.015063512 |
| ENSMUSG00000042401 | Crtac1 | 0.320667785 | 5.671622688 | 0.015074432 |
| ENSMUSG00000040181 | Fmo1 | 0.275203753 | 5.183332207 | 0.015078369 |
| ENSMUSG00000007655 | Cav1 | 0.254488698 | 9.745840621 | 0.015087576 |
| ENSMUSG00000047766 | Lrrc49 | -0.220469564 | 3.527815953 | 0.015112559 |
| ENSMUSG00000031983 | 2310022B05Rik | 0.138275903 | 6.125437065 | 0.015137562 |
| ENSMUSG00000014791 | Elmo3 | 0.265129711 | 3.485758588 | 0.015156862 |
| ENSMUSG00000062132 | Arhgap33os | 0.755163233 | -0.165147811 | 0.015214694 |
| ENSMUSG00000025268 | Maged2 | -0.143803675 | 6.245315617 | 0.015280365 |
| ENSMUSG00000029338 | Antxr2 | -0.149005946 | 6.551034206 | 0.015288549 |
| ENSMUSG00000066571 | 4931406P16Rik | 0.188171135 | 5.059123833 | 0.015326975 |
| ENSMUSG00000033278 | Ptprm | 0.227127421 | 4.720441462 | 0.015346902 |
| ENSMUSG00000086552 | Dlx4os | -0.557384223 | 0.300901836 | 0.015367677 |
| ENSMUSG00000020451 | Limk2 | 0.18351476 | 5.573218433 | 0.015369067 |
| ENSMUSG00000063605 | Ccdc102a | 0.20421387 | 3.929512177 | 0.015405465 |
| ENSMUSG00000033966 | Cdkl4 | 0.382707927 | 4.309495721 | 0.015423351 |
| ENSMUSG00000024014 | Pim1 | -0.162881243 | 5.053443961 | 0.015431519 |
| ENSMUSG00000029229 | Chic2 | -0.170808422 | 4.516908416 | 0.015436923 |
| ENSMUSG00000101655 | 2310040G24Rik | -0.350612078 | 1.639169448 | 0.015465742 |
| ENSMUSG00000022911 | Arl13b | 0.279445648 | 3.105700464 | 0.015490589 |
| ENSMUSG00000032261 | Sh3bgrl2 | -0.170137482 | 4.686608602 | 0.015518536 |
| ENSMUSG00000044037 | Als2cl | 0.216501181 | 4.876892502 | 0.015571356 |
| ENSMUSG00000030306 | Tmtc1 | 0.451521584 | 2.142344329 | 0.015576841 |
| ENSMUSG00000039616 | Mocos | -0.135087876 | 6.453950954 | 0.015584126 |
| ENSMUSG00000045679 | Pqlc3 | -0.13500533 | 6.449030034 | 0.015585236 |
| ENSMUSG00000086546 | Gm13709 | 0.459483626 | 1.527536806 | 0.015597019 |
| ENSMUSG00000050666 | Vstm4 | -0.17072921 | 4.617320045 | 0.015624645 |
| ENSMUSG00000102615 | Gm37844 | 0.584518446 | 0.631930847 | 0.015700383 |
| ENSMUSG00000026005 | Rpe | 0.207555464 | 5.086928912 | 0.015702403 |
| ENSMUSG00000036330 | Slc18a1 | -0.355148257 | 2.574067301 | 0.015724341 |
| ENSMUSG00000032014 | Oaf | -0.165014876 | 6.465092892 | 0.015724557 |
| ENSMUSG00000020605 | Hs1bp3 | -0.185019514 | 5.933410711 | 0.015736221 |
| ENSMUSG00000031734 | Irx3 | 0.470800251 | 1.475483949 | 0.01574773 |
| ENSMUSG00000061666 | Gdpd1 | -0.195229659 | 4.353737581 | 0.015790079 |
| ENSMUSG00000035513 | Ntng2 | 0.284048322 | 3.707235511 | 0.015801422 |
| ENSMUSG00000006356 | Crip2 | 0.204608231 | 6.993900785 | 0.015810934 |
| ENSMUSG00000025362 | Rps26 | -0.149708656 | 7.301371402 | 0.01584045 |
| ENSMUSG00000037362 | Ccn3 | 0.633458976 | 1.29955602 | 0.015841702 |
| ENSMUSG00000020649 | Rrm2 | -0.201370059 | 4.411500413 | 0.015856009 |
| ENSMUSG00000018411 | Mapt | 0.610202439 | 0.054920793 | 0.015867376 |
| ENSMUSG00000009739 | Pou6f1 | 0.149398032 | 5.080414327 | 0.01591658 |
| ENSMUSG00000056492 | Adgrf5 | 0.227118915 | 6.276761832 | 0.01592242 |
| ENSMUSG00000050174 | Nudt6 | 0.220658971 | 3.027217389 | 0.016057883 |
| ENSMUSG00000024667 | Tmem216 | 0.302116247 | 3.994290033 | 0.016133038 |
| ENSMUSG00000006567 | Atp7b | 0.445953164 | 1.460768563 | 0.016182102 |
| ENSMUSG00000033191 | Tie1 | 0.278438643 | 6.280739739 | 0.016184122 |
| ENSMUSG00000015970 | Chdh | 0.308019441 | 2.506135065 | 0.016242034 |
| ENSMUSG00000097357 | Gm16793 | -0.926785791 | -0.451415468 | 0.01631643 |
| ENSMUSG00000026177 | Slc11a1 | -0.188978435 | 9.067795103 | 0.016339582 |
| ENSMUSG00000067786 | Nnat | 0.440223064 | 7.877364475 | 0.01634748 |
| ENSMUSG00000028247 | Coq3 | 0.178436005 | 4.606899725 | 0.016367295 |
| ENSMUSG00000037295 | Ldlrap1 | -0.128238848 | 7.272130195 | 0.016379472 |
| ENSMUSG00000005373 | Mlxipl | 0.33120209 | 5.52186492 | 0.016422656 |
| ENSMUSG00000091639 | Gm3756 | 0.817549099 | -0.567842191 | 0.016446683 |
| ENSMUSG00000068735 | Trp53i11 | 0.269703168 | 6.230421839 | 0.016457527 |
| ENSMUSG00000002307 | Daxx | 0.14651553 | 6.659302144 | 0.016465514 |
| ENSMUSG00000030498 | Gas2 | 0.400262497 | 1.458416898 | 0.016477578 |
| ENSMUSG00000046961 | Gpr156 | 0.557721764 | 1.566363327 | 0.016530043 |
| ENSMUSG00000022123 | Scel | 0.510042479 | 0.983560177 | 0.016565193 |
| ENSMUSG00000026153 | Fam135a | 0.478584885 | 1.82773296 | 0.016598178 |
| ENSMUSG00000021662 | Arhgef28 | 0.267730529 | 4.267519944 | 0.01664329 |
| ENSMUSG00000024456 | Diaph1 | -0.142367534 | 7.944732632 | 0.016652922 |
| ENSMUSG00000020315 | Sptbn1 | 0.194338441 | 9.03141609 | 0.016673929 |
| ENSMUSG00000044349 | Snhg11 | 0.258162211 | 4.135339791 | 0.016684192 |
| ENSMUSG00000034416 | Pkd1l2 | -0.399150522 | 1.55209534 | 0.016708161 |
| ENSMUSG00000024892 | Pcx | 0.23076067 | 9.115124913 | 0.016723746 |
| ENSMUSG00000044098 | Rsbn1 | 0.310165056 | 2.427325208 | 0.016745224 |
| ENSMUSG00000006818 | Sod2 | 0.180531639 | 7.486380146 | 0.016763055 |
| ENSMUSG00000022548 | Apod | 0.3243806 | 4.596720669 | 0.016777097 |
| ENSMUSG00000098142 | Gm7507 | -0.751933933 | -0.472675411 | 0.01677887 |
| ENSMUSG00000026956 | Uap1l1 | -0.171769627 | 7.760610051 | 0.016864322 |
| ENSMUSG00000032263 | Bckdhb | 0.190732424 | 5.214178814 | 0.016930219 |
| ENSMUSG00000020053 | Igf1 | -0.130287462 | 8.084036948 | 0.016930669 |
| ENSMUSG00000004035 | Gstm7 | 0.409869979 | 2.141748888 | 0.016960662 |
| ENSMUSG00000028613 | Lrp8 | -0.390739322 | 2.728883089 | 0.016985398 |
| ENSMUSG00000030878 | Cdr2 | 0.204917586 | 6.114225932 | 0.017016176 |
| ENSMUSG00000025791 | Pgm1 | 0.166796198 | 6.803607721 | 0.017041507 |
| ENSMUSG00000034330 | Plcg2 | -0.170819811 | 5.857425251 | 0.017055508 |
| ENSMUSG00000026480 | Ncf2 | -0.182556598 | 7.093896181 | 0.017067423 |
| ENSMUSG00000027002 | Nckap1 | 0.18884587 | 6.086252676 | 0.017091628 |
| ENSMUSG00000074207 | Adh1 | 0.244284861 | 6.738360059 | 0.017091859 |
| ENSMUSG00000047394 | Odf3b | 0.604952851 | 0.099464972 | 0.017103666 |
| ENSMUSG00000095385 | D630033O11Rik | -0.408661665 | 1.721908063 | 0.017158934 |
| ENSMUSG00000027605 | Acss2 | 0.202708604 | 6.25370591 | 0.017262095 |
| ENSMUSG00000039208 | Metrnl | -0.180997289 | 7.712221341 | 0.017268334 |
| ENSMUSG00000058427 | Cxcl2 | -0.330022658 | 2.839133348 | 0.017274306 |
| ENSMUSG00000075486 | Commd6 | -0.185920493 | 5.219924432 | 0.017274575 |
| ENSMUSG00000072812 | Ahnak2 | -0.17886625 | 7.987391655 | 0.017289393 |
| ENSMUSG00000026245 | Farsb | -0.155740872 | 5.997084047 | 0.017323828 |
| ENSMUSG00000023827 | Agpat4 | -0.195936886 | 5.319487568 | 0.017351533 |
| ENSMUSG00000055555 | 4930502E18Rik | 0.672459061 | 1.112800523 | 0.017387056 |
| ENSMUSG00000036093 | Arl5a | 0.223046056 | 4.238105485 | 0.017399522 |
| ENSMUSG00000038259 | Gdf5 | 0.661911358 | 0.355000925 | 0.017437059 |
| ENSMUSG00000054203 | Ifi205 | 0.382073973 | 3.881875661 | 0.017536825 |
| ENSMUSG00000097131 | D230017M19Rik | 0.665823113 | -0.088057374 | 0.017563964 |
| ENSMUSG00000070576 | Mn1 | 0.27761526 | 2.684423088 | 0.017634443 |
| ENSMUSG00000038400 | Pmepa1 | 0.290905266 | 7.731190958 | 0.017662617 |
| ENSMUSG00000033389 | Arhgap44 | 0.422191916 | 2.241943774 | 0.017669142 |
| ENSMUSG00000027074 | Slc43a3 | 0.258472458 | 6.678234258 | 0.017679113 |
| ENSMUSG00000021090 | Lrrc9 | 0.74120395 | -0.395629525 | 0.017764429 |
| ENSMUSG00000024747 | Aldh1a7 | 0.283077936 | 6.160676713 | 0.017855625 |
| ENSMUSG00000043903 | Zfp469 | -0.341515861 | 2.176745587 | 0.017861198 |
| ENSMUSG00000089706 | B230216N24Rik | 0.641635395 | 0.050738476 | 0.017977992 |
| ENSMUSG00000073079 | Srp54a | 0.595380769 | 0.467367519 | 0.017989647 |
| ENSMUSG00000029189 | Sel1l3 | -0.199358688 | 5.315030991 | 0.017996654 |
| ENSMUSG00000092274 | Neat1 | -0.153438505 | 11.13228859 | 0.018001572 |
| ENSMUSG00000021993 | Mipep | 0.189910298 | 4.202244147 | 0.018097021 |
| ENSMUSG00000033174 | Mgll | 0.19325213 | 9.995765665 | 0.01814822 |
| ENSMUSG00000053318 | Slamf8 | -0.203112097 | 5.342863843 | 0.018190372 |
| ENSMUSG00000027342 | Pcna | -0.146535675 | 6.922399882 | 0.018206946 |
| ENSMUSG00000060613 | Cyp2c70 | 0.772257033 | -0.126213687 | 0.018230677 |
| ENSMUSG00000051225 | Fam83a | 0.24936122 | 4.40274698 | 0.018232682 |
| ENSMUSG00000015467 | Egfl8 | 0.372729756 | 2.217902229 | 0.018272465 |
| ENSMUSG00000057130 | Txnl4a | -0.162172892 | 4.201588074 | 0.018352979 |
| ENSMUSG00000026626 | Ppp2r5a | 0.181180406 | 7.462715323 | 0.018381067 |
| ENSMUSG00000052396 | Mogat2 | 0.317497851 | 4.336798755 | 0.018398758 |
| ENSMUSG00000034902 | Pip5k1c | -0.165965358 | 8.333758674 | 0.018439349 |
| ENSMUSG00000030894 | Tpp1 | -0.130815356 | 8.541100865 | 0.018459554 |
| ENSMUSG00000049303 | Syt12 | 0.321834231 | 3.832236887 | 0.018476238 |
| ENSMUSG00000102211 | Gm37490 | -0.573920131 | 0.225205137 | 0.018488139 |
| ENSMUSG00000030107 | Usp18 | 0.316087205 | 2.959383852 | 0.018508124 |
| ENSMUSG00000025877 | Hk3 | -0.187462097 | 8.186671902 | 0.018518625 |
| ENSMUSG00000001029 | Icam2 | 0.222640199 | 4.451525175 | 0.018591499 |
| ENSMUSG00000028082 | Sh3d19 | -0.163657158 | 4.721441208 | 0.018697015 |
| ENSMUSG00000002111 | Spi1 | -0.191808315 | 8.436785539 | 0.018741491 |
| ENSMUSG00000060002 | Chpt1 | 0.222326963 | 7.47256249 | 0.018745749 |
| ENSMUSG00000054728 | Phactr1 | -0.260104538 | 3.164394847 | 0.018763442 |
| ENSMUSG00000041193 | Pla2g5 | 0.568699261 | 1.060806249 | 0.018790586 |
| ENSMUSG00000107429 | Gm44206 | -0.74637439 | 0.111414403 | 0.018793335 |
| ENSMUSG00000061353 | Cxcl12 | 0.188435133 | 7.281175741 | 0.018828724 |
| ENSMUSG00000035376 | Hacd2 | 0.236373883 | 6.58190066 | 0.018981745 |
| ENSMUSG00000034664 | Itga2b | -0.491849487 | 2.770219973 | 0.018984583 |
| ENSMUSG00000017400 | Stac2 | -0.293778691 | 6.078025457 | 0.019044269 |
| ENSMUSG00000031264 | Btk | -0.143092508 | 6.045931664 | 0.01904989 |
| ENSMUSG00000030047 | Arhgap25 | -0.206496234 | 8.453943089 | 0.019073536 |
| ENSMUSG00000039167 | Adgrl4 | 0.200003328 | 4.540666476 | 0.019076926 |
| ENSMUSG00000031558 | Slit2 | -0.340698984 | 3.673321834 | 0.019086945 |
| ENSMUSG00000027513 | Pck1 | 0.463318019 | 7.241080182 | 0.019218279 |
| ENSMUSG00000027806 | Tsc22d2 | 0.183172241 | 5.175865536 | 0.019229616 |
| ENSMUSG00000021069 | Pygl | 0.158140133 | 6.059672471 | 0.019235578 |
| ENSMUSG00000017309 | Cd300lg | 0.258936406 | 6.972110945 | 0.019237773 |
| ENSMUSG00000052102 | Gnpda1 | -0.172550552 | 5.26791634 | 0.019297555 |
| ENSMUSG00000036636 | Clcn7 | -0.160032898 | 7.954584119 | 0.019312556 |
| ENSMUSG00000037095 | Lrg1 | 0.224335959 | 9.444487636 | 0.019358916 |
| ENSMUSG00000004446 | Bid | -0.149872752 | 4.869087572 | 0.019360288 |
| ENSMUSG00000049411 | Tmem241 | -0.248248707 | 3.152294081 | 0.019374004 |
| ENSMUSG00000056130 | Ticam2 | -0.238987239 | 3.335531477 | 0.01938083 |
| ENSMUSG00000038843 | Gcnt1 | -0.223765795 | 5.039360926 | 0.019382554 |
| ENSMUSG00000031586 | Rbpms | 0.205491895 | 5.98573477 | 0.01945139 |
| ENSMUSG00000034471 | Caskin2 | 0.231348137 | 5.563134686 | 0.019466327 |
| ENSMUSG00000028680 | Plk3 | 0.236139751 | 4.075407267 | 0.019474642 |
| ENSMUSG00000024232 | Bambi | -0.316329583 | 2.255078649 | 0.019487223 |
| ENSMUSG00000049521 | Cdc42ep1 | 0.271860266 | 7.051582643 | 0.019526077 |
| ENSMUSG00000030168 | Adipor2 | 0.177919451 | 8.129918964 | 0.019568209 |
| ENSMUSG00000069094 | Pde7a | 0.234255733 | 5.050321112 | 0.019612091 |
| ENSMUSG00000038379 | Ttk | -0.36261404 | 1.860588897 | 0.019664406 |
| ENSMUSG00000030536 | Iqgap1 | -0.123753745 | 9.100595909 | 0.019667085 |
| ENSMUSG00000024219 | Anks1 | 0.127994981 | 6.276795646 | 0.019750282 |
| ENSMUSG00000031871 | Cdh5 | 0.26022693 | 7.435581938 | 0.019768773 |
| ENSMUSG00000027890 | Gstm4 | 0.164049767 | 4.612249515 | 0.019777047 |
| ENSMUSG00000037896 | Rcor1 | -0.183054534 | 4.665355156 | 0.019788066 |
| ENSMUSG00000091199 | Gm2619 | 0.7128587 | 0.319881079 | 0.01980342 |
| ENSMUSG00000027335 | Adra1d | 0.355996861 | 3.506278137 | 0.019825234 |
| ENSMUSG00000021699 | Pde4d | 0.32294238 | 2.156109143 | 0.019862571 |
| ENSMUSG00000031289 | Il13ra2 | 0.531652807 | 2.142544503 | 0.019872471 |
| ENSMUSG00000045838 | Ccdc9b | 0.216380634 | 5.404901225 | 0.019945236 |
| ENSMUSG00000037960 | Card19 | -0.226446753 | 7.367305942 | 0.019971253 |
| ENSMUSG00000024403 | Atp6v1g2 | -0.246018173 | 3.107446747 | 0.020051208 |
| ENSMUSG00000032135 | Mcam | 0.252674255 | 8.222787777 | 0.020068335 |
| ENSMUSG00000034858 | Fam214a | 0.182584335 | 5.427536993 | 0.020069274 |
| ENSMUSG00000049404 | Rarres1 | 0.626455345 | 0.241185598 | 0.020082209 |
| ENSMUSG00000046275 | Trarg1 | 0.237627555 | 8.116406531 | 0.020182936 |
| ENSMUSG00000083649 | Rasl2-9 | 0.64374196 | 0.259621631 | 0.020188706 |
| ENSMUSG00000051648 | Kctd19 | 0.624303827 | -0.029316795 | 0.02019277 |
| ENSMUSG00000032661 | Oas3 | 0.438216541 | 2.678423896 | 0.020217022 |
| ENSMUSG00000059555 | Tor4a | -0.179398963 | 6.077192324 | 0.020304622 |
| ENSMUSG00000052459 | Atp6v1a | -0.190441104 | 7.885370577 | 0.020311892 |
| ENSMUSG00000085327 | Gm16104 | -0.432202025 | 1.061264724 | 0.020347011 |
| ENSMUSG00000026678 | Rgs5 | 0.308705041 | 6.660702912 | 0.020379389 |
| ENSMUSG00000090386 | Mir99ahg | -0.321620441 | 1.895740703 | 0.020526024 |
| ENSMUSG00000026360 | Rgs2 | -0.192740815 | 6.703207732 | 0.020568508 |
| ENSMUSG00000036097 | Slf2 | -0.171509463 | 4.202581246 | 0.020621046 |
| ENSMUSG00000045763 | Basp1 | -0.242806932 | 6.640685584 | 0.020623573 |
| ENSMUSG00000038150 | Ormdl3 | 0.189809622 | 7.576687645 | 0.020643672 |
| ENSMUSG00000042622 | Maff | 0.233168698 | 4.344241362 | 0.02064806 |
| ENSMUSG00000029843 | Slc13a4 | 0.328243758 | 3.703922402 | 0.020663855 |
| ENSMUSG00000062309 | Rpp25 | -0.446203913 | 1.053473446 | 0.020727938 |
| ENSMUSG00000022906 | Parp9 | 0.15852778 | 5.951196537 | 0.020798017 |
| ENSMUSG00000002997 | Prkar2b | 0.207346323 | 7.45885673 | 0.021004839 |
| ENSMUSG00000098242 | Gm26929 | -0.643129907 | -0.106911508 | 0.021078756 |
| ENSMUSG00000043122 | A530016L24Rik | 0.335385765 | 5.947550182 | 0.02108953 |
| ENSMUSG00000046826 | Fam187b | 0.690188535 | -0.147587695 | 0.021167744 |
| ENSMUSG00000022197 | Pdzd2 | 0.274580008 | 5.028632872 | 0.021171537 |
| ENSMUSG00000084932 | Gm15156 | -0.341172808 | 2.411504909 | 0.021219857 |
| ENSMUSG00000057948 | Unc13d | -0.206376193 | 4.842327034 | 0.021246964 |
| ENSMUSG00000015134 | Aldh1a3 | 0.402760267 | 4.179624311 | 0.021258577 |
| ENSMUSG00000031934 | Panx1 | -0.221222195 | 5.237734232 | 0.021279467 |
| ENSMUSG00000070643 | Sox13 | 0.269798945 | 4.11029371 | 0.021315013 |
| ENSMUSG00000039347 | Atp6v0e2 | 0.286237469 | 4.324292079 | 0.021356391 |
| ENSMUSG00000029919 | Hpgds | -0.147386923 | 7.338934837 | 0.021361355 |
| ENSMUSG00000018740 | Slc25a35 | 0.28166174 | 3.825000048 | 0.021383406 |
| ENSMUSG00000046352 | Gjb2 | 0.639415975 | 0.070304391 | 0.021459821 |
| ENSMUSG00000026623 | Lpgat1 | 0.2353252 | 8.518088384 | 0.021483086 |
| ENSMUSG00000031672 | Got2 | -0.147256336 | 5.234892522 | 0.021497278 |
| ENSMUSG00000035342 | Lzts2 | 0.184938357 | 6.131364397 | 0.021505033 |
| ENSMUSG00000019478 | Rab4a | -0.244197602 | 3.775377586 | 0.021559271 |
| ENSMUSG00000031628 | Casp3 | -0.140340563 | 4.990078802 | 0.021584723 |
| ENSMUSG00000035004 | Igsf6 | -0.175640689 | 6.553539144 | 0.021586216 |
| ENSMUSG00000027935 | Rab13 | -0.166584349 | 4.964163006 | 0.021590564 |
| ENSMUSG00000063193 | Cd300lb | -0.180320615 | 7.818375485 | 0.021651944 |
| ENSMUSG00000039774 | Galnt12 | -0.14391148 | 4.910858227 | 0.021660556 |
| ENSMUSG00000102545 | 6430573P05Rik | 0.634585319 | 0.263195873 | 0.021838328 |
| ENSMUSG00000000184 | Ccnd2 | 0.19686385 | 7.60733468 | 0.021856863 |
| ENSMUSG00000039911 | Spsb1 | 0.317946412 | 5.338700543 | 0.021863289 |
| ENSMUSG00000029020 | Mfn2 | 0.149113585 | 6.963887372 | 0.021880142 |
| ENSMUSG00000031226 | Pbdc1 | -0.18529773 | 5.023289547 | 0.021885758 |
| ENSMUSG00000042087 | 4933440N22Rik | 0.419545247 | 1.298340214 | 0.021888319 |
| ENSMUSG00000038650 | Rnh1 | -0.182307417 | 9.135183604 | 0.021912378 |
| ENSMUSG00000040613 | Apobec1 | -0.127504793 | 8.619956205 | 0.021958222 |
| ENSMUSG00000032609 | Klhdc8b | 0.241381919 | 4.665535321 | 0.021960083 |
| ENSMUSG00000034165 | Ccnd3 | 0.14648715 | 6.575584001 | 0.021965767 |
| ENSMUSG00000020325 | Fstl3 | 0.248550852 | 4.306968061 | 0.022015462 |
| ENSMUSG00000019806 | Aig1 | -0.195699811 | 4.662051659 | 0.022062389 |
| ENSMUSG00000074342 | I830077J02Rik | -0.249908633 | 2.618540405 | 0.022074752 |
| ENSMUSG00000029815 | Malsu1 | 0.175452868 | 3.946956352 | 0.022110361 |
| ENSMUSG00000018008 | Cyth4 | -0.145005646 | 9.484271644 | 0.022157445 |
| ENSMUSG00000087624 | 9230111E07Rik | 0.450308408 | 0.925060559 | 0.022186262 |
| ENSMUSG00000053641 | Dennd4a | 0.260926313 | 4.277366632 | 0.022281494 |
| ENSMUSG00000018848 | Rars | -0.14868522 | 6.429778283 | 0.022295637 |
| ENSMUSG00000022864 | D16Ertd472e | 0.379455224 | 1.830318892 | 0.022303023 |
| ENSMUSG00000029664 | Tfpi2 | 0.370926304 | 2.495652525 | 0.022338753 |
| ENSMUSG00000036661 | Dennd3 | 0.243636664 | 3.045734209 | 0.022344212 |
| ENSMUSG00000064147 | Rab44 | -0.593830458 | 1.131902584 | 0.022387242 |
| ENSMUSG00000033576 | Apol6 | 0.232625142 | 7.983223168 | 0.022410806 |
| ENSMUSG00000037280 | Galnt6 | -0.159243114 | 6.72851571 | 0.022417155 |
| ENSMUSG00000086915 | Gm16365 | 0.736890403 | -0.499750475 | 0.022520767 |
| ENSMUSG00000037012 | Hk1 | -0.139230076 | 6.815916722 | 0.022547205 |
| ENSMUSG00000054226 | Tprkb | -0.168013648 | 4.58970699 | 0.022561656 |
| ENSMUSG00000026134 | Prim2 | -0.190426427 | 4.019688274 | 0.022590817 |
| ENSMUSG00000041567 | Serpina12 | 0.714351489 | 0.081202855 | 0.022601635 |
| ENSMUSG00000028337 | Coro2a | -0.171231403 | 5.971351019 | 0.022670228 |
| ENSMUSG00000006522 | Itih3 | 0.562476606 | 0.267105733 | 0.022674565 |
| ENSMUSG00000027075 | Slc43a1 | 0.252790957 | 3.273549488 | 0.022694854 |
| ENSMUSG00000035403 | Crb2 | -0.799105208 | 0.037937222 | 0.022697399 |
| ENSMUSG00000042804 | Gpr153 | -0.225837133 | 5.348088541 | 0.022699326 |
| ENSMUSG00000028378 | Ptgr1 | -0.355608734 | 6.11346936 | 0.022714527 |
| ENSMUSG00000028636 | Ppcs | 0.227992468 | 3.605525301 | 0.022781314 |
| ENSMUSG00000009281 | Rarres2 | 0.172179919 | 8.869695752 | 0.022790535 |
| ENSMUSG00000031604 | Msmo1 | -0.1580935 | 5.981872232 | 0.022829434 |
| ENSMUSG00000063856 | Gpx1 | -0.156711449 | 10.6208203 | 0.022838635 |
| ENSMUSG00000066595 | Flvcr1 | -0.219365288 | 4.380835102 | 0.02291833 |
| ENSMUSG00000111977 | Gm47163 | -0.381250578 | 1.996611898 | 0.022989057 |
| ENSMUSG00000031097 | Tnni2 | -0.349756776 | 1.792907551 | 0.023028238 |
| ENSMUSG00000026939 | Tmem141 | -0.14844565 | 5.115914932 | 0.023090644 |
| ENSMUSG00000028581 | Laptm5 | -0.144104662 | 10.34096568 | 0.023123287 |
| ENSMUSG00000022270 | Retreg1 | -0.251755809 | 3.238911523 | 0.02312886 |
| ENSMUSG00000032285 | Dnaja4 | 0.237812908 | 3.128045946 | 0.023149157 |
| ENSMUSG00000029551 | Psmg3 | -0.238038505 | 3.64366186 | 0.023191832 |
| ENSMUSG00000071253 | Slc25a16 | 0.17694888 | 4.149995155 | 0.023243643 |
| ENSMUSG00000026664 | Phyh | 0.147121709 | 6.143218384 | 0.023248687 |
| ENSMUSG00000030282 | Cmas | -0.147881254 | 5.979981883 | 0.023258683 |
| ENSMUSG00000027674 | Pex5l | 0.377998302 | 4.390525629 | 0.023266099 |
| ENSMUSG00000054582 | Pabpc1l | 0.425054197 | 2.14762752 | 0.023275941 |
| ENSMUSG00000034187 | Nsf | -0.145920641 | 6.555545616 | 0.023281938 |
| ENSMUSG00000059201 | Lep | 0.28197845 | 10.74914081 | 0.023287545 |
| ENSMUSG00000031373 | Car5b | 0.218429639 | 5.828493111 | 0.023323259 |
| ENSMUSG00000023979 | Guca1b | -0.584507839 | -0.038391513 | 0.023333222 |
| ENSMUSG00000029776 | Hibadh | 0.147411935 | 6.456248441 | 0.023343402 |
| ENSMUSG00000018651 | Tada2a | -0.188370054 | 3.524832681 | 0.023416939 |
| ENSMUSG00000071359 | Tbpl1 | -0.150124709 | 4.668761294 | 0.023427955 |
| ENSMUSG00000097123 | Gm6297 | 0.682510169 | -0.271250572 | 0.023439751 |
| ENSMUSG00000020354 | Sgcd | 0.235646391 | 4.084836928 | 0.02349218 |
| ENSMUSG00000045672 | Col27a1 | -0.230133562 | 4.342139521 | 0.023558229 |
| ENSMUSG00000046806 | Cyren | -0.170224233 | 4.103571306 | 0.023657681 |
| ENSMUSG00000081763 | Gm9435 | 0.634044232 | -0.051082772 | 0.023675862 |
| ENSMUSG00000028931 | Kcnab2 | -0.179406428 | 7.058251005 | 0.023685767 |
| ENSMUSG00000029484 | Anxa3 | -0.197751756 | 7.276462483 | 0.023686676 |
| ENSMUSG00000023032 | Slc4a8 | 0.535449185 | 1.670510682 | 0.023717317 |
| ENSMUSG00000043587 | Pxylp1 | -0.283139976 | 2.562354527 | 0.023879228 |
| ENSMUSG00000036315 | Znrd1 | -0.165897637 | 4.384818984 | 0.023887712 |
| ENSMUSG00000025035 | Arl3 | -0.182595454 | 4.861061978 | 0.023893996 |
| ENSMUSG00000102748 | Pcdhgb2 | 0.480288344 | 0.901445371 | 0.023913144 |
| ENSMUSG00000020717 | Pecam1 | 0.202828764 | 7.46629469 | 0.023995111 |
| ENSMUSG00000022420 | Dnal4 | -0.152310129 | 5.434989927 | 0.024012731 |
| ENSMUSG00000061780 | Cfd | 0.485161079 | 7.181133383 | 0.024108529 |
| ENSMUSG00000054737 | Zfp182 | 0.244572005 | 2.847061378 | 0.024120697 |
| ENSMUSG00000058099 | Nfam1 | -0.194229748 | 6.927441773 | 0.024163423 |
| ENSMUSG00000006221 | Hspb7 | 0.29555188 | 6.797283243 | 0.024170832 |
| ENSMUSG00000036867 | Smad6 | 0.288217298 | 3.864937054 | 0.02421108 |
| ENSMUSG00000041828 | Abca8a | 0.218493564 | 4.80646028 | 0.024226781 |
| ENSMUSG00000027698 | Nceh1 | -0.150716568 | 7.300206142 | 0.024231516 |
| ENSMUSG00000021939 | Ctsb | -0.127878237 | 13.54404797 | 0.024232466 |
| ENSMUSG00000048779 | P2ry6 | -0.144419189 | 7.531303532 | 0.02425538 |
| ENSMUSG00000002332 | Dhrs1 | -0.104646363 | 6.738228057 | 0.024258728 |
| ENSMUSG00000105196 | Mir142 | -0.412390555 | 1.2790152 | 0.024280819 |
| ENSMUSG00000113769 | 5033406O09Rik | -0.344762276 | 1.948152838 | 0.02432392 |
| ENSMUSG00000020061 | Mybpc1 | 0.52463876 | 1.403672548 | 0.024326436 |
| ENSMUSG00000084925 | 1810062O18Rik | 0.500976389 | 0.533052858 | 0.024426782 |
| ENSMUSG00000027762 | Sucnr1 | 0.364800713 | 4.510096171 | 0.024431207 |
| ENSMUSG00000073489 | Ifi204 | 0.176085481 | 5.625447128 | 0.024528683 |
| ENSMUSG00000005417 | Mprip | 0.134316918 | 8.199393879 | 0.024558853 |
| ENSMUSG00000085355 | 3010003L21Rik | -0.766566255 | -0.120569545 | 0.024583724 |
| ENSMUSG00000068742 | Cry2 | 0.233175448 | 5.436090668 | 0.024605162 |
| ENSMUSG00000027222 | Pex16 | 0.180336481 | 7.103949885 | 0.02462459 |
| ENSMUSG00000025888 | Casp1 | -0.154623007 | 6.19568494 | 0.024644531 |
| ENSMUSG00000028121 | Bcar3 | 0.218538732 | 7.543138739 | 0.024680553 |
| ENSMUSG00000037936 | Scarb1 | 0.120164557 | 7.921809636 | 0.02476712 |
| ENSMUSG00000046245 | Pilra | -0.254755692 | 3.76015031 | 0.024782516 |
| ENSMUSG00000020143 | Dock2 | -0.161809123 | 8.050448099 | 0.024880411 |
| ENSMUSG00000031391 | L1cam | -0.231120042 | 3.405426688 | 0.024900883 |
| ENSMUSG00000023031 | Cela1 | 0.222826155 | 6.297331337 | 0.024951555 |
| ENSMUSG00000035246 | Pcyt1b | -0.365774379 | 1.488596045 | 0.025030865 |
| ENSMUSG00000040345 | Arhgap9 | -0.146248282 | 7.331341288 | 0.025039511 |
| ENSMUSG00000058715 | Fcer1g | -0.145030029 | 8.965158841 | 0.02508345 |
| ENSMUSG00000037788 | Vopp1 | -0.178959907 | 5.069072817 | 0.025162311 |
| ENSMUSG00000067203 | H2-K2 | -0.217718594 | 3.671449817 | 0.025209654 |
| ENSMUSG00000067224 | Gm3695 | 0.86672366 | -0.753145265 | 0.025230989 |
| ENSMUSG00000041801 | Phlda3 | 0.18322934 | 7.915269636 | 0.025309235 |
| ENSMUSG00000041836 | Ptpre | -0.186566386 | 5.956118573 | 0.025310584 |
| ENSMUSG00000017817 | Jph2 | 0.460460948 | 1.365604121 | 0.025338785 |
| ENSMUSG00000020644 | Id2 | -0.144424507 | 5.386517575 | 0.025361402 |
| ENSMUSG00000040373 | Cacng5 | 0.573213755 | 0.300575835 | 0.025380032 |
| ENSMUSG00000035041 | Creb3l3 | -0.310856683 | 2.909322926 | 0.025381515 |
| ENSMUSG00000096981 | Gm16845 | -0.302429609 | 2.659661058 | 0.025412054 |
| ENSMUSG00000033707 | Lrrc24 | 0.195153641 | 4.672241197 | 0.025460724 |
| ENSMUSG00000031618 | Nr3c2 | 0.308400025 | 1.770433142 | 0.025475724 |
| ENSMUSG00000021260 | Hhipl1 | 0.281395089 | 3.297657854 | 0.025489877 |
| ENSMUSG00000060038 | Dhps | -0.14180661 | 5.362649581 | 0.025546473 |
| ENSMUSG00000020023 | Tmcc3 | 0.161247183 | 5.913609871 | 0.025575315 |
| ENSMUSG00000021969 | Zdhhc20 | -0.155224282 | 5.230723108 | 0.025580392 |
| ENSMUSG00000044811 | Cd300c2 | -0.226742574 | 7.631328609 | 0.025585946 |
| ENSMUSG00000075296 | Aldh3b2 | 0.335230833 | 4.130131043 | 0.025612884 |
| ENSMUSG00000036782 | Klhl13 | 0.263409792 | 3.550524854 | 0.025629376 |
| ENSMUSG00000038156 | Spon1 | -0.263265251 | 4.840750646 | 0.025632333 |
| ENSMUSG00000005533 | Igf1r | 0.163913356 | 3.971032446 | 0.025635877 |
| ENSMUSG00000020553 | Pctp | -0.142456216 | 5.326894527 | 0.025669669 |
| ENSMUSG00000030505 | Prmt3 | -0.179696071 | 4.469922318 | 0.025758923 |
| ENSMUSG00000030987 | Stim1 | 0.122839621 | 6.378168361 | 0.025851032 |
| ENSMUSG00000040562 | Gstm2 | 0.171201298 | 6.079471025 | 0.025875862 |
| ENSMUSG00000109511 | Nup62 | -0.139072432 | 6.180265498 | 0.025932261 |
| ENSMUSG00000112134 | Gm40723 | -0.663331882 | 0.171875639 | 0.025971 |
| ENSMUSG00000039206 | Daglb | -0.14078649 | 8.190613292 | 0.025981813 |
| ENSMUSG00000026443 | Lrrn2 | -0.250742078 | 4.334321015 | 0.026079948 |
| ENSMUSG00000097277 | 2900076A07Rik | 0.269489851 | 2.386514118 | 0.026091453 |
| ENSMUSG00000057969 | Sema3b | 0.326008588 | 2.608279073 | 0.02613135 |
| ENSMUSG00000097177 | 9330159M07Rik | 0.505433475 | 1.251953488 | 0.02620252 |
| ENSMUSG00000014030 | Pax5 | 0.811533568 | 0.15466644 | 0.026220543 |
| ENSMUSG00000017664 | Slc35c2 | -0.131838299 | 6.121547151 | 0.026280222 |
| ENSMUSG00000040907 | Atp1a3 | -0.227869062 | 8.435193877 | 0.026360433 |
| ENSMUSG00000069910 | Spdl1 | -0.221907563 | 3.485099765 | 0.026368386 |
| ENSMUSG00000021477 | Ctsl | -0.169101922 | 11.51502974 | 0.026386324 |
| ENSMUSG00000029163 | Emilin1 | -0.175789704 | 7.082207351 | 0.026389941 |
| ENSMUSG00000050390 | C77080 | -0.207006921 | 7.433461351 | 0.026401023 |
| ENSMUSG00000033060 | Lmo7 | 0.321048528 | 3.185040243 | 0.026404428 |
| ENSMUSG00000058818 | Pirb | -0.158072213 | 8.579331803 | 0.026410081 |
| ENSMUSG00000031146 | Plp2 | -0.187944059 | 6.127498866 | 0.026454229 |
| ENSMUSG00000020190 | Mknk2 | 0.128473715 | 8.499504204 | 0.026471846 |
| ENSMUSG00000022510 | Trp63 | 0.596525198 | 0.117391936 | 0.026477811 |
| ENSMUSG00000023206 | Il15ra | 0.198184821 | 4.401872489 | 0.026487386 |
| ENSMUSG00000069769 | Msi2 | 0.204155287 | 3.944157284 | 0.026528905 |
| ENSMUSG00000006304 | Arpc2 | -0.107207082 | 9.214319191 | 0.026533332 |
| ENSMUSG00000022272 | Myo10 | 0.150860541 | 6.09162624 | 0.026534551 |
| ENSMUSG00000024713 | Pcsk5 | 0.318689058 | 3.388509875 | 0.026550052 |
| ENSMUSG00000031101 | Sash3 | -0.153536597 | 6.309640568 | 0.026600374 |
| ENSMUSG00000049382 | Krt8 | 0.733836392 | 0.550921124 | 0.0266496 |
| ENSMUSG00000031153 | Gripap1 | -0.152912798 | 6.364505467 | 0.026689566 |
| ENSMUSG00000031523 | Dlc1 | 0.176142139 | 6.926850719 | 0.026698444 |
| ENSMUSG00000014782 | Plekhg4 | -0.602571426 | -0.100527508 | 0.026700539 |
| ENSMUSG00000096974 | Gm26881 | 0.293599772 | 1.99277365 | 0.0268474 |
| ENSMUSG00000015354 | Pcolce2 | 0.23873129 | 5.254332611 | 0.026891572 |
| ENSMUSG00000026426 | Arl8a | -0.136477958 | 7.536703563 | 0.02698355 |
| ENSMUSG00000060044 | Tmem26 | -0.629329106 | 0.550784855 | 0.026998313 |
| ENSMUSG00000085586 | Gm11613 | -0.28505004 | 2.56956834 | 0.027069721 |
| ENSMUSG00000074093 | Svip | 0.263702407 | 3.112958395 | 0.027111114 |
| ENSMUSG00000020282 | Rhbdf1 | 0.139923773 | 6.414445601 | 0.027169977 |
| ENSMUSG00000030972 | Acsm5 | 0.671085844 | 0.323400718 | 0.027170617 |
| ENSMUSG00000059412 | Fxyd2 | -0.212692736 | 4.315932106 | 0.027193675 |
| ENSMUSG00000026672 | Optn | 0.197996301 | 6.075387124 | 0.027208305 |
| ENSMUSG00000112129 | Pbld1 | 0.552019648 | 0.541389957 | 0.02722031 |
| ENSMUSG00000079037 | Prnp | 0.143871185 | 8.047610445 | 0.027223609 |
| ENSMUSG00000026172 | Bcs1l | -0.156305863 | 3.99752828 | 0.027226418 |
| ENSMUSG00000015981 | Stk32c | -0.299863634 | 1.878824014 | 0.027233772 |
| ENSMUSG00000105176 | Gm43668 | 0.682011786 | -0.062302652 | 0.027243777 |
| ENSMUSG00000017009 | Sdc4 | 0.138887168 | 6.672344984 | 0.027283043 |
| ENSMUSG00000097729 | 2310015A10Rik | 0.237280886 | 3.15862031 | 0.027334348 |
| ENSMUSG00000023064 | Sncg | 0.24162353 | 9.678821581 | 0.02734363 |
| ENSMUSG00000041420 | Meis3 | -0.15181678 | 5.5301501 | 0.02735595 |
| ENSMUSG00000037824 | Tspan14 | -0.129697493 | 6.808099661 | 0.027357878 |
| ENSMUSG00000036334 | Igsf10 | -0.198841762 | 4.368126194 | 0.027361536 |
| ENSMUSG00000001946 | Esam | 0.189526938 | 6.558345644 | 0.027389247 |
| ENSMUSG00000025971 | Maip1 | 0.177588253 | 4.090140447 | 0.027392761 |
| ENSMUSG00000066952 | Myo1h | 0.478043778 | 0.593417747 | 0.027405792 |
| ENSMUSG00000064202 | 4430402I18Rik | 0.339669365 | 1.890962709 | 0.027412223 |
| ENSMUSG00000046711 | Hmga1 | -0.189192494 | 6.216933964 | 0.027429689 |
| ENSMUSG00000054434 | Tmem120b | 0.2021795 | 6.240248316 | 0.027439012 |
| ENSMUSG00000019087 | Atp6ap1 | -0.129875499 | 8.739562631 | 0.027482742 |
| ENSMUSG00000026208 | Des | 0.149134735 | 5.628833742 | 0.027534994 |
| ENSMUSG00000074227 | Spint2 | 0.228963487 | 4.023822125 | 0.027551378 |
| ENSMUSG00000079588 | Tmem182 | 0.301764782 | 5.958358367 | 0.027567331 |
| ENSMUSG00000030770 | Parva | 0.188097686 | 6.846079601 | 0.027609215 |
| ENSMUSG00000006542 | Prkag3 | 0.383571271 | 2.212420773 | 0.027702349 |
| ENSMUSG00000038451 | Spsb2 | -0.182115749 | 5.398911218 | 0.027799991 |
| ENSMUSG00000004626 | Stxbp2 | -0.117955769 | 7.541725149 | 0.027800535 |
| ENSMUSG00000023015 | Racgap1 | -0.14017303 | 4.931901952 | 0.027833173 |
| ENSMUSG00000029427 | Zcchc8 | -0.136985853 | 5.48344879 | 0.027874975 |
| ENSMUSG00000039270 | Megf9 | 0.217176666 | 4.50879595 | 0.027877436 |
| ENSMUSG00000040424 | Hipk4 | 0.535876373 | 0.186413293 | 0.02789124 |
| ENSMUSG00000064105 | Cnnm2 | 0.214740578 | 5.309551293 | 0.027923095 |
| ENSMUSG00000002233 | Rhoc | -0.14553415 | 8.819355661 | 0.027950844 |
| ENSMUSG00000023994 | Nfya | -0.150189563 | 6.238575365 | 0.027980828 |
| ENSMUSG00000006728 | Cdk4 | -0.121975688 | 7.21421173 | 0.027990928 |
| ENSMUSG00000097534 | Gm16675 | 0.583108321 | 0.545557226 | 0.028107266 |
| ENSMUSG00000026705 | Klhl20 | -0.172724027 | 4.198710196 | 0.028164496 |
| ENSMUSG00000029684 | Wasl | 0.164826224 | 4.735212978 | 0.028245998 |
| ENSMUSG00000020818 | Mfsd11 | -0.118839555 | 6.861652462 | 0.028260761 |
| ENSMUSG00000026249 | Serpine2 | 0.157339852 | 6.134151077 | 0.028271398 |
| ENSMUSG00000022485 | Hoxc5 | 0.270912569 | 3.040591679 | 0.028274783 |
| ENSMUSG00000020059 | Sycp3 | 0.360229214 | 4.137185666 | 0.028300895 |
| ENSMUSG00000015536 | Mocs2 | 0.117841957 | 6.460316253 | 0.028344922 |
| ENSMUSG00000042082 | Arsb | -0.123440787 | 5.877314512 | 0.028348455 |
| ENSMUSG00000028958 | Tmub1 | -0.214445453 | 3.590720234 | 0.028392011 |
| ENSMUSG00000105377 | Gm43148 | 0.702789762 | -0.442252742 | 0.028395217 |
| ENSMUSG00000043673 | Kcns3 | 0.350976415 | 3.251677102 | 0.02842078 |
| ENSMUSG00000079018 | Ly6c1 | 0.157961093 | 6.482414828 | 0.028430109 |
| ENSMUSG00000000811 | Txnrd3 | 0.191541059 | 4.060660353 | 0.028465358 |
| ENSMUSG00000027962 | Vcam1 | 0.247391202 | 5.854649112 | 0.028494231 |
| ENSMUSG00000031659 | Adcy7 | -0.139006478 | 7.512036701 | 0.028518351 |
| ENSMUSG00000060675 | Pla2g16 | 0.238994893 | 8.576732615 | 0.028527036 |
| ENSMUSG00000010914 | Pdhx | 0.180576693 | 5.084218951 | 0.028555646 |
| ENSMUSG00000027546 | Atp9a | 0.183466947 | 7.494873951 | 0.02856013 |
| ENSMUSG00000060429 | Sntb1 | 0.257215765 | 4.162596608 | 0.028571725 |
| ENSMUSG00000031299 | Pdha1 | 0.134854406 | 7.087106864 | 0.028604291 |
| ENSMUSG00000016256 | Ctsz | -0.161718444 | 10.60263788 | 0.028643576 |
| ENSMUSG00000040061 | Plcb2 | -0.254516705 | 3.786589129 | 0.028665971 |
| ENSMUSG00000050428 | Fbxo46 | -0.436075606 | 3.881009927 | 0.028684456 |
| ENSMUSG00000075419 | Dolk | 0.150561582 | 6.049790141 | 0.028687422 |
| ENSMUSG00000042759 | Apobr | -0.138763501 | 7.410204281 | 0.028694713 |
| ENSMUSG00000082292 | Gm12250 | 0.430449721 | 1.653597466 | 0.028704886 |
| ENSMUSG00000088595 | Gm24601 | -0.832071081 | 0.505829051 | 0.02871075 |
| ENSMUSG00000008393 | Carhsp1 | 0.151716376 | 7.125568756 | 0.028784484 |
| ENSMUSG00000028932 | Psmc2 | -0.105892892 | 7.432994556 | 0.028841339 |
| ENSMUSG00000032850 | Rnft2 | 0.288136788 | 2.475573639 | 0.028844335 |
| ENSMUSG00000039982 | Dtx4 | -0.119537072 | 6.774802734 | 0.028863841 |
| ENSMUSG00000018927 | Ccl6 | -0.212923337 | 9.332210877 | 0.028990302 |
| ENSMUSG00000035385 | Ccl2 | -0.28224529 | 6.446997411 | 0.029009902 |
| ENSMUSG00000020836 | Coro6 | 0.379013066 | 3.093806366 | 0.029017452 |
| ENSMUSG00000039601 | Rcan2 | 0.407489734 | 2.526719659 | 0.029051199 |
| ENSMUSG00000029094 | Afap1 | 0.214578645 | 4.974025215 | 0.029056818 |
| ENSMUSG00000037347 | Chst7 | 0.31899492 | 2.809350003 | 0.029088446 |
| ENSMUSG00000030787 | Lyve1 | 0.868480526 | 1.241448518 | 0.029102363 |
| ENSMUSG00000035069 | Oma1 | -0.162734006 | 4.473758605 | 0.029168427 |
| ENSMUSG00000046714 | Foxc2 | 0.580156015 | 0.309147753 | 0.029188547 |
| ENSMUSG00000040824 | Snrpd2 | -0.177191081 | 5.177681056 | 0.029324309 |
| ENSMUSG00000026785 | Pkn3 | 0.184543294 | 4.543717759 | 0.029344048 |
| ENSMUSG00000022607 | Ptk2 | 0.116651009 | 5.480326453 | 0.029392642 |
| ENSMUSG00000038296 | Galnt18 | 0.216988468 | 4.062302572 | 0.029416373 |
| ENSMUSG00000028907 | Utp11 | -0.171993263 | 5.439040046 | 0.029478961 |
| ENSMUSG00000046034 | Otulin | -0.130577601 | 6.05013725 | 0.029496436 |
| ENSMUSG00000054702 | Ap1s3 | -0.448285437 | 1.224694448 | 0.029535945 |
| ENSMUSG00000032332 | Col12a1 | 0.303540878 | 4.732866594 | 0.029552105 |
| ENSMUSG00000091512 | Lamtor3 | -0.153488137 | 5.268859449 | 0.029554009 |
| ENSMUSG00000033530 | Ttc7b | 0.145760699 | 6.553237067 | 0.029576815 |
| ENSMUSG00000053024 | Cntn2 | 0.536068985 | 3.471555578 | 0.029579133 |
| ENSMUSG00000042686 | Jph1 | 0.401505869 | 1.879906532 | 0.029592822 |
| ENSMUSG00000026600 | Soat1 | -0.159378078 | 7.321153229 | 0.029667901 |
| ENSMUSG00000022555 | Dgat1 | 0.19474691 | 6.975420506 | 0.029694439 |
| ENSMUSG00000041845 | Rhod | 0.206846372 | 3.82677945 | 0.029696135 |
| ENSMUSG00000024677 | Ms4a6b | 0.284652836 | 4.978670757 | 0.029699238 |
| ENSMUSG00000062488 | Ifit3b | 0.421052075 | 2.411588144 | 0.02982296 |
| ENSMUSG00000042476 | Abcb4 | -0.172475759 | 4.418565812 | 0.029841777 |
| ENSMUSG00000109118 | Gm32031 | -0.373900147 | 1.317548506 | 0.02985496 |
| ENSMUSG00000075284 | Wipf1 | -0.123214084 | 6.460915231 | 0.029887271 |
| ENSMUSG00000049858 | Suox | 0.241697253 | 3.364879687 | 0.029889821 |
| ENSMUSG00000049643 | 2310022A10Rik | -0.127982686 | 6.648879816 | 0.029922423 |
| ENSMUSG00000041378 | Cldn5 | 0.31133619 | 6.246633146 | 0.029924272 |
| ENSMUSG00000002799 | Jag2 | 0.287220197 | 4.03057059 | 0.029995214 |
| ENSMUSG00000028150 | Rorc | 0.62598485 | 0.520268241 | 0.030097221 |
| ENSMUSG00000008575 | Nfib | 0.163646231 | 5.968780902 | 0.030152969 |
| ENSMUSG00000044162 | Tnip3 | -0.182489083 | 5.262743072 | 0.030182128 |
| ENSMUSG00000034205 | Loxl2 | -0.20829095 | 6.218622791 | 0.030243408 |
| ENSMUSG00000026435 | Slc45a3 | -0.274038873 | 3.870098509 | 0.030243707 |
| ENSMUSG00000027944 | Hax1 | -0.185298039 | 3.936326738 | 0.030306369 |
| ENSMUSG00000038146 | Notch3 | 0.190521792 | 6.135364945 | 0.030369845 |
| ENSMUSG00000031984 | 2810004N23Rik | -0.153937665 | 4.686456157 | 0.030380865 |
| ENSMUSG00000027712 | Anxa5 | -0.10900414 | 10.12312724 | 0.03039462 |
| ENSMUSG00000029313 | Aff1 | 0.108757265 | 5.800786091 | 0.030424172 |
| ENSMUSG00000021114 | Atp6v1d | -0.166460032 | 6.57097389 | 0.03051372 |
| ENSMUSG00000026048 | Ercc5 | 0.178662252 | 4.45396191 | 0.030529244 |
| ENSMUSG00000048327 | Ckap2l | -0.23918484 | 3.565894821 | 0.030551263 |
| ENSMUSG00000039349 | C130074G19Rik | 0.22970717 | 8.121679853 | 0.030611211 |
| ENSMUSG00000032352 | Lrrc1 | 0.285534282 | 2.63200913 | 0.030662281 |
| ENSMUSG00000078771 | Evi2a | -0.13672638 | 6.635780777 | 0.030701765 |
| ENSMUSG00000061740 | Cyp2d22 | 0.221599188 | 5.228276534 | 0.030713263 |
| ENSMUSG00000040310 | Alx4 | -0.439310363 | 2.47986993 | 0.030741271 |
| ENSMUSG00000024953 | Prdx5 | 0.111875899 | 8.560104673 | 0.03075924 |
| ENSMUSG00000057367 | Birc2 | 0.157821522 | 4.485989137 | 0.03077673 |
| ENSMUSG00000042256 | Ptchd4 | 0.503777327 | 0.859888426 | 0.030788133 |
| ENSMUSG00000026526 | Fh1 | 0.12409532 | 6.534749484 | 0.030797049 |
| ENSMUSG00000087142 | Gm12454 | -0.482576141 | 0.872592472 | 0.030868265 |
| ENSMUSG00000085360 | Arhgap27os2 | -0.701159316 | -0.432622808 | 0.030996977 |
| ENSMUSG00000040528 | Milr1 | -0.166152452 | 5.127976997 | 0.031015722 |
| ENSMUSG00000043091 | Tuba1c | -0.124759717 | 6.513564163 | 0.031065417 |
| ENSMUSG00000048731 | Ggnbp1 | 0.305432918 | 2.389632801 | 0.031069401 |
| ENSMUSG00000033788 | Dysf | 0.184528988 | 5.421662766 | 0.031091042 |
| ENSMUSG00000055013 | Agap1 | -0.145260811 | 6.165365233 | 0.031109895 |
| ENSMUSG00000061723 | Tnnt3 | 0.566819983 | 0.482360826 | 0.031160968 |
| ENSMUSG00000042700 | Sipa1l1 | 0.159875892 | 6.580332981 | 0.031202031 |
| ENSMUSG00000028773 | Fabp3 | 0.519498795 | 0.733355532 | 0.03120875 |
| ENSMUSG00000048355 | Arxes1 | 0.227001484 | 3.885933126 | 0.031273698 |
| ENSMUSG00000020175 | Rab36 | 0.369216093 | 1.350900392 | 0.031292113 |
| ENSMUSG00000018001 | Cyth3 | 0.15414874 | 6.440790814 | 0.031324023 |
| ENSMUSG00000030094 | Xpc | 0.139441767 | 4.895451148 | 0.031346313 |
| ENSMUSG00000027834 | Serpini1 | 0.630763108 | -0.266849647 | 0.031396565 |
| ENSMUSG00000112352 | Gm40617 | 0.544538201 | 0.496214608 | 0.031396919 |
| ENSMUSG00000070867 | Trabd2b | 0.308701207 | 5.076834593 | 0.031406164 |
| ENSMUSG00000025465 | Echs1 | 0.137547241 | 6.96508431 | 0.03143277 |
| ENSMUSG00000039428 | Tmem135 | 0.228034296 | 6.743265878 | 0.031459407 |
| ENSMUSG00000064043 | Trerf1 | -0.139139814 | 5.620523618 | 0.031496483 |
| ENSMUSG00000046949 | Nqo2 | -0.143264698 | 5.559128026 | 0.031536339 |
| ENSMUSG00000072653 | Zfp783 | 0.265439292 | 2.465904381 | 0.031554931 |
| ENSMUSG00000029287 | Tgfbr3 | 0.155694517 | 5.707223766 | 0.031630367 |
| ENSMUSG00000029771 | Irf5 | -0.129128692 | 7.94045967 | 0.031638885 |
| ENSMUSG00000038086 | Hspb2 | 0.323060001 | 3.146495064 | 0.031644439 |
| ENSMUSG00000038147 | Cd84 | -0.164031598 | 8.023526223 | 0.031644655 |
| ENSMUSG00000032527 | Pccb | 0.167419106 | 6.6489157 | 0.03166285 |
| ENSMUSG00000098098 | Bvht | -0.24608633 | 3.163745742 | 0.031670945 |
| ENSMUSG00000036172 | Cd200r3 | -0.640921026 | 0.459569143 | 0.031690193 |
| ENSMUSG00000048988 | Elfn1 | -0.517436258 | 0.466983734 | 0.031809915 |
| ENSMUSG00000040339 | Fam102b | 0.151780852 | 5.60032578 | 0.031830858 |
| ENSMUSG00000101389 | Ms4a4a | 0.384397139 | 2.861390663 | 0.031857383 |
| ENSMUSG00000090698 | Apold1 | 0.314948599 | 2.309718406 | 0.031875122 |
| ENSMUSG00000107634 | Gm36816 | 0.591623252 | 0.222657652 | 0.031945291 |
| ENSMUSG00000022340 | Sybu | 0.355511846 | 2.262164014 | 0.032088987 |
| ENSMUSG00000036985 | Zdhhc9 | -0.125134713 | 6.534474258 | 0.032115872 |
| ENSMUSG00000051853 | Arf3 | -0.109991649 | 7.570720261 | 0.032124957 |
| ENSMUSG00000023132 | Gzma | 0.481013821 | 1.474956729 | 0.032127951 |
| ENSMUSG00000022843 | Clcn2 | 0.275694543 | 2.705142448 | 0.032153502 |
| ENSMUSG00000038418 | Egr1 | -0.209238464 | 5.248634472 | 0.032157004 |
| ENSMUSG00000063760 | Rnf217 | -0.235506905 | 3.193411197 | 0.032161656 |
| ENSMUSG00000021428 | Riok1 | -0.152880288 | 4.446439491 | 0.032211468 |
| ENSMUSG00000047904 | Sstr2 | -0.406546658 | 1.691995331 | 0.03225035 |
| ENSMUSG00000035735 | Dagla | -0.15168471 | 4.903337443 | 0.032292347 |
| ENSMUSG00000022878 | Adipoq | 0.26890953 | 9.522130287 | 0.032293702 |
| ENSMUSG00000052748 | Swt1 | -0.212514384 | 2.996034393 | 0.032308551 |
| ENSMUSG00000024534 | Sncaip | 0.306362096 | 2.666689688 | 0.032341718 |
| ENSMUSG00000037196 | Pacrg | -0.350329531 | 2.106284191 | 0.032375378 |
| ENSMUSG00000020422 | Tns3 | -0.130312057 | 7.066284349 | 0.032382276 |
| ENSMUSG00000084989 | Crocc2 | 0.54963707 | 0.062340288 | 0.032383096 |
| ENSMUSG00000029217 | Tec | -0.18616154 | 5.427880982 | 0.032406872 |
| ENSMUSG00000006764 | Tph2 | 0.353756006 | 5.819097311 | 0.032454935 |
| ENSMUSG00000029082 | Bst1 | -0.258014371 | 4.837892915 | 0.03248314 |
| ENSMUSG00000038668 | Lpar1 | 0.238235842 | 3.774258641 | 0.03262032 |
| ENSMUSG00000036158 | Prickle1 | 0.31148084 | 2.249856553 | 0.032633186 |
| ENSMUSG00000020682 | Mmp28 | 0.227267925 | 5.060591928 | 0.032651695 |
| ENSMUSG00000028538 | St3gal3 | 0.150402247 | 6.082310449 | 0.03265592 |
| ENSMUSG00000026192 | Atic | -0.110420046 | 6.080867457 | 0.032666157 |
| ENSMUSG00000028528 | Dnajc6 | 0.395658559 | 2.273116971 | 0.032823149 |
| ENSMUSG00000028863 | Meaf6 | -0.125171421 | 4.990220273 | 0.032846323 |
| ENSMUSG00000031841 | Cdh13 | 0.268779169 | 4.984837083 | 0.032867175 |
| ENSMUSG00000025545 | Clybl | 0.152795225 | 5.640729775 | 0.032936231 |
| ENSMUSG00000035642 | Aamdc | 0.158445122 | 4.788224357 | 0.03296672 |
| ENSMUSG00000027646 | Src | -0.162318153 | 5.17823408 | 0.033014813 |
| ENSMUSG00000032812 | Arap1 | -0.123458565 | 8.152014764 | 0.033105267 |
| ENSMUSG00000028358 | Zfp618 | 0.367287046 | 1.619383438 | 0.033119386 |
| ENSMUSG00000027015 | Cybrd1 | -0.688770139 | 0.453379484 | 0.033126426 |
| ENSMUSG00000051314 | Ffar2 | 0.226882947 | 5.633423369 | 0.033142814 |
| ENSMUSG00000004044 | Cavin1 | 0.154217743 | 10.25717565 | 0.033180448 |
| ENSMUSG00000026069 | Il1rl1 | -0.364760353 | 3.473479383 | 0.033382724 |
| ENSMUSG00000079610 | Ankrd39 | 0.203736107 | 3.090424349 | 0.033506371 |
| ENSMUSG00000012428 | Steap4 | 0.205733258 | 7.590019103 | 0.033517253 |
| ENSMUSG00000063142 | Kcnma1 | 0.698364545 | -0.459202813 | 0.033625897 |
| ENSMUSG00000036545 | Adamts2 | 0.121692696 | 7.948761758 | 0.033633049 |
| ENSMUSG00000054509 | Parp4 | -0.123277714 | 5.235172314 | 0.033639934 |
| ENSMUSG00000027309 | 4930402H24Rik | 0.147640863 | 6.341929655 | 0.03368749 |
| ENSMUSG00000004099 | Dnmt1 | -0.117982676 | 6.588977512 | 0.033848543 |
| ENSMUSG00000032735 | Ablim3 | 0.272713528 | 5.696182662 | 0.033912067 |
| ENSMUSG00000029778 | Adcyap1r1 | -0.136687568 | 5.869707557 | 0.03392574 |
| ENSMUSG00000041774 | Ydjc | -0.28669171 | 2.242297784 | 0.033962908 |
| ENSMUSG00000044477 | Zfand3 | 0.112565756 | 7.420270957 | 0.033997423 |
| ENSMUSG00000025792 | Slc25a10 | 0.177880936 | 8.31349199 | 0.03401911 |
| ENSMUSG00000071657 | Bscl2 | 0.158713829 | 8.208368702 | 0.034062649 |
| ENSMUSG00000035357 | Pdzrn3 | 0.217859803 | 4.536150061 | 0.03406276 |
| ENSMUSG00000073394 | Runx2os1 | 0.479671257 | 0.820173002 | 0.034147924 |
| ENSMUSG00000051790 | Nlgn2 | -0.129513568 | 4.984164057 | 0.03416312 |
| ENSMUSG00000030410 | Dmwd | 0.194750694 | 5.119301145 | 0.034219827 |
| ENSMUSG00000055407 | Map6 | 0.153574986 | 5.125773429 | 0.034257172 |
| ENSMUSG00000034751 | Mast4 | 0.183467284 | 3.949602793 | 0.034263797 |
| ENSMUSG00000111128 | Gm49338 | 0.261589836 | 2.544696609 | 0.03427187 |
| ENSMUSG00000028766 | Alpl | 0.301328138 | 2.967603597 | 0.034282474 |
| ENSMUSG00000024610 | Cd74 | 0.204102575 | 11.76813971 | 0.034305429 |
| ENSMUSG00000023828 | Slc22a3 | 0.271538181 | 5.314774452 | 0.034329048 |
| ENSMUSG00000014606 | Slc25a11 | -0.103286104 | 7.205678775 | 0.034351646 |
| ENSMUSG00000005374 | Tbl2 | 0.137381571 | 4.937439517 | 0.03436489 |
| ENSMUSG00000022751 | Nit2 | -0.153545437 | 4.722638501 | 0.034393451 |
| ENSMUSG00000022468 | Endou | 0.338757199 | 2.60386302 | 0.034428879 |
| ENSMUSG00000050471 | Fam118b | -0.165330597 | 4.733895873 | 0.034476349 |
| ENSMUSG00000026068 | Il18rap | 0.341584567 | 1.873528266 | 0.034536608 |
| ENSMUSG00000026547 | Tagln2 | -0.140463936 | 9.61251748 | 0.034563487 |
| ENSMUSG00000056973 | Ces1d | 0.305671446 | 6.359767167 | 0.034566317 |
| ENSMUSG00000005899 | Smpd4 | -0.140996181 | 5.43970892 | 0.03463402 |
| ENSMUSG00000059920 | 4930453N24Rik | 0.186923715 | 4.220559988 | 0.03466613 |
| ENSMUSG00000038807 | Rap1gap2 | 0.250707997 | 4.507569046 | 0.034709534 |
| ENSMUSG00000047686 | Rtl3 | -0.601767634 | 0.435208146 | 0.034776124 |
| ENSMUSG00000001227 | Sema6b | 0.18503397 | 5.239026715 | 0.034800519 |
| ENSMUSG00000067235 | H2-Q10 | 0.683596285 | 1.875014266 | 0.034943242 |
| ENSMUSG00000036452 | Arhgap26 | 0.235900037 | 3.440673103 | 0.034950363 |
| ENSMUSG00000030739 | Myh14 | 0.307662264 | 4.023611355 | 0.034972329 |
| ENSMUSG00000055240 | Zfp101 | 0.303114868 | 2.030484673 | 0.035026842 |
| ENSMUSG00000038366 | Lasp1 | -0.096890764 | 9.11321488 | 0.035057076 |
| ENSMUSG00000026958 | Dpp7 | -0.196291253 | 6.493819733 | 0.035064527 |
| ENSMUSG00000038525 | Armc10 | -0.169925339 | 4.354231676 | 0.035127765 |
| ENSMUSG00000004328 | Hif3a | 0.619702522 | 0.128887897 | 0.035201372 |
| ENSMUSG00000020089 | Ppa1 | 0.153126483 | 6.063322202 | 0.035246427 |
| ENSMUSG00000106928 | Gm43860 | 0.39753844 | 0.854935084 | 0.035275458 |
| ENSMUSG00000044201 | Cdc25c | -0.32017766 | 1.854825044 | 0.03535984 |
| ENSMUSG00000036667 | Tcaf1 | 0.190555841 | 5.916835271 | 0.035379661 |
| ENSMUSG00000115388 | Eppk1 | 0.600762398 | 0.000958837 | 0.035399485 |
| ENSMUSG00000068566 | Myadm | -0.126506265 | 8.128779745 | 0.035485225 |
| ENSMUSG00000051674 | Dcun1d4 | 0.235284172 | 3.847822775 | 0.035488665 |
| ENSMUSG00000068114 | Ccdc134 | 0.157001492 | 4.546285225 | 0.035490293 |
| ENSMUSG00000020184 | Mdm2 | -0.139806518 | 6.332717669 | 0.035527515 |
| ENSMUSG00000030798 | Cd37 | -0.140315663 | 7.715274975 | 0.035536961 |
| ENSMUSG00000021646 | Mccc2 | 0.16974139 | 5.312623006 | 0.03558993 |
| ENSMUSG00000086533 | Mypopos | -0.402523359 | 1.329449178 | 0.035644524 |
| ENSMUSG00000004814 | Ccl24 | 0.541443534 | 1.570807135 | 0.035648932 |
| ENSMUSG00000026620 | Mark1 | 0.219140901 | 4.008861797 | 0.035665671 |
| ENSMUSG00000024661 | Fth1 | -0.175567752 | 12.51191772 | 0.035680992 |
| ENSMUSG00000105449 | Gm43379 | 0.705235119 | -0.511982428 | 0.035700582 |
| ENSMUSG00000035863 | Palm | 0.197673804 | 6.549595953 | 0.03573118 |
| ENSMUSG00000024053 | Emilin2 | -0.125177782 | 7.185522901 | 0.035750616 |
| ENSMUSG00000020100 | Slc29a3 | -0.155684256 | 5.289201941 | 0.035769245 |
| ENSMUSG00000018507 | Trpv2 | -0.159755517 | 6.851787541 | 0.035793786 |
| ENSMUSG00000047867 | Gimap6 | 0.207046647 | 5.205867707 | 0.035801896 |
| ENSMUSG00000110744 | Gm5171 | -0.652464304 | -0.347486209 | 0.03582802 |
| ENSMUSG00000030281 | Il17rc | 0.15932148 | 4.566789042 | 0.035837441 |
| ENSMUSG00000037857 | Nufip2 | -0.192433759 | 3.958658456 | 0.035860776 |
| ENSMUSG00000048965 | Mrgpre | -0.215014967 | 3.687760025 | 0.035894909 |
| ENSMUSG00000043501 | Lgals2 | -0.705758882 | -0.47831306 | 0.036020886 |
| ENSMUSG00000045975 | C2cd2 | 0.215200486 | 6.234488169 | 0.03604636 |
| ENSMUSG00000043090 | Zfp866 | 0.238629244 | 3.012202709 | 0.036161167 |
| ENSMUSG00000014859 | E2f4 | -0.10757646 | 6.098157937 | 0.036167441 |
| ENSMUSG00000029602 | Rasal1 | -0.280779997 | 2.172849333 | 0.036189263 |
| ENSMUSG00000015027 | Galns | -0.141775069 | 6.954029691 | 0.036191905 |
| ENSMUSG00000024347 | Psd2 | -0.428708874 | 1.329850043 | 0.036195123 |
| ENSMUSG00000035849 | Krt222 | 0.410954852 | 1.207898851 | 0.036331121 |
| ENSMUSG00000036992 | Nxt1 | -0.160609305 | 4.002154638 | 0.036355079 |
| ENSMUSG00000022089 | Bin3 | -0.147909368 | 6.047384468 | 0.036372096 |
| ENSMUSG00000071714 | Csf2rb2 | -0.158693287 | 7.049342373 | 0.03637445 |
| ENSMUSG00000026131 | Dst | 0.213544869 | 7.570130412 | 0.036376274 |
| ENSMUSG00000079625 | Tm4sf19 | -0.245721826 | 4.213927049 | 0.036417977 |
| ENSMUSG00000032220 | Myo1e | -0.111296265 | 8.234235596 | 0.036441098 |
| ENSMUSG00000030352 | Tspan9 | 0.144709638 | 5.324047061 | 0.036559874 |
| ENSMUSG00000045211 | Nudt18 | 0.150670673 | 6.138801202 | 0.03656886 |
| ENSMUSG00000023169 | Slc38a1 | -0.168304564 | 4.986829804 | 0.036610786 |
| ENSMUSG00000055413 | H2-Q5 | 0.217403094 | 2.928209357 | 0.036637303 |
| ENSMUSG00000073147 | 5031425E22Rik | 0.172886202 | 3.85772508 | 0.036651669 |
| ENSMUSG00000039410 | Prdm16 | 0.3531824 | 2.506593491 | 0.036694733 |
| ENSMUSG00000045569 | Mc2r | 0.318798699 | 3.618909789 | 0.036705249 |
| ENSMUSG00000000628 | Hk2 | 0.193510134 | 5.230354674 | 0.036727765 |
| ENSMUSG00000067586 | S1pr3 | -0.153238262 | 5.205774322 | 0.03677355 |
| ENSMUSG00000015843 | Rxrg | 0.234979208 | 3.518553437 | 0.036782525 |
| ENSMUSG00000004266 | Ptpn6 | -0.144404158 | 7.723113817 | 0.036816724 |
| ENSMUSG00000021384 | Susd3 | -0.153073563 | 5.459925851 | 0.036856184 |
| ENSMUSG00000026833 | Olfm1 | 0.124830789 | 6.91441777 | 0.036954875 |
| ENSMUSG00000036537 | Rnf113a1 | -0.373309531 | 1.433233083 | 0.036969497 |
| ENSMUSG00000037148 | Arhgap10 | -0.149358516 | 6.720838818 | 0.036976818 |
| ENSMUSG00000019577 | Pdk4 | 0.41499625 | 5.406887029 | 0.036983704 |
| ENSMUSG00000029228 | Lnx1 | 0.402577456 | 2.399488815 | 0.037091758 |
| ENSMUSG00000070420 | Zscan25 | 0.179617383 | 3.789811792 | 0.037220586 |
| ENSMUSG00000000489 | Pdgfb | -0.17254677 | 6.655662492 | 0.037304808 |
| ENSMUSG00000040721 | Zfhx2 | -0.237665234 | 3.296263813 | 0.037324299 |
| ENSMUSG00000052911 | Lamb2 | 0.17169498 | 7.463021688 | 0.037366958 |
| ENSMUSG00000021573 | Tppp | 0.388245403 | 2.11557991 | 0.037465123 |
| ENSMUSG00000060568 | Fam78b | -0.221898125 | 4.154342779 | 0.03755385 |
| ENSMUSG00000029231 | Pdgfra | -0.154270378 | 5.706001474 | 0.037628333 |
| ENSMUSG00000045136 | Tubb2b | -0.21163002 | 3.907235777 | 0.037646055 |
| ENSMUSG00000013236 | Ptprs | -0.120625782 | 7.615082291 | 0.037679915 |
| ENSMUSG00000026072 | Il1r1 | -0.160299692 | 5.32444006 | 0.037691117 |
| ENSMUSG00000014453 | Blk | 0.625003541 | -0.234153005 | 0.037701329 |
| ENSMUSG00000098050 | Gm5345 | -0.49994877 | 0.23582798 | 0.03774782 |
| ENSMUSG00000040219 | Ttc12 | -0.195723353 | 4.832892478 | 0.037757636 |
| ENSMUSG00000063146 | Clip2 | -0.111449713 | 6.39468171 | 0.037772138 |
| ENSMUSG00000028463 | Car9 | -0.220539093 | 3.191431543 | 0.037784925 |
| ENSMUSG00000038172 | Ttc39b | 0.141499847 | 4.535390881 | 0.03778577 |
| ENSMUSG00000007987 | Ift22 | -0.128385173 | 5.074023476 | 0.037831682 |
| ENSMUSG00000085315 | A430018G15Rik | 0.434043126 | 1.400325225 | 0.037903493 |
| ENSMUSG00000052384 | Nrros | -0.14700537 | 7.919210449 | 0.037914449 |
| ENSMUSG00000022037 | Clu | 0.241092228 | 7.038046841 | 0.037988077 |
| ENSMUSG00000048096 | Lmod1 | 0.32438466 | 2.391240561 | 0.038017948 |
| ENSMUSG00000037035 | Inhbb | 0.244229337 | 5.505283448 | 0.038068939 |
| ENSMUSG00000018459 | Slc13a3 | -0.66012068 | 4.406459099 | 0.038095981 |
| ENSMUSG00000030206 | Gsg1 | -0.531106982 | 0.575374005 | 0.03814992 |
| ENSMUSG00000042570 | Mier2 | -0.131462847 | 5.70562731 | 0.038183919 |
| ENSMUSG00000053985 | Zfp14 | 0.300048685 | 2.504325059 | 0.03820351 |
| ENSMUSG00000032841 | Prr5l | -0.256399261 | 2.335873239 | 0.03822117 |
| ENSMUSG00000032946 | Rasgrp2 | 0.263531116 | 5.385503683 | 0.038227406 |
| ENSMUSG00000010080 | Epn3 | 0.670750205 | -0.252457899 | 0.038233868 |
| ENSMUSG00000041957 | Pkp2 | 0.190283127 | 5.614847302 | 0.038279776 |
| ENSMUSG00000037712 | Fermt2 | 0.246009143 | 6.058773828 | 0.038324109 |
| ENSMUSG00000097471 | 5830432E09Rik | -0.327972348 | 2.702989167 | 0.03833559 |
| ENSMUSG00000030255 | Sspn | 0.181638107 | 4.658777699 | 0.038427287 |
| ENSMUSG00000044562 | Rasip1 | 0.2484364 | 5.359755262 | 0.038433499 |
| ENSMUSG00000019944 | Rhobtb1 | 0.207459597 | 3.837421061 | 0.038456511 |
| ENSMUSG00000057880 | Abat | 0.24774225 | 4.321424352 | 0.03853223 |
| ENSMUSG00000041488 | Stx3 | -0.188392855 | 4.378125653 | 0.038579206 |
| ENSMUSG00000032679 | Cd59a | 0.189725513 | 6.573689206 | 0.038592269 |
| ENSMUSG00000001281 | Itgb7 | -0.212531687 | 5.341727801 | 0.038599022 |
| ENSMUSG00000043991 | Pura | -0.209753436 | 3.639483921 | 0.038607675 |
| ENSMUSG00000056201 | Cfl1 | -0.116490153 | 10.05614834 | 0.038653221 |
| ENSMUSG00000045534 | Kcna5 | 0.322260646 | 2.890530344 | 0.038710506 |
| ENSMUSG00000021250 | Fos | -0.14007829 | 7.082425053 | 0.038754613 |
| ENSMUSG00000031971 | Ccsap | -0.222114029 | 3.234853982 | 0.038759751 |
| ENSMUSG00000023034 | Nr4a1 | 0.182320713 | 4.018916666 | 0.038776281 |
| ENSMUSG00000024043 | Arhgap28 | -0.313348306 | 2.311860825 | 0.038794794 |
| ENSMUSG00000050619 | Zscan29 | -0.145922864 | 4.447450111 | 0.038822013 |
| ENSMUSG00000015355 | Cd48 | -0.138689664 | 6.626188778 | 0.038854675 |
| ENSMUSG00000013275 | Slc41a1 | 0.154449434 | 5.013003784 | 0.038880285 |
| ENSMUSG00000004730 | Adgre1 | -0.146150622 | 9.462599758 | 0.038883365 |
| ENSMUSG00000052040 | Klf13 | 0.162545148 | 6.514690287 | 0.038911958 |
| ENSMUSG00000047473 | Zfp30 | 0.375492883 | 1.378399178 | 0.038927393 |
| ENSMUSG00000004056 | Akt2 | 0.116986421 | 6.535980267 | 0.039000548 |
| ENSMUSG00000020974 | Pole2 | -0.327114359 | 1.78264647 | 0.039033519 |
| ENSMUSG00000041426 | Hibch | 0.191204089 | 4.923506949 | 0.039035331 |
| ENSMUSG00000032786 | Alas1 | -0.143721905 | 8.801682711 | 0.039145529 |
| ENSMUSG00000039105 | Atp6v1g1 | -0.155538149 | 5.813035233 | 0.039168017 |
| ENSMUSG00000040447 | Spns2 | 0.217980977 | 4.623891708 | 0.039187452 |
| ENSMUSG00000020029 | Nudt4 | 0.184096373 | 6.81226729 | 0.039194663 |
| ENSMUSG00000016128 | Stard13 | 0.223058933 | 4.072709424 | 0.039256535 |
| ENSMUSG00000015957 | Wnt11 | 0.457547187 | 1.861341365 | 0.039457421 |
| ENSMUSG00000028633 | Ctps | 0.159309335 | 4.823160636 | 0.039548705 |
| ENSMUSG00000039831 | Arhgap29 | 0.217816695 | 6.44857077 | 0.039554987 |
| ENSMUSG00000026043 | Col3a1 | -0.212611542 | 12.00576245 | 0.039571145 |
| ENSMUSG00000047583 | Tyw3 | 0.262498243 | 2.234472674 | 0.039641617 |
| ENSMUSG00000042644 | Itpr3 | -0.155039752 | 5.084456991 | 0.039739369 |
| ENSMUSG00000026649 | Cfap126 | 0.255264523 | 2.571077396 | 0.039786689 |
| ENSMUSG00000002475 | Abhd3 | -0.244488322 | 2.523375831 | 0.039794935 |
| ENSMUSG00000041765 | Ubac2 | 0.112276817 | 5.540531335 | 0.039857165 |
| ENSMUSG00000064225 | Paqr9 | 0.200669295 | 6.550030695 | 0.039909521 |
| ENSMUSG00000033377 | Palmd | 0.261612078 | 6.87719806 | 0.03994984 |
| ENSMUSG00000028359 | Orm3 | -0.49742485 | 0.67719586 | 0.039980216 |
| ENSMUSG00000023307 | March5 | 0.140414078 | 5.809032192 | 0.040033697 |
| ENSMUSG00000034686 | Prr7 | 0.429204993 | 0.745123695 | 0.040048179 |
| ENSMUSG00000041757 | Plekha6 | -0.240166823 | 5.005307229 | 0.040172998 |
| ENSMUSG00000020744 | Slc25a19 | 0.172608771 | 6.349705231 | 0.040298392 |
| ENSMUSG00000008475 | Arpc5 | -0.113200989 | 8.218935956 | 0.040300728 |
| ENSMUSG00000048546 | Tob2 | 0.152889484 | 5.824377969 | 0.040382433 |
| ENSMUSG00000025764 | Jade1 | 0.144823363 | 4.320285178 | 0.040449862 |
| ENSMUSG00000040213 | Kyat3 | 0.265950096 | 2.62927142 | 0.040461187 |
| ENSMUSG00000040717 | Il17rd | 0.277734444 | 2.482966809 | 0.040531132 |
| ENSMUSG00000038695 | Josd2 | -0.143039054 | 5.423353155 | 0.040588663 |
| ENSMUSG00000068794 | Col28a1 | 0.376845977 | 2.851868345 | 0.040692092 |
| ENSMUSG00000023044 | Csad | 0.142445917 | 6.157818335 | 0.040696207 |
| ENSMUSG00000031714 | Gab1 | 0.147670106 | 4.587877095 | 0.040713025 |
| ENSMUSG00000003355 | Fkbp11 | -0.236507548 | 2.895017759 | 0.040722982 |
| ENSMUSG00000020875 | Hoxb9 | 0.486060699 | 0.627932758 | 0.04075882 |
| ENSMUSG00000020003 | Pex7 | 0.170531115 | 4.743557537 | 0.04076598 |
| ENSMUSG00000025608 | Podxl | 0.210083202 | 5.218253073 | 0.040773457 |
| ENSMUSG00000020439 | Smtn | 0.149553466 | 5.792526492 | 0.040777083 |
| ENSMUSG00000025010 | Ccnj | 0.293075198 | 2.155370469 | 0.040784841 |
| ENSMUSG00000108624 | Gm45091 | 0.411383891 | 0.881173507 | 0.040823843 |
| ENSMUSG00000027012 | Dync1i2 | -0.111615114 | 7.337390629 | 0.040916689 |
| ENSMUSG00000042306 | S100a14 | 0.736674488 | -0.56875997 | 0.040937941 |
| ENSMUSG00000110755 | BC049987 | 0.704186694 | -0.6275163 | 0.041082214 |
| ENSMUSG00000020395 | Itk | -0.379679194 | 1.230882103 | 0.041094013 |
| ENSMUSG00000032294 | Pkm | -0.14713959 | 9.89854324 | 0.041123771 |
| ENSMUSG00000022749 | Tbc1d23 | -0.133871692 | 5.790490015 | 0.041154016 |
| ENSMUSG00000046312 | AI464131 | 0.243974562 | 4.22669854 | 0.041167269 |
| ENSMUSG00000024164 | C3 | 0.17653454 | 10.66546427 | 0.041190546 |
| ENSMUSG00000066361 | Serpina3c | 0.279327501 | 6.354480435 | 0.041260308 |
| ENSMUSG00000002718 | Cse1l | -0.151318856 | 5.003340156 | 0.041290763 |
| ENSMUSG00000025381 | Cnpy2 | -0.122623045 | 6.3773365 | 0.041321929 |
| ENSMUSG00000054723 | Vmac | 0.172946754 | 4.140696839 | 0.041378865 |
| ENSMUSG00000026065 | Slc9a4 | -0.247998648 | 3.000889838 | 0.041468349 |
| ENSMUSG00000042349 | Ikbke | -0.132153924 | 6.88485589 | 0.041499964 |
| ENSMUSG00000031149 | Praf2 | -0.237062941 | 3.17625132 | 0.041608013 |
| ENSMUSG00000024768 | Lipf | 0.293337338 | 3.765472343 | 0.041706999 |
| ENSMUSG00000052298 | Cdc42se2 | -0.111457425 | 6.936164958 | 0.041744261 |
| ENSMUSG00000040605 | Bace2 | 0.208308225 | 3.223256178 | 0.041770454 |
| ENSMUSG00000019947 | Arid5b | 0.145680371 | 4.69517339 | 0.041787821 |
| ENSMUSG00000074505 | Fat3 | -0.281945724 | 3.446873419 | 0.041795088 |
| ENSMUSG00000055320 | Tead1 | 0.214720956 | 4.975789409 | 0.04179802 |
| ENSMUSG00000021241 | Isca2 | 0.1356902 | 5.661040667 | 0.041857488 |
| ENSMUSG00000067577 | A430093F15Rik | 0.680905522 | -0.466051354 | 0.041898128 |
| ENSMUSG00000037826 | Ppm1k | 0.304460704 | 2.071340443 | 0.042010181 |
| ENSMUSG00000036046 | 5031439G07Rik | -0.127817693 | 8.637828103 | 0.042027822 |
| ENSMUSG00000035969 | Rusc2 | -0.125177263 | 6.517104731 | 0.042253268 |
| ENSMUSG00000021759 | Plpp1 | 0.144026734 | 6.078495105 | 0.042258857 |
| ENSMUSG00000042215 | Bag2 | -0.22178541 | 3.462980701 | 0.042317144 |
| ENSMUSG00000031843 | Mphosph6 | -0.193720513 | 3.590687775 | 0.042343263 |
| ENSMUSG00000032348 | Gsta4 | 0.36584524 | 1.593498778 | 0.042371894 |
| ENSMUSG00000035165 | Kcne3 | -0.342630395 | 1.506315088 | 0.04238243 |
| ENSMUSG00000032350 | Gclc | -0.159244405 | 5.192679156 | 0.04240337 |
| ENSMUSG00000037972 | Snn | 0.169001599 | 5.061440247 | 0.042426135 |
| ENSMUSG00000068747 | Sort1 | 0.098935282 | 7.80245641 | 0.042457291 |
| ENSMUSG00000036854 | Hspb6 | 0.186741237 | 4.816789969 | 0.042461012 |
| ENSMUSG00000057286 | St6galnac2 | 0.198167378 | 3.780157975 | 0.042479212 |
| ENSMUSG00000051107 |  | -0.384889646 | 1.879743818 | 0.04249443 |
| ENSMUSG00000001036 | Epn2 | 0.169320956 | 5.85580075 | 0.042637309 |
| ENSMUSG00000035283 | Adrb1 | 0.518302559 | 0.37781988 | 0.042650601 |
| ENSMUSG00000073481 | Marc2 | 0.117958151 | 7.273954438 | 0.042750891 |
| ENSMUSG00000032549 | Rab6b | -0.191735317 | 4.520629521 | 0.042762415 |
| ENSMUSG00000057337 | Chst3 | -0.258509955 | 2.642197726 | 0.04276482 |
| ENSMUSG00000020258 | Glyctk | 0.276313451 | 3.120327346 | 0.042916945 |
| ENSMUSG00000044749 | Abca6 | 0.312942225 | 2.045435986 | 0.042939714 |
| ENSMUSG00000024691 | Fam111a | -0.18392296 | 6.00524687 | 0.04302052 |
| ENSMUSG00000024782 | Ak3 | 0.142250857 | 7.219640519 | 0.043041503 |
| ENSMUSG00000040289 | Hey1 | 0.26656114 | 2.757671824 | 0.043103769 |
| ENSMUSG00000021732 | Fgf10 | -0.261087336 | 3.55052519 | 0.043127748 |
| ENSMUSG00000026385 | Dbi | 0.157125193 | 10.11493103 | 0.043133654 |
| ENSMUSG00000006342 | Susd2 | 0.232926897 | 3.543143544 | 0.043151127 |
| ENSMUSG00000091387 | Gcnt4 | -0.510023443 | 0.39568465 | 0.043183802 |
| ENSMUSG00000045092 | S1pr1 | 0.16964986 | 6.016672703 | 0.043205213 |
| ENSMUSG00000034765 | Dusp5 | -0.214164449 | 4.457049162 | 0.043271744 |
| ENSMUSG00000019889 | Ptprk | 0.283684105 | 3.888539347 | 0.043317281 |
| ENSMUSG00000021624 | Cd180 | -0.120049702 | 6.905522702 | 0.043323179 |
| ENSMUSG00000032387 | Rbpms2 | 0.31432328 | 3.62777901 | 0.043367984 |
| ENSMUSG00000099137 | Gm10603 | 0.393952095 | 1.092487116 | 0.043391029 |
| ENSMUSG00000057147 | Dph6 | -0.188722922 | 3.40012655 | 0.043531732 |
| ENSMUSG00000022186 | Oxct1 | 0.120335193 | 7.640081751 | 0.043546633 |
| ENSMUSG00000034116 | Vav1 | -0.108896022 | 6.740936288 | 0.043565342 |
| ENSMUSG00000015850 | Adamtsl4 | 0.228862544 | 4.215661388 | 0.043600362 |
| ENSMUSG00000043733 | Ptpn11 | 0.125895564 | 6.936628617 | 0.043619705 |
| ENSMUSG00000025413 | Ttc4 | -0.118138047 | 5.32060233 | 0.043627384 |
| ENSMUSG00000022408 | Fam83f | -0.172080004 | 5.348939282 | 0.043629708 |
| ENSMUSG00000001506 | Col1a1 | -0.19186569 | 10.86272083 | 0.043650463 |
| ENSMUSG00000021072 | Tmx1 | -0.13507785 | 5.201472908 | 0.043675409 |
| ENSMUSG00000020777 | Acox1 | 0.141547976 | 8.403456459 | 0.043701044 |
| ENSMUSG00000022946 | Dop1b | -0.12268278 | 6.699368221 | 0.043717845 |
| ENSMUSG00000032122 | Slc37a2 | -0.12060392 | 8.780093779 | 0.043787498 |
| ENSMUSG00000028251 | Tstd3 | -0.153710628 | 4.45404632 | 0.043810624 |
| ENSMUSG00000047866 | Lonp2 | 0.129841187 | 6.880601214 | 0.043815149 |
| ENSMUSG00000022987 | Zfp641 | 0.380650072 | 1.432155798 | 0.043886762 |
| ENSMUSG00000034744 | Nagk | -0.116024187 | 7.199595584 | 0.043933996 |
| ENSMUSG00000048078 | Tenm4 | 0.243035824 | 6.793812046 | 0.043956519 |
| ENSMUSG00000070044 | Fam149a | 0.163772127 | 4.540154044 | 0.043975782 |
| ENSMUSG00000065922 | n-R5-8s1 | -0.680880786 | 1.414575062 | 0.04399746 |
| ENSMUSG00000028465 | Tln1 | -0.104543631 | 9.835968703 | 0.044040485 |
| ENSMUSG00000070390 | Nlrp1b | -0.19157049 | 3.912196467 | 0.044045085 |
| ENSMUSG00000042439 | Zfp532 | 0.156285511 | 4.228613935 | 0.044087584 |
| ENSMUSG00000115219 | Eef1akmt4 | -0.208385424 | 3.055080912 | 0.044159106 |
| ENSMUSG00000003444 | Med29 | -0.201471018 | 3.348092759 | 0.044200483 |
| ENSMUSG00000031775 | Pllp | 0.359634937 | 2.053802535 | 0.044206464 |
| ENSMUSG00000043542 | Zc2hc1a | 0.308099684 | 3.969494743 | 0.044280084 |
| ENSMUSG00000021365 | Nedd9 | 0.170903432 | 5.908155275 | 0.044300965 |
| ENSMUSG00000022841 | Ap2m1 | -0.110417026 | 6.368737304 | 0.044327145 |
| ENSMUSG00000025422 | Agap2 | 0.247255067 | 2.969358737 | 0.044364456 |
| ENSMUSG00000040856 | Dlk1 | 0.651351334 | -0.264287609 | 0.044392485 |
| ENSMUSG00000031959 | Wdr59 | -0.170579978 | 3.884392697 | 0.044497936 |
| ENSMUSG00000085939 | Cd63-ps | -0.38182442 | 2.163426157 | 0.044524653 |
| ENSMUSG00000045377 | Tmem88 | 0.23465571 | 4.331448667 | 0.044596393 |
| ENSMUSG00000026893 | Gca | 0.28055424 | 1.765598063 | 0.044622933 |
| ENSMUSG00000018841 | Rad51d | 0.142592215 | 4.415286478 | 0.044650913 |
| ENSMUSG00000043391 | 2510009E07Rik | -0.145714383 | 4.9987557 | 0.044682892 |
| ENSMUSG00000044548 | Dact1 | 0.234286149 | 3.268809077 | 0.044706975 |
| ENSMUSG00000018326 | Ywhab | 0.126015538 | 8.727595113 | 0.044778463 |
| ENSMUSG00000031609 | Sap30 | -0.17711 | 3.929081288 | 0.044780651 |
| ENSMUSG00000022099 | Dmtn | 0.301248303 | 4.467053154 | 0.044800388 |
| ENSMUSG00000074657 | Kif5a | 0.212092062 | 2.901522393 | 0.044838317 |
| ENSMUSG00000019297 | Nop9 | -0.113312457 | 5.123378876 | 0.044852941 |
| ENSMUSG00000027514 | Zbp1 | 0.238211122 | 4.443416838 | 0.044882412 |
| ENSMUSG00000054619 | Mettl7a1 | 0.173899015 | 6.142624322 | 0.044978005 |
| ENSMUSG00000051339 | 2900026A02Rik | 0.183704032 | 4.853869966 | 0.045028418 |
| ENSMUSG00000002308 | Cd320 | 0.183992992 | 5.293500683 | 0.045152303 |
| ENSMUSG00000028470 | Hint2 | 0.147522965 | 5.171893316 | 0.045152682 |
| ENSMUSG00000028678 | Kif2c | -0.190874959 | 3.588351488 | 0.045211879 |
| ENSMUSG00000048612 | Myof | -0.121402725 | 8.195063214 | 0.045241423 |
| ENSMUSG00000027809 | Etfdh | 0.130475921 | 6.575840693 | 0.045304979 |
| ENSMUSG00000021903 | Galnt15 | 0.204766678 | 5.70649795 | 0.045312623 |
| ENSMUSG00000042498 | D330045A20Rik | -0.577493326 | 0.062619847 | 0.045454797 |
| ENSMUSG00000091575 | 2010016I18Rik | -0.374801089 | 1.33799965 | 0.045500909 |
| ENSMUSG00000035439 | Haus8 | -0.156231787 | 4.977898854 | 0.045527977 |
| ENSMUSG00000028030 | Tbck | -0.152010295 | 4.143388825 | 0.045533159 |
| ENSMUSG00000058022 | Adtrp | 0.341757381 | 2.309063261 | 0.045564397 |
| ENSMUSG00000087574 | C030037D09Rik | 0.550745559 | -0.020135069 | 0.045585423 |
| ENSMUSG00000040723 | Rcsd1 | 0.127284192 | 5.629686707 | 0.045619282 |
| ENSMUSG00000085741 | 5430405H02Rik | 0.230722386 | 2.437661163 | 0.045663144 |
| ENSMUSG00000020799 | Tekt1 | 0.411164358 | 1.515525825 | 0.045687234 |
| ENSMUSG00000030108 | Slc6a13 | 0.201023058 | 6.339311721 | 0.045692595 |
| ENSMUSG00000073176 | Zfp449 | 0.336528921 | 1.682916148 | 0.045776918 |
| ENSMUSG00000006800 | Sulf2 | 0.135535487 | 7.682821833 | 0.045855815 |
| ENSMUSG00000014301 | Pam16 | -0.537227144 | 0.440696303 | 0.045862584 |
| ENSMUSG00000046844 | Vat1l | 0.785902244 | 0.197944275 | 0.045869234 |
| ENSMUSG00000035828 | Pim3 | 0.219279549 | 6.346973006 | 0.045928194 |
| ENSMUSG00000041346 | Wrap53 | 0.167362697 | 3.842022167 | 0.045934734 |
| ENSMUSG00000035948 | Acss3 | 0.315029393 | 3.131535051 | 0.046000797 |
| ENSMUSG00000026627 | Tmem206 | -0.168070649 | 5.789051597 | 0.046038193 |
| ENSMUSG00000049807 | Arhgap23 | 0.13117074 | 6.311784087 | 0.046059506 |
| ENSMUSG00000024970 | Spindoc | 0.142013579 | 4.902438366 | 0.046068632 |
| ENSMUSG00000036672 | Cenpt | -0.124142285 | 4.774359287 | 0.046270562 |
| ENSMUSG00000028763 | Hspg2 | 0.149441271 | 10.08845338 | 0.046336401 |
| ENSMUSG00000001014 | Icam4 | -0.742859579 | -0.242922792 | 0.04634906 |
| ENSMUSG00000027248 | Pdia3 | -0.112247105 | 9.14501557 | 0.046356575 |
| ENSMUSG00000019768 | Esr1 | 0.287172397 | 2.579981705 | 0.04641325 |
| ENSMUSG00000082361 | Btc | 0.449269819 | 1.132576733 | 0.046531143 |
| ENSMUSG00000032598 | Nckipsd | 0.133926451 | 5.23247828 | 0.046551369 |
| ENSMUSG00000074364 | Ehd2 | 0.174611912 | 9.145996286 | 0.046614026 |
| ENSMUSG00000027602 | Map1lc3a | 0.161678892 | 7.391737565 | 0.046636782 |
| ENSMUSG00000039013 | Siglecf | -0.463752389 | 0.223618587 | 0.046743044 |
| ENSMUSG00000072941 | Sod3 | 0.220494291 | 9.359623045 | 0.046847863 |
| ENSMUSG00000006010 | Odr4 | 0.137815168 | 5.45955758 | 0.046912732 |
| ENSMUSG00000022231 | Sema5a | 0.289075365 | 2.785934338 | 0.04694124 |
| ENSMUSG00000045917 | Tmem268 | 0.139786179 | 4.82885981 | 0.047021061 |
| ENSMUSG00000051235 | Gen1 | -0.420030389 | 1.201107 | 0.04707929 |
| ENSMUSG00000029661 | Col1a2 | -0.196048261 | 10.80736851 | 0.047107645 |
| ENSMUSG00000063060 | Sox7 | 0.297257723 | 3.289587924 | 0.047151316 |
| ENSMUSG00000070565 | Rasal2 | 0.215404804 | 3.209332833 | 0.047159244 |
| ENSMUSG00000051041 | Olfml1 | 0.19828009 | 4.026752821 | 0.04727286 |
| ENSMUSG00000032265 | Tent5a | 0.200631985 | 4.979347444 | 0.047330422 |
| ENSMUSG00000004268 | Emg1 | -0.102306148 | 5.707752315 | 0.047343944 |
| ENSMUSG00000026475 | Rgs16 | -0.377964787 | 2.534990899 | 0.047502056 |
| ENSMUSG00000097150 | Gm26513 | 0.744435843 | -0.730448427 | 0.047537057 |
| ENSMUSG00000031885 | Cbfb | -0.135870101 | 5.106092248 | 0.047603736 |
| ENSMUSG00000031849 | Comp | 0.497157698 | 3.575906195 | 0.047608815 |
| ENSMUSG00000034024 | Cct2 | -0.090698245 | 7.769117038 | 0.047616901 |
| ENSMUSG00000098973 | Mir6236 | -0.640479895 | 4.821413575 | 0.047656637 |
| ENSMUSG00000033059 | Pygb | 0.128082151 | 6.729787158 | 0.047749434 |
| ENSMUSG00000040957 | Cables1 | 0.175414872 | 3.790207327 | 0.047828629 |
| ENSMUSG00000028487 | Bnc2 | 0.382056536 | 0.800912531 | 0.047881739 |
| ENSMUSG00000056671 | Prelid2 | -0.26505373 | 2.096718786 | 0.047888636 |
| ENSMUSG00000027854 | Sike1 | -0.120252647 | 5.429919926 | 0.047975864 |
| ENSMUSG00000028681 | Ptch2 | 0.316606115 | 3.329357508 | 0.047978755 |
| ENSMUSG00000006519 | Cyba | -0.174357723 | 9.735093978 | 0.048013043 |
| ENSMUSG00000024764 | Naa40 | -0.124115017 | 5.374119289 | 0.048026586 |
| ENSMUSG00000029171 | Pgm2 | -0.145731116 | 4.736741981 | 0.048070878 |
| ENSMUSG00000041445 | Mmrn2 | 0.185398228 | 5.731707969 | 0.04807648 |
| ENSMUSG00000021555 | Naa35 | -0.127511551 | 4.841451327 | 0.048118416 |
| ENSMUSG00000025006 | Sorbs1 | 0.217785488 | 6.114383058 | 0.048290818 |
| ENSMUSG00000041202 | Pla2g2d | 0.402338449 | 1.178934684 | 0.048316621 |
| ENSMUSG00000106383 | E330034L11Rik | -0.365954246 | 1.217372849 | 0.048375949 |
| ENSMUSG00000060376 | Bckdha | 0.130224812 | 7.089179203 | 0.048383851 |
| ENSMUSG00000007950 | Abhd8 | -0.147599313 | 4.372555308 | 0.048401814 |
| ENSMUSG00000058979 | Hdhd5 | -0.142255838 | 4.092493621 | 0.048404757 |
| ENSMUSG00000026707 | Nsun6 | 0.24330775 | 2.600451964 | 0.048423156 |
| ENSMUSG00000022853 | Ehhadh | 0.181573728 | 4.226373191 | 0.048435561 |
| ENSMUSG00000089665 | Fcor | 0.210800825 | 5.290177092 | 0.048581851 |
| ENSMUSG00000073758 | Sh3d21 | 0.288069541 | 1.941724663 | 0.048642487 |
| ENSMUSG00000058743 | Kcnj14 | 0.265091425 | 3.351928819 | 0.048779409 |
| ENSMUSG00000019312 | Grb7 | 0.493998777 | 0.711990096 | 0.048849366 |
| ENSMUSG00000022000 | Zc3h13 | -0.129380775 | 5.060023713 | 0.048916844 |
| ENSMUSG00000022773 | Ypel1 | -0.369667811 | 1.061601592 | 0.048930268 |
| ENSMUSG00000035275 | Raver2 | 0.332660813 | 2.170712435 | 0.048998015 |
| ENSMUSG00000097207 | 6030443J06Rik | 0.766753187 | -0.683513912 | 0.049013603 |
| ENSMUSG00000015747 | Vps45 | -0.115060538 | 4.836011796 | 0.04905242 |
| ENSMUSG00000017830 | Dhx58 | 0.137291613 | 4.886539476 | 0.049145479 |
| ENSMUSG00000027978 | Prss12 | -0.236429522 | 3.097370459 | 0.049164098 |
| ENSMUSG00000100691 | 2010320M18Rik | 0.205961306 | 3.306144324 | 0.049166409 |
| ENSMUSG00000025213 | Kazald1 | -0.274613133 | 2.222693559 | 0.049182994 |
| ENSMUSG00000023942 | Slc29a1 | -0.136621369 | 7.286435361 | 0.049200253 |
| ENSMUSG00000110647 | Gm17745 | -0.41357809 | 0.867831609 | 0.049283451 |
| ENSMUSG00000037946 | Fgd3 | -0.14319519 | 5.869635978 | 0.049305168 |
| ENSMUSG00000041707 | Tmem273 | -0.233512481 | 3.744544937 | 0.049328333 |
| ENSMUSG00000069835 | Sat2 | 0.191603675 | 2.907242986 | 0.049374836 |
| ENSMUSG00000112302 | Gm48226 | 0.335512236 | 1.588087333 | 0.04944281 |
| ENSMUSG00000026536 | Ifi211 | 0.248930062 | 3.144755638 | 0.049462662 |
| ENSMUSG00000026303 | Mlph | -0.341334456 | 1.368191718 | 0.049624809 |
| ENSMUSG00000060961 | Slc4a4 | 0.289643255 | 2.573090462 | 0.049691438 |
| ENSMUSG00000040410 | Fbxl4 | 0.207703991 | 4.607185032 | 0.04969946 |
| ENSMUSG00000091898 | Tnnc1 | -0.442810103 | 0.723049971 | 0.04980579 |
| ENSMUSG00000031488 | Rab11fip1 | -0.188710368 | 3.296848049 | 0.049863634 |
| ENSMUSG00000058638 | Zfp110 | 0.132319462 | 4.940115132 | 0.049887749 |
| ENSMUSG00000074336 | Apoc4 | 0.274523984 | 2.64300411 | 0.049984426 |
