# Supplementary material for: Accelerated phosphatidylcholine turnover in macrophages promotes adipose tissue inflammation in obesity
Source: eLife. 2019 Aug 16;8:e47990. doi: 10.7554/eLife.47990 (PMC6748830; doi:10.7554/eLife.47990)
Supplement: Supplementary file 3. — FAM/TAMRA reporter and quencher detection system was used for genes with indicated probe sequences, and SYBR was used for the remaining genes. [file elife-47990-supp3.docx]

**Supplementary file 3.**

| **Gene** | **Forward primer (5’-3’)** | **Reverse primer (5’-3’)** | **Probe (5’-3’)** |
| --- | --- | --- | --- |
| *18s* | CGGCTACCACATCCAAGGAA | GCTGGAATTACCGCGGCT | GAGGGCAAGTCTGGTGCCAG |
| *36b4* | AGATGCAGCAGATCCGCAT | GTTCTTGCCCATCAGCACC |  |
| *ACTB*  *(human)* | TGAGATGCGTTGTTACAGGAAGTC | GACTGGGCCATTCTCCTTAGAGA | CTTGCCATCCTAAAAGCCACCCCACTT |
| *Arg1* | CTCCAAGCCAAAGTCCTTAGAG | AGGAGCTGTCATTAGGGACATC |  |
| *Atf4* | ATGATGGCTTGGCCAGTG | CCATTTTCTCCAACATCCAATC |  |
| *B2M*  *(human)* | CGCTCCGTGGCCTTAGC | AATCTTTGGAGTACGCTGGATAGC | TGCTCGCGCTACTCTCTCTTTCTGGC |
| *Hspa5* | CTGAGGCGTATTTGGGAAAG | TCATGACATTCAGTCCAGCAA |  |
| *Ddit3* | CCACCACACCTGAAAGCAGAA | AGGTGAAAGGCAGGGACTCA |  |
| *Elovl5* | TTCGATGCGTCACTCAGTACCT | TGTCCAGGAGGAACCATCCTT |  |
| *Elovl6* | TGCAGGAAAACTGGAAGAAGTCT | ATGCCGACCACCAAAGATAAA |  |
| *Emr1* | CAGATACAGCAATGCCAAGCA | GATTGTGAAGGTAGCATTCACAAGTG |  |
| *ERdj4* | CACAAAGATGCCTTTTCTACCG | TTAAACTTTTCAGCTTAATGACGTG |  |
| *Fads1* | TGCACCCCCTCTTCTTCGCC | AGGCTGGGGGTCCGATGAGG |  |
| *Fads2* | AGCCCTGGTTTTCCTCAACT | GTTGTGACGTGGCATAGTGG |  |
| *Fasn* | GCCCAGACAGAGAAGAGGCA | CTGACTCGGGCAACTTCCC | GGAGGAGGTGGTGATAGCCGGTATGTC |
| *Fatp1* | CGTTTCGATGGTTATGTTAGTGACA | CATCACTAGCACGTCACCTGAGA |  |
| *GAPDH*  *(human)* | CCAGGTGGTCTCCTCTGACTTC | TCATACCAGGAAATGAGCTTGACA | ACAGCGACACCCACTCCTCCACCTT |
| *Glut4* | ACTCATTCTTGGACGGTTCCTC | CACCCCGAAGATGAGTGGG | TGGCGCCTACTCAGGGCTAACATCA |
| *Il10* | CAGAGCCACATGCTCCTAGA | TGTCCAGCTGGTCCTTTGTT |  |
| *Insig1* | GCGCTGTATTGCCGTGTTC | ACAGCTGGACATTATTGGCAAA | CATCAACCACGCCAGTGCCA |
| *Itgam* | CAGACAGGAAGTAGCAGCTCCT | CTGGTCATGTTGATGAAGGTGCT |  |
| *Itgax* | GGCTATCAAGCATGTCATAACAGAAC | CCCCTTGTTTTCTCCCATCAG |  |
| *Mgl1* | AGGTCCCTGTCATGCTTCTG | GCTGCTGGTGATCCTCTTGT |  |
| *Mrc1* | GCATGGGTTTTACTGCTACTTGATT | CAGGAATGCTTGTTCATATCTGTCTT |  |
| *Pcyt1a* | TCTGCAGGGAGCGATGATG | TGTGGAGATACCTTCTGTCCTCTGT | TATAAGCACATCAAGGACGCAGGCATGTT |
| *PCYT1a*  *(human)* | Commercial assay (Hs00192339_m1, Thermofisher) | | |
| *Pcyt1b* | CAGTGCCAAGCACCTCATGA | GGCCTATCAACTGGTGTTCCTAA |  |
| *POLR2a*  *(human)* | GCTATAAGGTGGAACGGCACAT | ACCCGATGCCCCATCAT | AACCGGCAGCCAACTCTGCACAA |
| *Scd1* | CTTGCGGATCTTCCTTATCATT | GATCTCGGGCCCATTCG | ACCATGGCGTTCCAGAATGACGTGT |
| *Scd2* | TGGTTTCCATGGGAGCTG | TTGATGTGCCAGCGGTACT |  |
| *Srebf1* | GCCATGGATTGCACATTTGA | GGCCCGGGAAGTCACTG | GACATGCTCCAGCTCATCAACAACCAAG |
| *Tbp* | CAAACCCAGAATTGTTCTCCTT | ATGTGGTCTTCCTGAATCCCT |  |
| *Tnf* | CATCTTCTCAAAATTCGAGTGACAA | TGGGAGTAGACAAGGTACAACCC | CACGTCGTAGCAAACCACCAAGTGGA |
| *Xbp1 spliced* | AGCTTTTACGGGAGAAAACTCA | GCCTGCACCTGCTGCG |  |
| *Xbp1 total* | ACACGCTTGGGAATGGACAC | CCATGGGAAGATGTTCTGGG |  |
