# Supplementary material for: Accelerated phosphatidylcholine turnover in macrophages promotes adipose tissue inflammation in obesity
Source: eLife. 2019 Aug 16;8:e47990. doi: 10.7554/eLife.47990 (PMC6748830; doi:10.7554/eLife.47990)
Supplement: Transparent reporting form [file elife-47990-transrepform.docx]

***eLife’s* transparent reporting form**

We encourage authors to provide detailed information *within their submission* to facilitate the interpretation and replication of experiments. Authors can upload supporting documentation to indicate the use of appropriate reporting guidelines for health-related research (see [EQUATOR Network](http://www.equator-network.org/%20)), life science research (see the [BioSharing Information Resource](https://biosharing.org/)), or the [ARRIVE guidelines](http://www.plosbiology.org/article/info:doi/10.1371/journal.pbio.1000412) for reporting work involving animal research. Where applicable, authors should refer to any relevant reporting standards documents in this form.

If you have any questions, please consult our Journal Policies and/or contact us: [editorial@elifesciences.org](mailto:editorial@elifesciences.org).

**Sample-size estimation**

- You should state whether an appropriate sample size was computed when the study was being designed
- You should state the statistical method of sample size computation and any required assumptions
- If no explicit power analysis was used, you should describe how you decided what sample (replicate) size (number) to use

Please outline where this information can be found within the submission (e.g., sections or figure legends), or explain why this information doesn’t apply to your submission:

For all animal work, we computed the sample size at the study design stage by performing power calculations: alpha was set at 0.05 and 1-beta at 0.8. For most of our experiments, the effect size was determined based on historical sets of data generated by the TVP Lab, the Disease Model Core (DMC), which is the animal core facility of our department, and the standardized metabolic phenotyping pipeline at the Wellcome Trust Sanger Institute as well as the scientific literature, and discussions with other researchers.

Power analysis was performed using freely available software (Russ Lenth’s power and sample size calculator). We selected a) the appropriate statistical test (in this case we used t-test for comparing macrophage-specific *Pcyt1a* WT and KO mice and reduced ITTs and GTTs to single data points by analysing areas of curves); b) we assumed variability would be similar to our previous experimental data (based on results from the TVP Lab, DMC, WTSI) and; c) we selected a biologically relevant effect size based on previous experimental data, the scientific literature and discussions with other researchers.

For *in vitro* cell work, we did not use statistics to predict sample size. However, we used at least 4 donor mice per group to generate BMDMs in each experiment. Furthermore, as stated below, we reproduced the key *in vitro* findings using independent BMDM cultures from different mice.

**Replicates**

- You should report how often each experiment was performed
- You should include a definition of biological versus technical replication
- The data obtained should be provided and sufficient information should be provided to indicate the number of independent biological and/or technical replicates
- If you encountered any outliers, you should describe how these were handled
- Criteria for exclusion/inclusion of data should be clearly stated
- High-throughput sequence data should be uploaded before submission, with a private link for reviewers provided (these are available from both GEO and ArrayExpress)

Please outline where this information can be found within the submission (e.g., sections or figure legends), or explain why this information doesn’t apply to your submission:

Mouse *in vivo* experiments were performed once. Each biological replicate in the presented in vivo mouse experiments corresponds to individual mice, except in figure 1, containing our previously published microarray and lipidomics analysis of ATMs isolated from WT and ob/ob mice that was generated using pools of 5 mice, therefore 1 biological replicate corresponds to the average of 5 individual animals.

The number of biological replicates in each in vivo study is indicated in the figure legend. In some cases where a figure contains multiple panels or figure supplements related to the same study (for example, qPCR, Western blotting and flow cytometry analysis of adipose tissue of the same animals), the number of replicates is indicated only once in the figure legend, implying that all figure panels were generated using the same number of replicates.

We did not exclude any mice from in vivo experiments due to statistical reasons. One mouse from ob/ob bone marrow transplant experiment had to be culled due to health reasons at the start of experiment, therefore was excluded from the study.

In human ATM gene expression analysis, each replicate corresponds to cells isolated from a different individual.

*In vitro* BMDM experiments were performed as indicated in the legend. We have reproduced every major finding – namely 1) *Pcyt1a* deficient BMDMs demonstrating lower ER stress and inflammation in response to palmitate treatment compared to controls; 2) *Pcyt1a* deficient BMDMs showing no differences in exogenous palmitate incorporation into PC fraction compared to controls; 3) *Pcyt1a* deficient BMDMs having higher PUFA abundance compared to controls – using at least 3 independent BMDM cultures from different mice, and presented one of the experiments that was representative of our findings.

In BMDM experiments, 1 replicate corresponds to cells isolated from 1 individual mouse, differentiated alongside other cells from other mice in that experiment.

We did not exclude any mice from presented BMDM experiments.

**Statistical reporting**

- Statistical analysis methods should be described and justified
- Raw data should be presented in figures whenever informative to do so (typically when N per group is less than 10)
- For each experiment, you should identify the statistical tests used, exact values of N, definitions of center, methods of multiple test correction, and dispersion and precision measures (e.g., mean, median, SD, SEM, confidence intervals; and, for the major substantive results, a measure of effect size (e.g., Pearson's r, Cohen's d)
- Report exact p-values wherever possible alongside the summary statistics and 95% confidence intervals. These should be reported for all key questions and not only when the p-value is less than 0.05.

Please outline where this information can be found within the submission (e.g., sections or figure legends), or explain why this information doesn’t apply to your submission:

A description of how statistical tests were selected and performed can be found in the Methods section.

(For large datasets, or papers with a very large number of statistical tests, you may upload a single table file with tests, Ns, etc., with reference to sections in the manuscript.)

**Group allocation**

- Indicate how samples were allocated into experimental groups (in the case of clinical studies, please specify allocation to treatment method); if randomization was used, please also state if restricted randomization was applied
- Indicate if masking was used during group allocation, data collection and/or data analysis

Please outline where this information can be found within the submission (e.g., sections or figure legends), or explain why this information doesn’t apply to your submission:

Detailed explanation on how purchased ob/ob mice were allocated into two experimental groups can be found in the Methods section.

LysM-Cre *Pcyt1a* floxed mice were born at expected 1:1 Mendelian ratio to *Pcyt1a* floxed controls, so littermates were allocated into two experimental groups based on the genotype. In cases where the whole litter consisted of only 1 genotype, these mice were not used for experiments.

In most cases, data analysis was quantitative, so masking was not required. All animal experiments were performed using mouse IDs only, and genotypes were only checked at the data entry stage after experiment.

**Additional data files (“source data”)**

- We encourage you to upload relevant additional data files, such as numerical data that are represented as a graph in a figure, or as a summary table
- Where provided, these should be in the most useful format, and they can be uploaded as “Source data” files linked to a main figure or table
- Include model definition files including the full list of parameters used
- Include code used for data analysis (e.g., R, MatLab)
- Avoid stating that data files are “available upon request”

Please indicate the figures or tables for which source data files have been provided:

We are submitting raw source data excel file for LC-MS lipidomics of *Pcyt1a*-deficient BMDMs, in both palmitate-treated and basal states (containing peak areas for each lipid species normalized to peak areas of respective internal standards).

ATM microarray dataset (GSE36669) used in Figure 1 is already published and referenced in this manuscript.

We are also submitting an excel sheet containing a list of all genes detected by RNAseq in the eWAT of ob/ob bone marrow transplant mice, with a log (Fold change), log (CPM) and p value indicated for each gene.

We have uploaded raw RNA sequencing data of liver macrophages isolated from WT and ob/ob mice in the NCBI database, under the following accession number: PRJNA541224.
